# Supplementary material for: Multi-omic profiling reveals the endogenous and neoplastic responses to immunotherapies in cutaneous T cell lymphoma
Source: Cell Rep Med. 2024 Apr 25;5(5):101527. doi: 10.1016/j.xcrm.2024.101527 (PMC11148639; doi:10.1016/j.xcrm.2024.101527)
Supplement: Document S1. Figures S1–S8 and Tables S1 and S3–S5 [file mmc1.pdf]

Cell Reports Medicine, Volume *5*

Supplemental information

Multi-omic profiling reveals the endogenous and neoplastic responses to immunotherapies in cutaneous T cell lymphoma

David R. Glass, Koshlan Mayer-Blackwell, Nirasha Ramchurren, K. Rachael Parks, George E. Duran, Anna K. Wright, Armando N. Bastidas Torres, Laura Islas, Youn H. Kim, Steven P. Fling, Michael S. Khodadoust, and Evan W. Newell

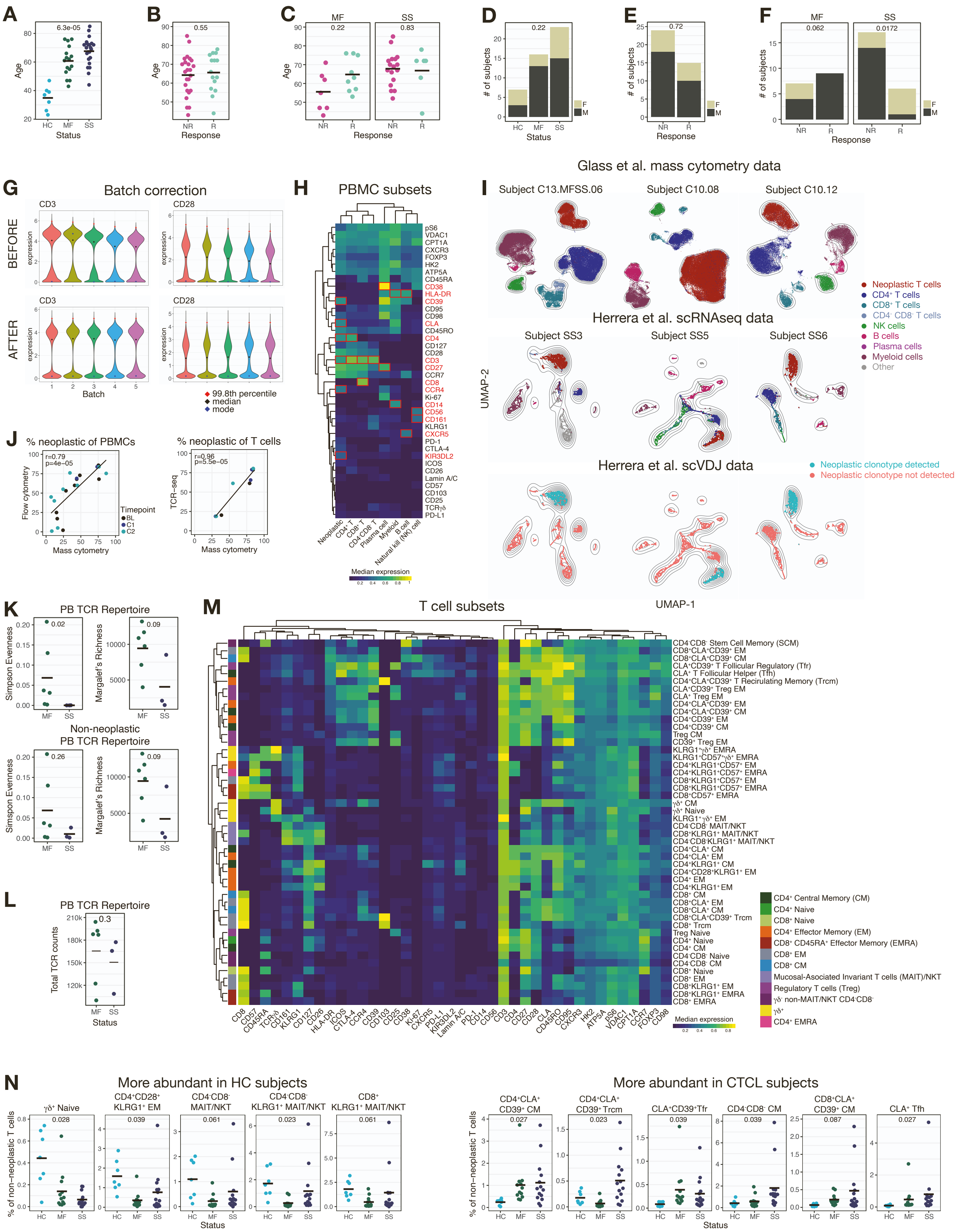
A
Age
HC MF SS
Status
6.3e-05
B
0.55
NR R
Response
C
MF SS
0.22 0.83
D
# of subjects
0.22
F
M
E
0.72
F
MF SS
0.062 0.0172
G
Batch correction
CD3
CD28
BEFORE
AFTER
expression
Batch
99.8th percentile
median
mode
H
PBMC subsets
pS6
VDAC1
CPT1A
CXCR3
FOXP3
HK2
ATP5A
CD45RA
CD38
HLA-DR
CD39
CD95
CD98
CLA
CD45RO
CD4
CD127
CD28
CD3
CD27
CCR7
CD8
CCR4
Ki-67
CD14
CD56
CD161
KLRG1
CXCR5
PD-1
CTLA-4
KIR3DL2
ICOS
CD26
Lamin A/C
CD57
CD103
CD25
TCRγδ
PD-L1
Neoplastic
CD4+ T
CD8+ T
CD4-CD8- T
Plasma cell
Myeloid
B cell
Natural kill (NK) cell
Median expression
I
Glass et al. mass cytometry data
Subject C13.MFSS.06
Subject C10.08
Subject C10.12
Herrera et al. scRNAseq data
Subject SS3
Subject SS5
Subject SS6
Herrera et al. scVDJ data
UMAP-2
UMAP-1
Neoplastic T cells
CD4+ T cells
CD8+ T cells
CD4- CD8- T cells
NK cells
B cells
Plasma cells
Myeloid cells
Other
Neoplastic clonotype detected
Neoplastic clonotype not detected
J
% neoplastic of PBMCs
r=0.79
p=4e-05
Flow cytometry
Mass cytometry
Timepoint
BL
C1
C2
% neoplastic of T cells
r=0.96
p=5.5e-05
TCR-seq
K
PB TCR Repertoire
Simpson Evenness
Margalef's Richness
0.02
0.09
Non-neoplastic
PB TCR Repertoire
Simspon Evenness
0.26
0.09
L
PB TCR Repertoire
Total TCR counts
0.3
Status
M
T cell subsets
CD4-CD8- Stem Cell Memory (SCM)
CD8+CLA+CD39+ EM
CD8+CLA+CD39+ CM
CLA+CD39+ T Follicular Regulatory (Tfr)
CLA+ T Follicular Helper (Tfh)
CD4+CLA+CD39+ T Recirulating Memory (Trcm)
CLA+CD39+ Treg EM
CLA+ Treg EM
CD4+CLA+CD39+ EM
CD4+CLA+CD39+ CM
CD4+CD39+ EM
CD4+CD39+ CM
Treg CM
CD39+ Treg EM
KLRG1+γδ+ EMRA
KLRG1+CD57+γδ+ EMRA
CD4+KLRG1+CD57+ EM
CD4+KLRG1+CD57+ EMRA
CD8+KLRG1+CD57+ EM
CD8+KLRG1+CD57+ EMRA
CD8+CD57+ EMRA
γδ+ CM
γδ+ Naive
KLRG1+γδ+ EM
CD4-CD8- MAIT/NKT
CD8+KLRG1+ MAIT/NKT
CD4-CD8-KLRG1+ MAIT/NKT
CD4+CLA+ CM
CD4+CLA+ EM
CD4+KLRG1+ CM
CD4+CD28+KLRG1+ EM
CD4+ EM
CD4+KLRG1+ EM
CD8+ CM
CD8+CLA+ EM
CD8+CLA+ CM
CD8+CLA+CD39+ Trcm
CD8+ Trcm
Treg Naive
CD4+ Naive
CD4+ CM
CD4-CD8- Naive
CD4-CD8- CM
CD8+ Naive
CD8+ EM
CD8+KLRG1+ EM
CD8+KLRG1+ EMRA
CD8+ EMRA
CD4+ Central Memory (CM)
CD4+ Naive
CD8+ Naive
CD4+ Effector Memory (EM)
CD8+ CD45RA+ Effector Memory (EMRA)
CD8+ EM
CD8+ CM
Mucosal-Associated Invariant T cells (MAIT)/NKT
Regulatory T cells (Treg)
γδ- non-MAIT/NKT CD4-CD8-
γδ+
CD4+ EMRA
Median expression
N
More abundant in HC subjects
γδ+ Naive
0.028
CD4+CD28+ KLRG1+ EM
0.039
CD4-CD8- MAIT/NKT
0.061
CD4-CD8- KLRG1+ MAIT/NKT
0.023
CD8+ KLRG1+ MAIT/NKT
0.061
% of non-neoplastic T cells
Status
More abundant in CTCL subjects
CD4+CLA+ CD39+ CM
0.027
CD4+CLA+ CD39+ Trcm
0.023
CLA+CD39+Tfr
0.039
CD4-CD8- CM
0.039
CD8+CLA+ CD39+ CM
0.087
CLA+ Tfh
0.027

Figure S1: Quality control, clustering, and disease status differences – related to Figure 1

A) Subject age by disease status. Crossbar indicates mean. Kruskal-Wallis.
B) Subject age by response. Crossbar indicates mean. Wilcoxon rank sum.
C) Subject age by response and disease status. Crossbar indicates mean. FDR-corrected Wilcoxon rank sum.
D) Subject gender by disease status. Fisher's exact.
E) Subject gender by response. Fisher's exact.
F) Subject gender by response and disease status. FDR-corrected Fisher's exact.
G) Exemplative batch correction of three protein channels. Violins represent cell distributions from a common control sample run with each batch.
H) Median expression of proteins by cell types from an equal sampling of subjects at baseline. Canonical markers highlighted in red.
I) UMAPs of individual CTCL subjects in this study (top row) or a public scRNAseq dataset (middle, bottom rows) colored by cell type (top, middle rows) or by the detection of the neoplastic TCR clone (bottom row).
J) % neoplastic T cells of PBMCs or of total T cells by clinical flow cytometry, mass cytometry, and TCR sequencing. Fitting lines calculated by linear regression. R- and p-values calculated by Pearson method.
K) Evenness and richness scores of BL TCR repertoires by status with or without neoplastic T cell clones. Wilcoxon rank sum.
L) BL TCR counts as calculated by Adaptive Biotechnology template counting by disease status. Crossbar indicates mean. Wilcoxon rank sum.
M) Median expression of proteins by non-neoplastic T cell clusters, from an equal sampling of subjects at BL.
N) Percent of T cell clusters out of non-neoplastic T cells at baseline, separated by disease status. Crossbar indicates mean. FDR-corrected Kruskal-Wallis.

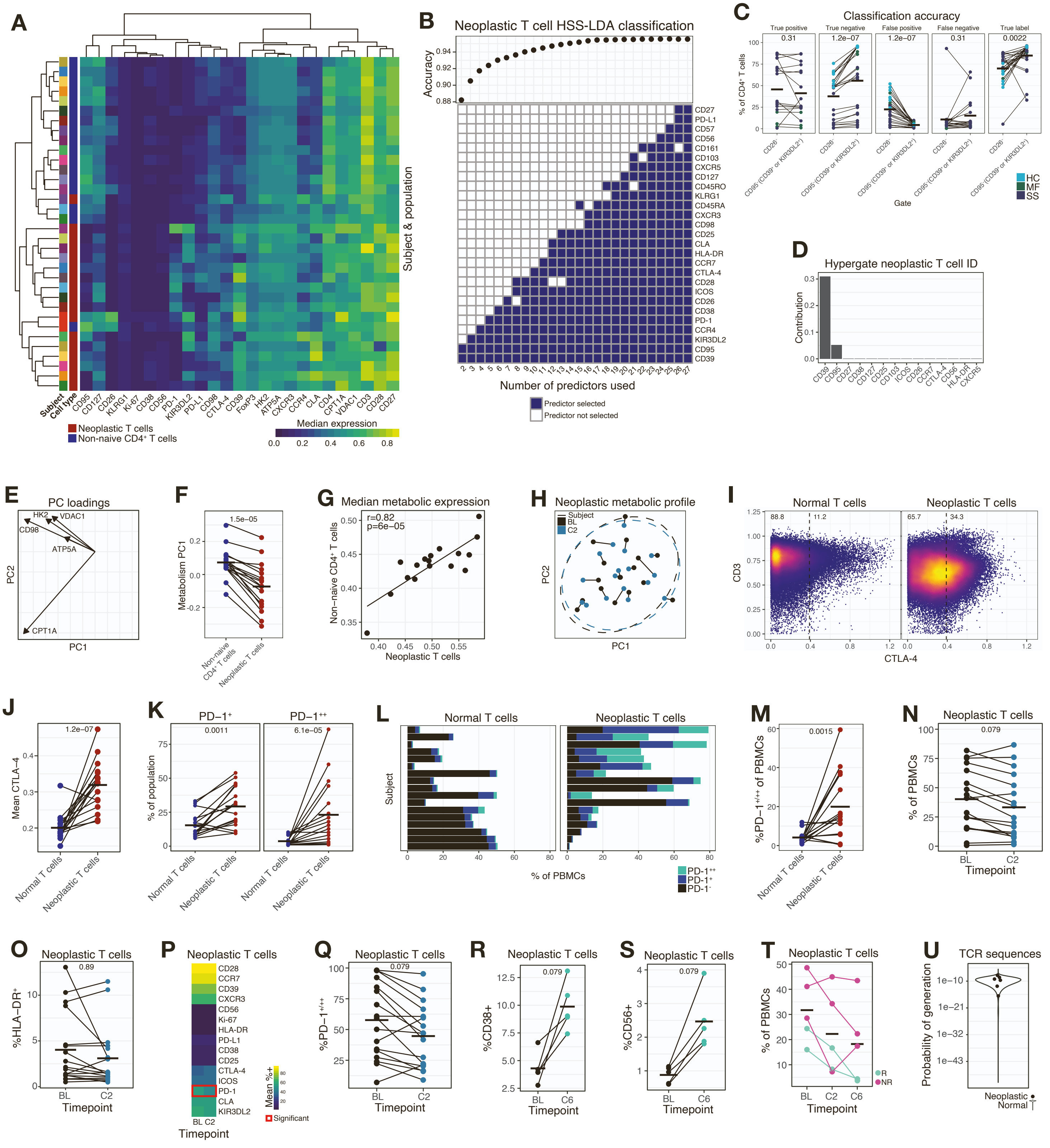

A
Subject & population
Subject
Cell type
Neoplastic T cells
Non-naive CD4+ T cells
Median expression
B
Neoplastic T cell HSS-LDA classification
Accuracy
Number of predictors used
Predictor selected
Predictor not selected
C
Classification accuracy
True positive
True negative
False positive
False negative
True label
% of CD4+ T cells
Gate
HC
MF
SS
D
Hypergate neoplastic T cell ID
Contribution
E
PC loadings
F
Metabolism PC1
G
Median metabolic expression
r=0.82
p=6e−05
Non-naive CD4+ T cells
Neoplastic T cells
H
Neoplastic metabolic profile
I
Normal T cells
Neoplastic T cells
CD3
CTLA-4
J
Mean CTLA-4
K
PD−1+
PD−1++
% of population
L
Normal T cells
Neoplastic T cells
Subject
% of PBMCs
M
%PD−1+/++ of PBMCs
N
Neoplastic T cells
Timepoint
O
%HLA−DR+
P
Mean %+
Significant
Q
%PD−1+/++
R
%CD38+
S
%CD56+
T
R
NR
U
TCR sequences
Probability of generation
Neoplastic
Normal

Figure S2: Neoplastic T cell phenotype and response to therapy – related to Figure 2

A) Median expression of differential proteins by non-naïve CD4+ and neoplastic T cells by subject.
B) Predictors included in HSS-LDA models to separate neoplastic and normal CD4+ T cells in equal sampling of HC and leukemic subjects (bottom) and model accuracy (top).
C) Per subject accuracy metrics for manual gating schemes shown in Fig. 2C. Crossbar indicates mean. Wilcoxon signed rank.
D) Marker contribution quantified by Hypergate in model trained to separate neoplastic and normal CD4+ T cells in equal in equal sampling of HC and leukemic subjects.
E) PC loadings of PCA manifold from Fig. 2E
F) PC1 coordinates by cell type; same manifold as Fig. 2E. Crossbar indicates mean. Wilcoxon signed rank.
G) Mean of median expression of metabolic markers for non-naïve $CD4^+$ and neoplastic T cells. Fitting line calculated by linear regression. R- and p-value calculated by Pearson method.
H) PCA coordinates of neoplastic T cells, colored by timepoint; same manifold as Fig. 2E. Ellipses indicate 95th percentile CI assuming a multivariate t-distribution.
I) Expression of equal sampling of normal or neoplastic T cells at BL, colored by density. Numbers indicate percent in gate.
J) Mean CTLA-4 expression in normal and neoplastic T cells. Crossbar indicates mean. Wilcoxon signed rank.
K) % $PD\text{-}1^+$ and $\%PD\text{-}1^{++}$ normal or neoplastic T cells. Crossbar indicates mean. FDR-corrected Wilcoxon signed rank.
L) %PD-1 expression level by normal or neoplastic T cells of total PBMCs.
M) $\%PD\text{-}1^{+/++}$ normal or neoplastic T cells of total PBMCs. Crossbar indicates mean. Wilcoxon signed rank.
N) % neoplastic T cells of PBMCs by timepoint. Crossbar indicates mean. Wilcoxon signed rank.
O) $\%HLA\text{-}DR^+$ neoplastic T cells by timepoint. Crossbar indicates mean. FDR-adjusted Wilcoxon signed rank.
P) Mean %+ of activation markers in neoplastic T cells by timepoint. Red box indicates significance. FDR-corrected Wilcoxon signed rank.
Q) $\%PD\text{-}1^{+/++}$ neoplastic T cells by timepoint. Crossbar indicates mean. Wilcoxon signed rank.
R) $\%CD38^+$ neoplastic T cells by timepoint for subjects with late timepoint data. Crossbar indicates mean. FDR-adjusted Wilcoxon signed rank.
S) $\%CD56^+$ neoplastic T cells by timepoint for subjects with late timepoint data. Crossbar indicates mean. FDR-adjusted Wilcoxon signed rank.
T) % neoplastic T cells of total PBMCs by timepoint for subjects with late timepoint data colored by therapeutic response. Crossbar indicates mean.
U) Probably of generation of TCR sequence for neoplastic TCRs (dots) and total normal repertoires (violin). Distributions were not significantly different (p=0.533, Wilcoxon rank sum).

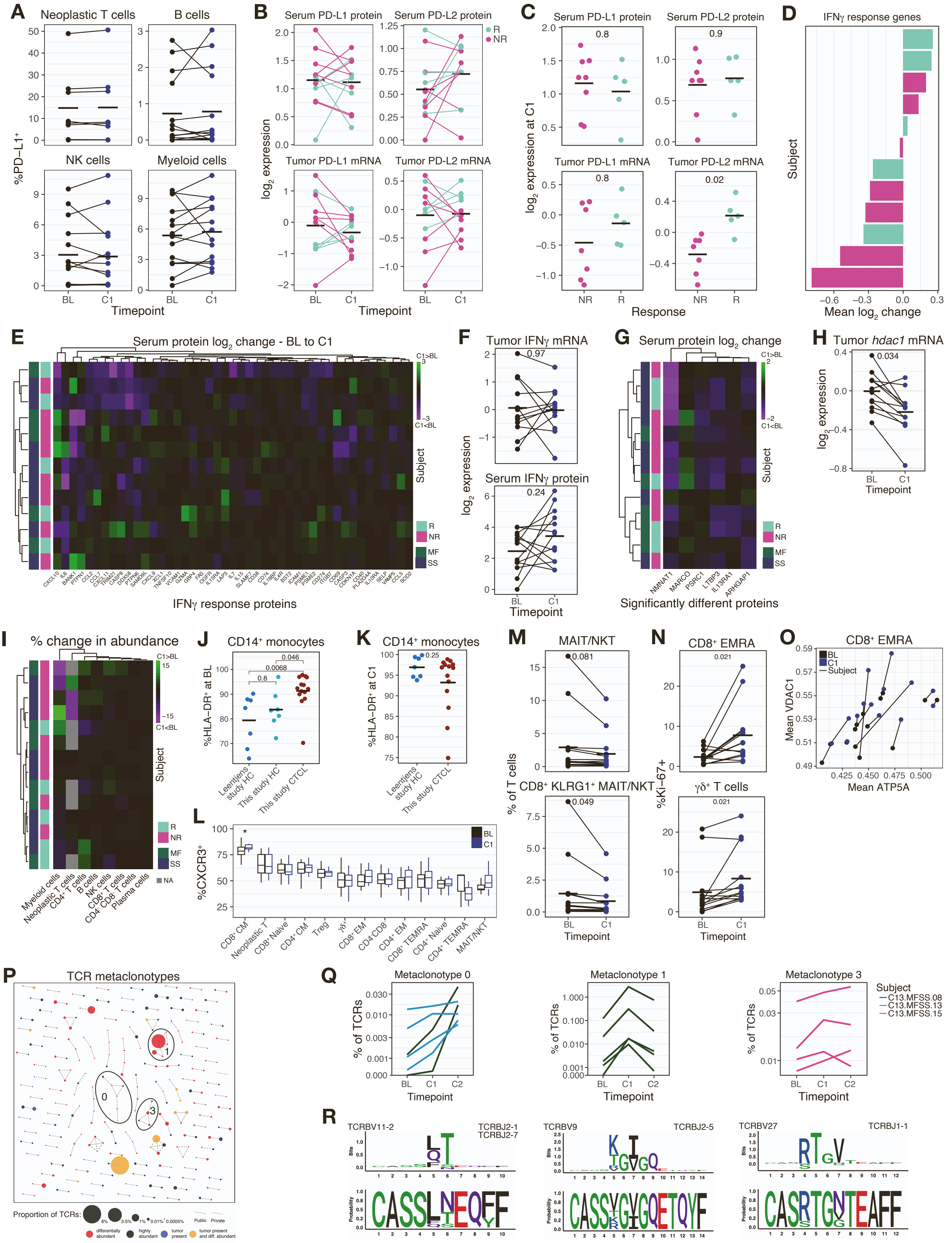
A
Neoplastic T cells
B cells
NK cells
Myeloid cells
%PD–L1+
Timepoint
BL
C1
B
Serum PD-L1 protein
Serum PD-L2 protein
Tumor PD-L1 mRNA
Tumor PD-L2 mRNA
R
NR
log2 expression
Timepoint
C
Serum PD-L1 protein
Serum PD-L2 protein
Tumor PD-L1 mRNA
Tumor PD-L2 mRNA
0.8
0.9
0.8
0.02
log2 expression at C1
Response
NR
R
D
IFNγ response genes
Subject
Mean log2 change
E
Serum protein log2 change - BL to C1
C1>BL
C1<BL
Subject
R
NR
MF
SS
IFNγ response proteins
F
Tumor IFNγ mRNA
0.97
Serum IFNγ protein
0.24
log2 expression
Timepoint
G
Serum protein log2 change
NMNAT1
MARCO
PSRC1
LTBP3
IL13RA1
ARHGAP1
Significantly different proteins
H
Tumor hdac1 mRNA
0.034
log2 expression
Timepoint
I
% change in abundance
Myeloid cells
Neoplastic T cells
CD4+ T cells
B cells
NK cells
CD8+ T cells
CD4-CD8- T cells
Plasma cells
NA
J
CD14+ monocytes
%HLA–DR+ at BL
0.8
0.0068
0.046
Leentjens study HC
This study HC
This study CTCL
K
CD14+ monocytes
%HLA–DR+ at C1
0.25
L
%CXCR3+
CD8+ CM
Neoplastic T
CD8+ Naive
CD4+ CM
Treg
γδ+
CD8+ EM
CD4-CD8-
CD4+ EM
CD8+ TEMRA
CD4+ Naive
CD4+ TEMRA
MAIT/NKT
M
MAIT/NKT
0.081
CD8+ KLRG1+ MAIT/NKT
0.049
% of T cells
Timepoint
N
CD8+ EMRA
0.021
γδ+ T cells
0.021
%Ki–67+
Timepoint
O
CD8+ EMRA
BL
C1
Subject
Mean VDAC1
Mean ATP5A
P
TCR metaclonotypes
Proportion of TCRs:
6%
3.5%
1%
0.01%
0.0005%
Public
Private
differentially abundant
highly abundant
tumor present
tumor present and diff. abundant
Q
Metaclonotype 0
Metaclonotype 1
Metaclonotype 3
% of TCRs
Timepoint
Subject
C13.MFSS.08
C13.MFSS.13
C13.MFSS.15
R
TCRBV11-2
TCRBJ2-1
TCRBJ2-7
TCRBV9
TCRBJ2-5
TCRBV27
TCRBJ1-1
Bits
Probability
CASSLNEQFF
CASSYGVGQETQYF
CASRTGNTEAFF

Figure S3: *In vivo* effects of interferon-gamma – related to Figure 3

A) %PD-L1^{+} cells by cell type and timepoint. Crossbar indicates mean. Differences across timepoints were not significant (q>0.1). FDR-corrected Wilcoxon signed rank.
B) Log_2 expression of serum proteins and tumor mRNA by timepoint and colored by response. Crossbar indicates mean. Differences across timepoints were not significant (q>0.1). FDR-corrected Wilcoxon signed rank.
C) Log_2 expression of serum proteinsand tumor mRNA at C1 by therapeutic response. Crossbar indicates mean. FDR-corrected Wilcoxon rank sum.
D) Mean log_2 change in expression of IFNg response genes in tumor mRNA between BL and C1, by subject, colored by response.
E) Log_2 change in expression of IFNg response genes in serum proteins between BL and C1, by subject.
F) Log_2 expression of serum protein and tumor mRNA by timepoint. Crossbar indicates mean. Wilcoxon signed rank.
G) Log_2 change in expression of serum proteins between BL and C1, by subject. All proteins were significantly differentially expressed (p<0.05 & |effect size|>1). Wilcoxon signed rank.
H) Log_2 expression of tumor mRNA by timepoint. Crossbar indicates mean. Wilcoxon signed rank; |effect size|>1.
I) Difference in cell type abundance between BL and C1. Differences across timepoints were not significant (q>0.1). FDR-corrected Wilcoxon signed rank.
J) %HLA-DR^{+} of CD14^{+} monocytes at BL by cohort and disease status. Crossbar indicates mean. FDR-corrected Wilcoxon rank sum.
K) %HLA-DR^{+} of CD14^{+} monocytes at C1 by cohort and disease status. Crossbar indicates mean. Wilcoxon rank sum.
L) %CXCR3^{+} of T cell populations, separated by timepoint. * indicates q<0.1. FDR-corrected Wilcoxon signed rank. Boxplots represent interquartile range (IQR); horizontal line indicates median; whiskers extend to the farthest data point within 1.5 × IQR.
M) % T cell populations of non-neoplastic T cells by timepoint. Crossbar indicates mean. FDR-corrected Wilcoxon signed rank.
N) % Ki-67^{+} of T cell populations by timepoint. Crossbar indicates mean. FDR-corrected Wilcoxon signed rank.
O) Mean expression of metabolic markers by CD8^{+} EMRA T cells across timepoints. Both markers were significantly differentially expressed between timepoints (q<0.1). FDR-corrected Wilcoxon signed rank.
P) TCR clones, colored by indicated criteria, organized into metaclones.
Q) % of TCRs by clone, colored by subject, separated by metaclone.
R) V and J gene usage and CDR3 sequence motifs of the metaclones indicated in (Q). The top sequence logo shows the information relative to amino acids found in a background set of randomly sampled CDR3 sequences with the same V and J genes.

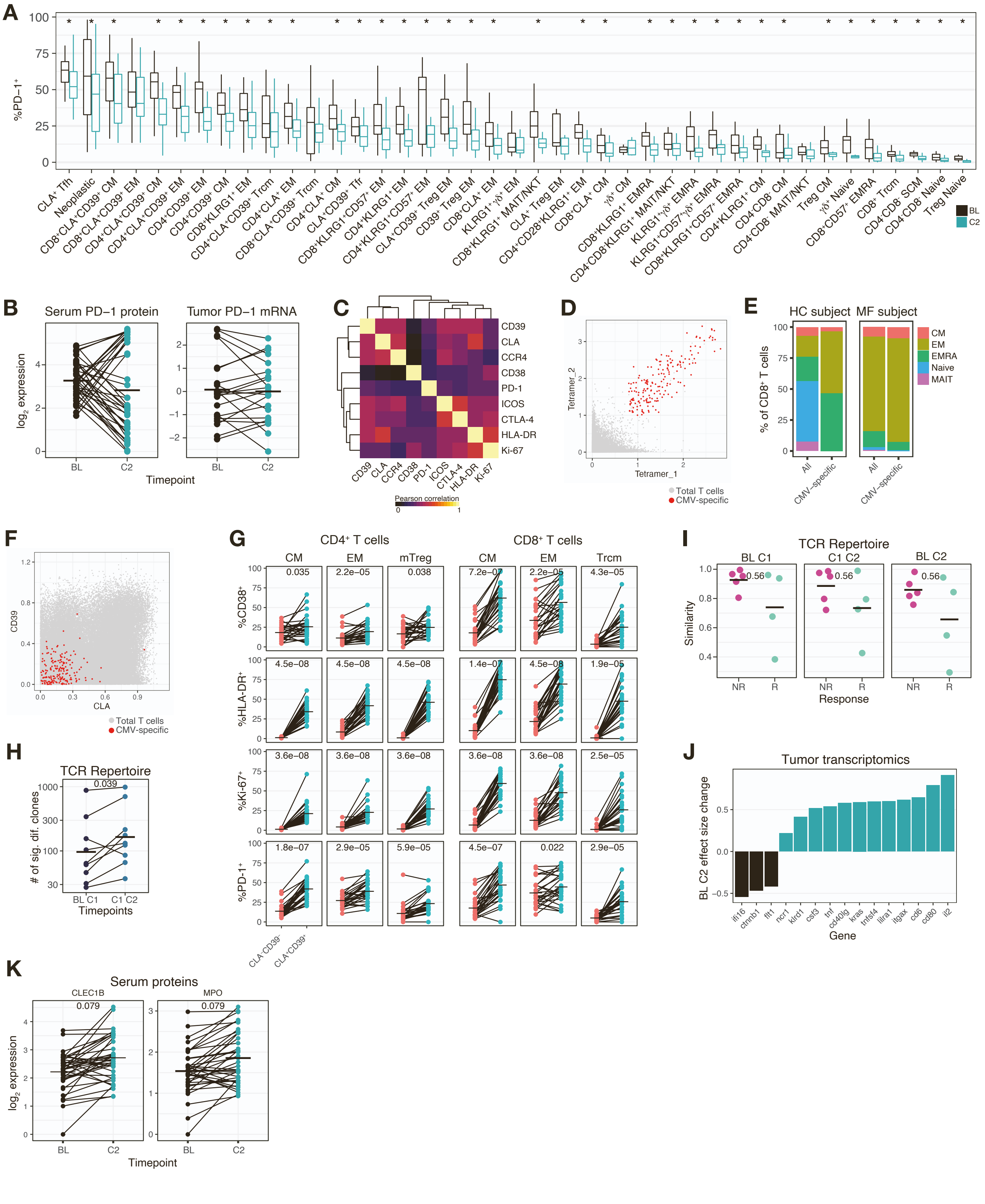
A
%PD-1+
BL
C2
B
Serum PD−1 protein
Tumor PD−1 mRNA
Timepoint
C
Pearson correlation
D
Tetramer_1
Tetramer_2
Total T cells
CMV-specific
E
HC subject
MF subject
F
CLA
CD39
G
CD4+ T cells
CD8+ T cells
H
TCR Repertoire
Timepoints
I
TCR Repertoire
Response
J
Tumor transcriptomics
Gene
K
Serum proteins
CLEC1B
MPO
Timepoint

Figure S4: *In vivo* effects of anti-PD-1 – related to Figure 4

A) %PD-1^{+} T cell populations, separated by timepoint. * indicates $q<0.1$. FDR-corrected Wilcoxon signed rank. Boxplots represent IQR; horizontal line indicates median; whiskers extend to the farthest data point within 1.5 × IQR.

B) Log_2 expression of serum proteins and tumor mRNA by timepoint. Crossbar indicates mean. Differences across timepoints were not significant ($q>0.1$). FDR-corrected Wilcoxon signed rank.

C) Pearson correlation between proteins expressed on non-neoplastic T cells at C2, post-anti-PD-1 treatment.

D) Exemplative staining of T cells from an equal sampling of non-neoplastic T cells pre- and post-anti-PD-1 treatment.

E) Composition of T cell subsets comprising total $CD8^{+}$ T cells and CMV-specific $CD8^{+}$ T cells in two subjects.

F) Exemplative staining of T cells from an equal sampling of non-neoplastic T cells pre- and post-anti-PD-1 treatment.

G) $\%^{+}$ activation markers across T cell populations by timepoint. FDR-corrected Wilcoxon signed rank.

H) The number of significantly differentially abundant clones ($q<0.1$) between indicated timepoints as calculated by FDR-corrected Fisher's exact test. P-value calculated from Wilcoxon signed rank test.

I) One minus the Horn-Morista index of the non-neoplastic TCR repertoire similarity of CTCL subjects at indicated timepoints by response. Crossbar indicates mean. FDR-corrected Wilcoxon rank sum.

J) Effect size difference for genes not significantly differentially expressed after multiple hypothesis correction ($p<0.05$; $q>0.1$) between BL and C2. Uncorrected/FDR-corrected Wilcoxon signed rank.

K) Log_2 expression of serum proteins significantly differentially expressed ($q<0.1$) between BL and C2. Crossbar indicates mean. FDR-corrected Wilcoxon signed rank.

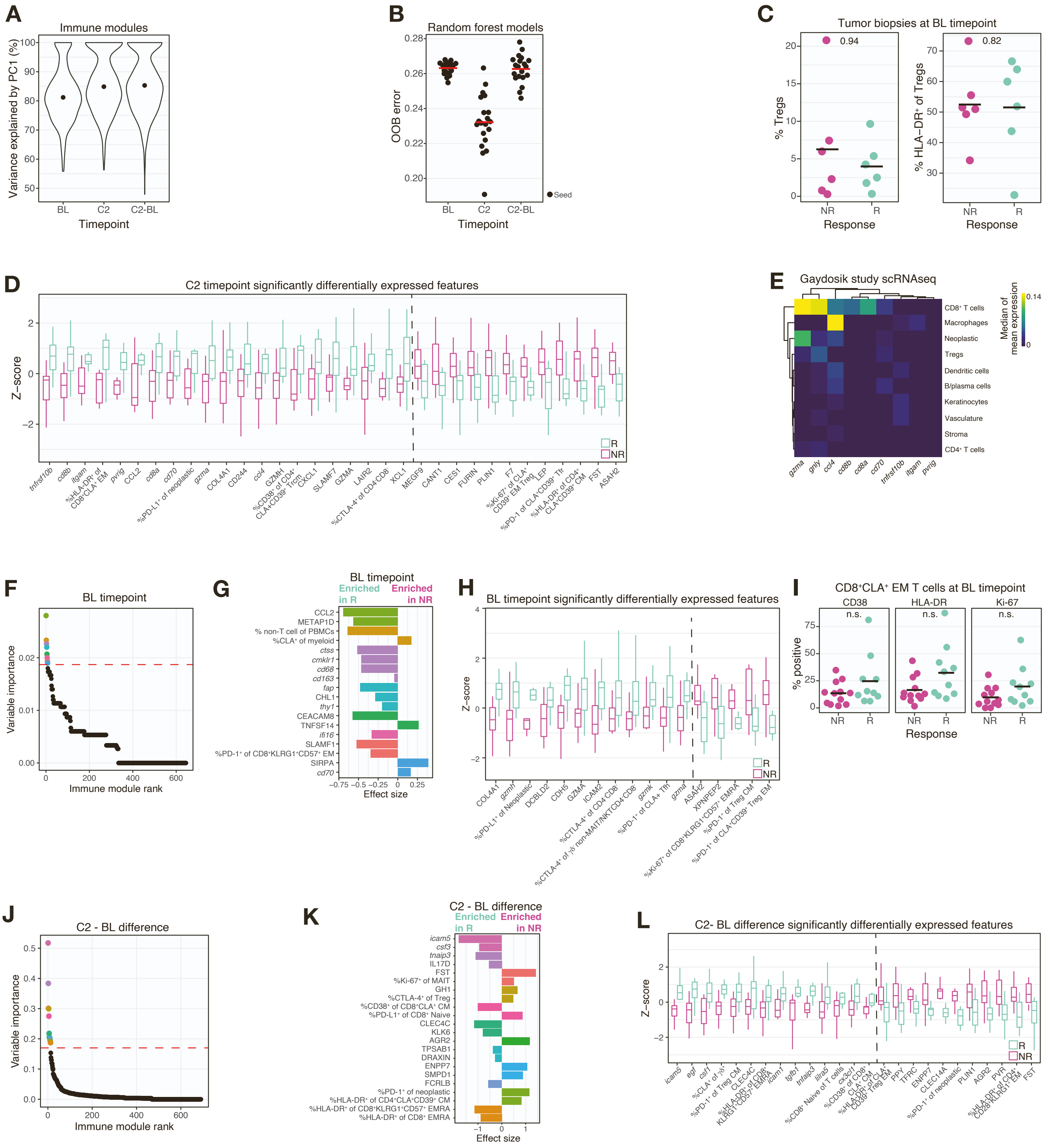

A
Immune modules
Variance explained by PC1 (%)
100
90
80
70
60
50
BL
C2
C2-BL
Timepoint
B
Random forest models
OOB error
0.28
0.26
0.24
0.22
0.20
Seed
BL
C2
C2-BL
Timepoint
C
Tumor biopsies at BL timepoint
% Tregs
0.94
% HLA−DR+ of Tregs
0.82
NR
R
Response
D
C2 timepoint significantly differentially expressed features
Z−score
R
NR
tnfrsf10b
cd8b
itgam
%HLA-DR+ of CD8+CLA+ EM
pvrig
CCL2
cd8a
cd70
%PD-L1+ of neoplastic
gzma
COL4A1
CD244
ccl4
GZMH
%CD38+ of CD4+ CLA+CD39+ Tcm
CXCL1
SLAMF7
GZMA
LAIR2
%CTLA-4+ of CD4 CD8
XCL1
MEGF9
CANT1
CES1
FURIN
PLIN1
F7
%Ki-67+ of CLA+ CD39+ EM Treg
LEP
%PD-1 of CLA+CD39+ Tfr
%HLA-DR+ of CD4+ CLA+CD39+ CM
FST
ASAH2
E
Gaydosik study scRNAseq
Median of mean expression
0.14
0
CD8+ T cells
Macrophages
Neoplastic
Tregs
Dendritic cells
B/plasma cells
Keratinocytes
Vasculature
Stroma
CD4+ T cells
gzma
gnly
ccl4
cd8b
cd8a
cd70
tnfrsf10b
itgam
pvrig
F
BL timepoint
Variable importance
Immune module rank
G
BL timepoint
Enriched in R
Enriched in NR
CCL2
METAP1D
% non-T cell of PBMCs
%CLA+ of myeloid
ctss
cmklr1
cd68
cd163
fap
CHL1
thy1
CEACAM8
TNFSF14
ifi16
SLAMF1
%PD-1+ of CD8+KLRG1+CD57+ EM
SIRPA
cd70
Effect size
H
BL timepoint significantly differentially expressed features
Z−score
R
NR
COL4A1
gzmh
%PD-L1+ of Neoplastic
DCBLD2
CDH5
GZMA
ICAM2
%CTLA-4+ of CD4 CD8
%CTLA-4+ of γδ non-MAIT/NKT CD4 CD8
gzmk
%PD-1+ of CLA+ Tfh
gzma
ASAH2
XPNPEP2
%Ki-67+ of CD8+KLRG1+CD57+ EMRA
%PD-1+ of Treg CM
%PD-1+ of CLA+CD39+ Treg EM
I
CD8+CLA+ EM T cells at BL timepoint
CD38
HLA-DR
Ki-67
n.s.
% positive
NR
R
Response
J
C2 - BL difference
Variable importance
Immune module rank
K
C2 - BL difference
Enriched in R
Enriched in NR
icam5
csf3
tnaip3
IL17D
FST
%Ki-67+ of MAIT
GH1
%CTLA-4+ of Treg
%CD38+ of CD8+CLA+ CM
%PD-L1+ of CD8+ Naive
CLEC4C
KLK6
AGR2
TPSAB1
DRAXIN
ENPP7
SMPD1
FCRLB
%PD-1+ of neoplastic
%HLA-DR+ of CD4+CLA+CD39+ CM
%HLA-DR+ of CD8+KLRG1+CD57+ EMRA
%HLA-DR+ of CD8+ EMRA
Effect size
L
C2- BL difference significantly differentially expressed features
Z−score
R
NR
icam5
egl
csf1
%CLA+ of γδ
%PD-1+ of Treg CM
CLEC4C
%HLA-DR+ of CD8+ KLRG1+CD57+ EMRA
icam1
tgfb1
tnfaip3
lilra5
%CD8+ Naive of T cells
cx3cl1
%CD38+ of CD8+ CLA+ CM
%HLA-DR+ of CLA+ CD39+ Treg EM
PPY
TFRC
ENPP7
CLEC14A
%PD-1+ of neoplastic
PLIN1
AGR2
PVR
%HLA-DR+ of CD4+ CD28+KLRG1+ EM
FST

Figure S5: Identification of immune features stratifying responders and non-responders – related to Figure 5

A) % variance explained by PC1 within each immune module by timepoint. Dots indicate median.
B) Out-of-bag errors across different seeds of the random forest models generated to predict therapeutic response using immune modules by timepoint. Crossbar indicates mean. Lower error indicates improved accuracy.
C) % Tregs of T cells and %Ki-67$^+$ Tregs at BL by response. Crossbar indicates mean. FDR-corrected Wilcoxon rank sum.
D) Z-scores of significantly differentially expressed features ($p<0.05$ & $|effect|>1$) stratifying therapeutic response at C2. Uncorrected Wilcoxon rank sum. Boxplots represent IQR; horizontal line indicates median; whiskers extend to the farthest data point within 1.5 × IQR. Dashed line separates features enriched in responders and features enriched in non-responders.
E) Median of mean expression of genes identified in (D) by cell population.
F) Gini index of immune module predictors separating response and non-response at the BL timepoint. Dashed red line indicates threshold set for immune modules shown in (G). Colors correspond to immune modules in (G).
G) Effect size of underlying features comprising immune modules selected in (F). Bar color denotes immune module membership. Features are ordered by Gini index.
H) Z-scores of significantly differentially expressed features ($p<0.05$ & $|effect|>1$) stratifying therapeutic response at BL. Wilcoxon rank sum. Boxplots represent IQR; horizontal line indicates median; whiskers extend to the farthest data point within 1.5 × IQR. Dashed line separates features enriched in responders and features enriched in non-responders.
I) %$^+$ of activation markers by CD8$^+$CLA$^+$ EM T cells at BL by response. Crossbar indicates mean. No comparison was statistically significant ($q>0.1$). FDR-corrected Wilcoxon rank sum test.
J) Gini index of immune module predictors separating response and non-response in the C2-BL timepoint. Dashed red line indicates threshold set for immune modules shown in K. Colors correspond to immune modules in (K).
K) Effect size of underlying features comprising immune modules selected in (J). Bar color denotes immune module membership. Features are ordered by Gini index.
L) Z-scores of significantly differentially expressed features ($p<0.05$ & $|effect|>1$) stratifying therapeutic response at C2-BL difference. Wilcoxon rank sum. Boxplots represent IQR; horizontal line indicates median; whiskers extend to the farthest data point within 1.5 × IQR. Dashed line separates features enriched in responders and features enriched in non-responders.

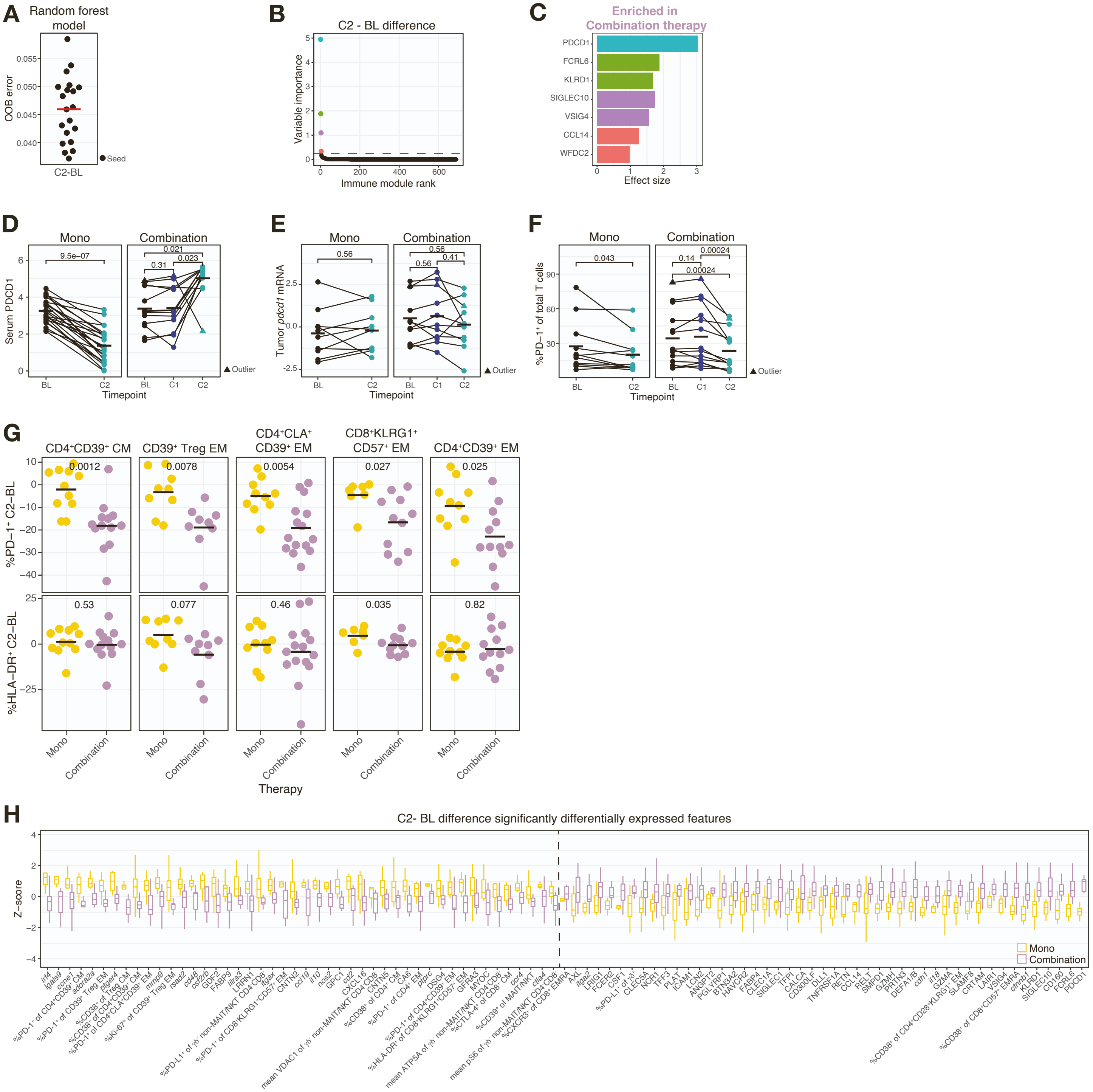
A
Random forest model
OOB error
Seed
C2-BL
B
C2 - BL difference
Variable importance
Immune module rank
C
Enriched in Combination therapy
PDCD1
FCRL6
KLRD1
SIGLEC10
VSIG4
CCL14
WFDC2
Effect size
D
Mono
Combination
Serum PDCD1
Timepoint
Outlier
E
Tumor pdcd1 mRNA
F
%PD−1+ of total T cells
G
CD4+CD39+ CM
CD39+ Treg EM
CD4+CLA+ CD39+ EM
CD8+KLRG1+ CD57+ EM
CD4+CD39+ EM
%PD−1+ C2−BL
%HLA−DR+ C2−BL
Therapy
H
C2- BL difference significantly differentially expressed features
Z-score
Mono
Combination

Figure S6: Mono and combination therapy induce differential abundance of PD-1 – related to Figure 5

A) Out-of-bag errors across different seeds of the random forest models generated to predict therapy regimen using immune modules at the C2-BL difference. Crossbar indicates mean. Lower error indicates improved accuracy.

B) Gini index of immune module predictors separating mono and combination therapy in the C2-BL timepoint. Dashed red line indicates threshold set for immune modules shown in (C). Colors correspond to immune modules in (C).

C) Effect size of underlying features comprising immune modules selected in (B). Bar color denotes immune module membership. Features are ordered by Gini index.

D) Log_2 expression of serum PD-1 by clinical trial and timepoint. Crossbar indicates mean. FDR-corrected Wilcoxon signed rank.

E) Log_2 expression of tumor *pdcd1* mRNA by clinical trial and timepoint. Crossbar indicates mean. FDR-corrected Wilcoxon signed rank.

F) %PD-1^+ of total T cells by clinical trial and timepoint. Crossbar indicates mean. FDR-corrected Wilcoxon signed rank.

G) Difference in %PD-1^+ (top row) or %HLA-DR^+ (bottom row) between C2 and BL in T cell populations by clinical trial. Crossbar indicates mean. FDR-corrected Wilcoxon rank sum.

H) Z-scores of significantly differentially expressed features ($p<0.05$ & $|effect|>1$) stratifying response to mono and combination therapy at C2-BL difference. Wilcoxon rank sum. Boxplots represent IQR; horizontal line indicates median; whiskers extend to the farthest data point within 1.5 × IQR. Dashed line separates features enriched in monotherapy and features enriched in combination therapy.

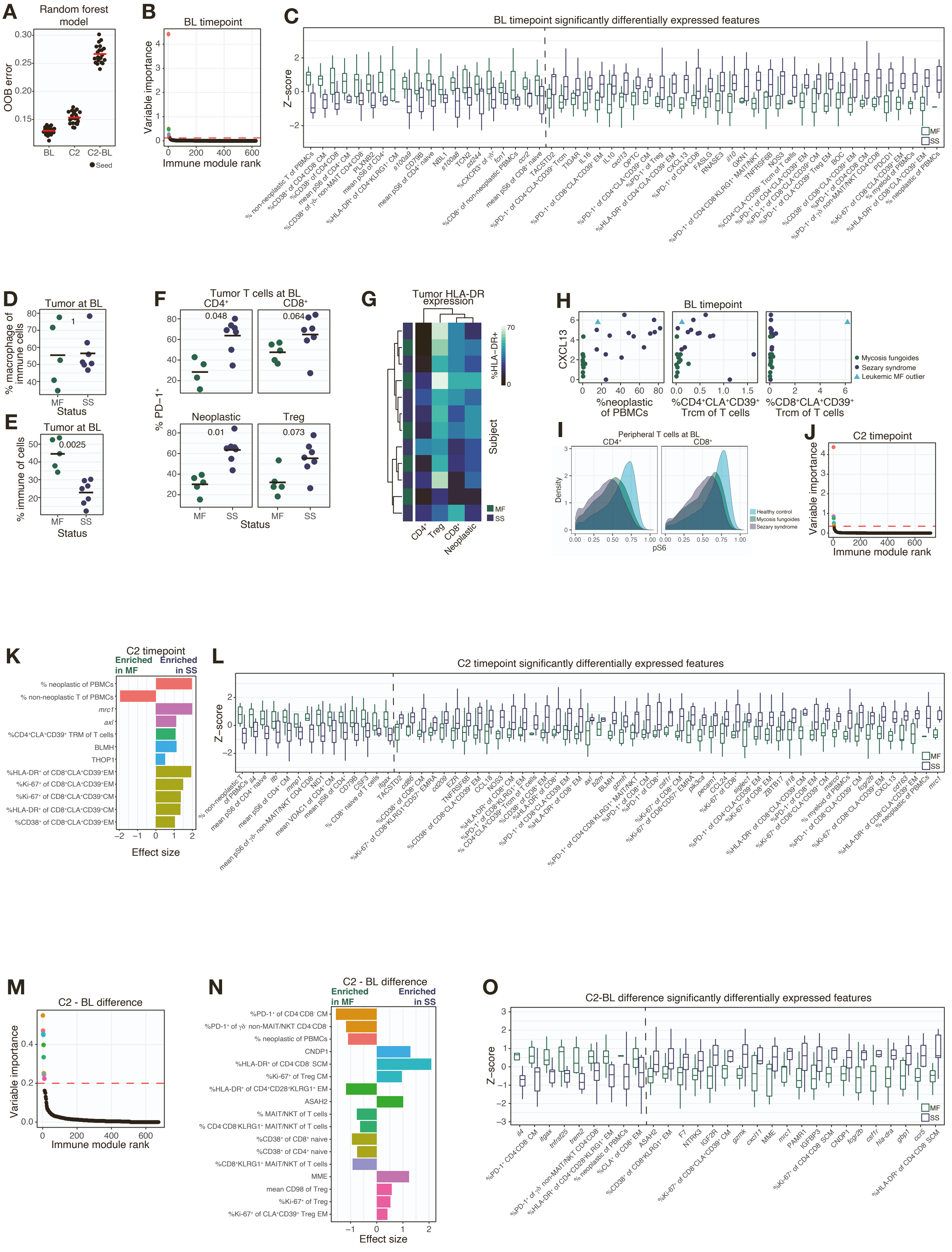
A
Random forest model
OOB error
BL
C2
C2-BL
Seed
B
BL timepoint
Variable importance
Immune module rank
C
BL timepoint significantly differentially expressed features
Z-score
MF
SS
D
Tumor at BL
% macrophage of immune cells
MF
SS
Status
E
Tumor at BL
% immune of cells
0.0025
Status
F
Tumor T cells at BL
CD4⁺
CD8⁺
0.048
0.064
Neoplastic
Treg
0.01
0.073
% PD−1⁺
Status
G
Tumor HLA-DR expression
%HLA-DR+
Subject
CD4⁺
Treg
CD8⁺
Neoplastic
MF
SS
H
BL timepoint
CXCL13
%neoplastic of PBMCs
%CD4⁺CLA⁺CD39⁺ Trcm of T cells
%CD8⁺CLA⁺CD39⁺ Trcm of T cells
Mycosis fungoides
Sezary syndrome
Leukemic MF outlier
I
Peripheral T cells at BL
CD4⁺
CD8⁺
Density
pS6
Healthy control
Mycosis fungoides
Sezary syndrome
J
C2 timepoint
Variable importance
Immune module rank
K
C2 timepoint
Enriched in MF
Enriched in SS
% neoplastic of PBMCs
% non-neoplastic T of PBMCs
mrc1
axl
%CD4⁺CLA⁺CD39⁺ TRM of T cells
BLMH
THOP1
%HLA-DR⁺ of CD8⁺CLA⁺CD39⁺EM
%Ki-67⁺ of CD8⁺CLA⁺CD39⁺EM
%Ki-67⁺ of CD8⁺CLA⁺CD39⁺CM
%HLA-DR⁺ of CD8⁺CLA⁺CD39⁺CM
%CD38⁺ of CD8⁺CLA⁺CD39⁺EM
Effect size
L
C2 timepoint significantly differentially expressed features
Z-score
MF
SS
M
C2 - BL difference
Variable importance
Immune module rank
N
C2 - BL difference
Enriched in MF
Enriched in SS
%PD-1⁺ of CD4⁻CD8⁻ CM
%PD-1⁺ of γδ⁻ non-MAIT/NKT CD4⁻CD8⁻
% neoplastic of PBMCs
CNDP1
%HLA-DR⁺ of CD4⁻CD8⁻ SCM
%Ki-67⁺ of Treg CM
%HLA-DR⁺ of CD4⁺CD28⁺KLRG1⁺ EM
ASAH2
% MAIT/NKT of T cells
% CD4⁻CD8⁻KLRG1⁺ MAIT/NKT of T cells
%CD38⁺ of CD8⁺ naive
%CD38⁺ of CD4⁺ naive
%CD8⁺KLRG1⁺ MAIT/NKT of T cells
MME
mean CD98 of Treg
%Ki-67⁺ of Treg
%Ki-67⁺ of CLA⁺CD39⁺ Treg EM
Effect size
O
C2-BL difference significantly differentially expressed features
Z-score
MF
SS

Figure S7: Identification of immune features stratifying MF and SS – related to Figure 6

A) Out-of-bag errors across different seeds of the random forest models generated to predict disease status using immune modules, separated by timepoint. Crossbar indicates mean. Lower error indicates improved accuracy.

B) Gini index of immune module predictors separating MF and SS at the BL timepoint. Dashed red line indicates threshold set for immune modules shown in Fig. 6A. Colors correspond to immune modules in Fig. 6A.

C) Z-scores of significantly differentially expressed features ($p<0.05$ & $|effect|>1$) stratifying MF and SS subjects at BL. Wilcoxon rank sum. Boxplots represent IQR; horizontal line indicates median; whiskers extend to the farthest data point within 1.5 × IQR. Dashed line separates features enriched in MF and features enriched in SS.

D) % macrophages of immune cells in tumor samples at BL. Crossbar indicates mean. Wilcoxon rank sum.

E) % immune cells of total cells in tumor samples at BL. Crossbar indicates mean. Wilcoxon rank sum.

F) %PD-1^{+} of indicated T cell populations in tumor samples at BL. Crossbar indicates mean. FDR-corrected Wilcoxon rank sum.

G) %HLA-DR^{+} of indicated tumor-present T cell populations at BL by subject.

H) CXCL13 serum protein level and abundance of indicated cell populations at BL.

I) Expression of pS6 for indicated T cell populations drawn from an equal sampling of cells from HC, MF, and SS subjects at BL.

J) Gini index of immune module predictors separating MF and SS at the BL timepoint. Dashed red line indicates threshold set for immune modules shown in (K). Colors correspond to immune modules in (K).

K) Effect size of underlying features comprising immune modules selected by model differentiating MF and SS subjects at C2. Bar color denotes immune module membership as in (J). Features are ordered by Gini index.

L) Z-scores of significantly differentially expressed features ($p<0.05$ & $|effect|>1$) stratifying MF and SS at C2. Wilcoxon rank sum. Boxplots represent IQR; horizontal line indicates median; whiskers extend to the farthest data point within 1.5 × IQR. Dashed line separates features enriched in MF and features enriched in SS.

M) Gini index of immune module predictors separating MF and SS at C2-BL difference. Dashed red line indicates threshold set for immune modules shown in (N). Colors correspond to immune modules in (N).

N) Effect size of underlying features comprising immune modules selected by model differentiating MF and SS subjects at C2-BL difference. Bar color denotes immune module membership as in (M). Features are ordered by Gini index.

O) Z-scores of significantly differentially expressed features ($p<0.05$ & $|effect|>1$) stratifying MF and SS subjects at C2-BL difference. Wilcoxon rank sum. Boxplots represent IQR; horizontal line indicates median; whiskers extend to the farthest data point within 1.5 × IQR. Dashed line separates features enriched in MF and features enriched in SS.

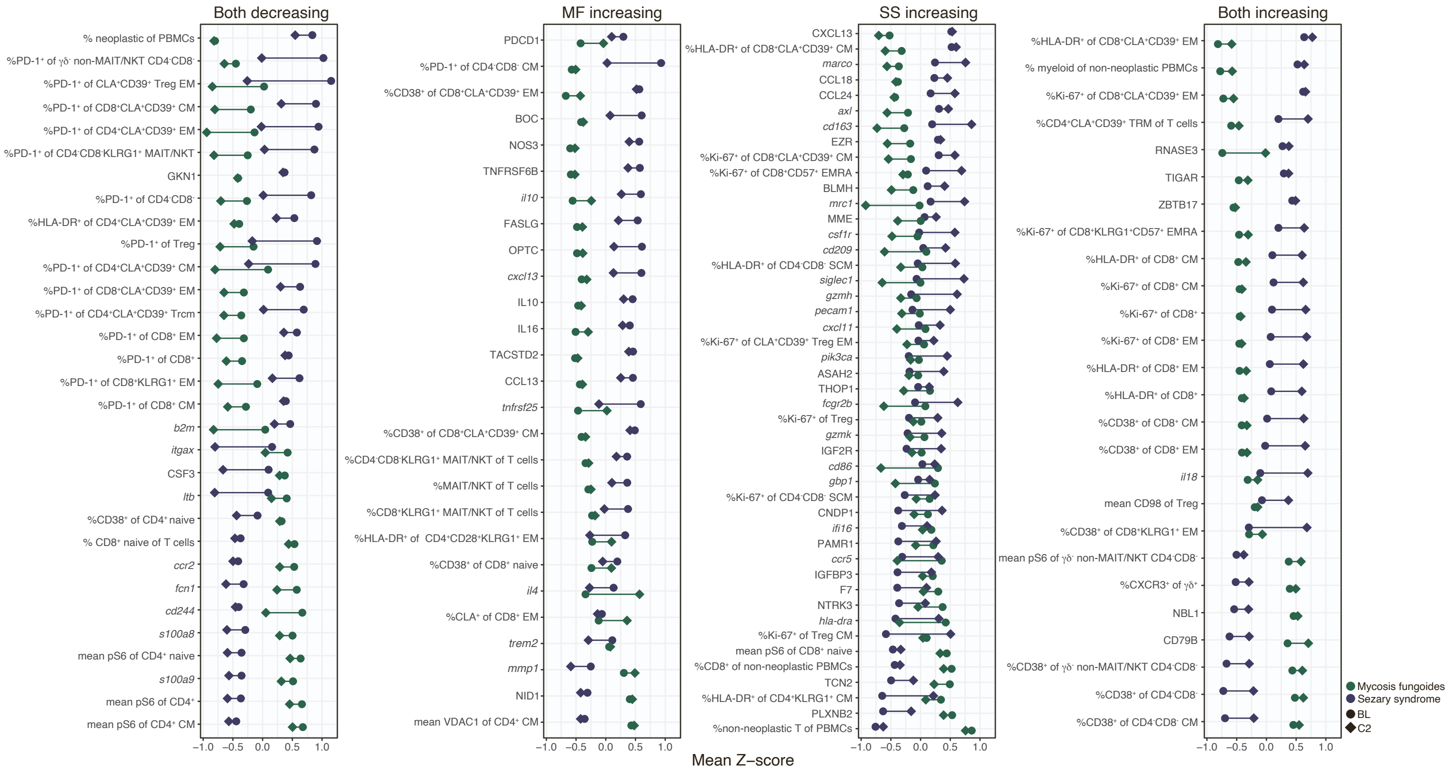

Both decreasing
% neoplastic of PBMCs
%PD-1+ of γδ- non-MAIT/NKT CD4-CD8-
%PD-1+ of CLA+CD39+ Treg EM
%PD-1+ of CD8+CLA+CD39+ CM
%PD-1+ of CD4+CLA+CD39+ EM
%PD-1+ of CD4-CD8-KLRG1+ MAIT/NKT
GKN1
%PD-1+ of CD4-CD8-
%HLA-DR+ of CD4+CLA+CD39+ EM
%PD-1+ of Treg
%PD-1+ of CD4+CLA+CD39+ CM
%PD-1+ of CD8+CLA+CD39+ EM
%PD-1+ of CD4+CLA+CD39+ Trcm
%PD-1+ of CD8+ EM
%PD-1+ of CD8+
%PD-1+ of CD8+KLRG1+ EM
%PD-1+ of CD8+ CM
b2m
itgax
CSF3
ltb
%CD38+ of CD4+ naive
% CD8+ naive of T cells
ccr2
fcn1
cd244
s100a8
mean pS6 of CD4+ naive
s100a9
mean pS6 of CD4+
mean pS6 of CD4+ CM
MF increasing
PDCD1
%PD-1+ of CD4-CD8- CM
%CD38+ of CD8+CLA+CD39+ EM
BOC
NOS3
TNFRSF6B
il10
FASLG
OPTC
cxcl13
IL10
IL16
TACSTD2
CCL13
tnfrsf25
%CD38+ of CD8+CLA+CD39+ CM
%CD4-CD8-KLRG1+ MAIT/NKT of T cells
%MAIT/NKT of T cells
%CD8+KLRG1+ MAIT/NKT of T cells
%HLA-DR+ of CD4+CD28+KLRG1+ EM
%CD38+ of CD8+ naive
il4
%CLA+ of CD8+ EM
trem2
mmp1
NID1
mean VDAC1 of CD4+ CM
SS increasing
CXCL13
%HLA-DR+ of CD8+CLA+CD39+ CM
marco
CCL18
CCL24
axl
cd163
EZR
%Ki-67+ of CD8+CLA+CD39+ CM
%Ki-67+ of CD8+CD57+ EMRA
BLMH
mrc1
MME
csf1r
cd209
%HLA-DR+ of CD4-CD8- SCM
siglec1
gzmh
pecam1
cxcl11
%Ki-67+ of CLA+CD39+ Treg EM
pik3ca
ASAH2
THOP1
fcgr2b
%Ki-67+ of Treg
gzmk
IGF2R
cd86
gbp1
%Ki-67+ of CD4-CD8- SCM
CNDP1
ifi16
PAMR1
ccr5
IGFBP3
F7
NTRK3
hla-dra
%Ki-67+ of Treg CM
mean pS6 of CD8+ naive
%CD8+ of non-neoplastic PBMCs
TCN2
%HLA-DR+ of CD4+KLRG1+ CM
PLXNB2
%non-neoplastic T of PBMCs
Both increasing
%HLA-DR+ of CD8+CLA+CD39+ EM
% myeloid of non-neoplastic PBMCs
%Ki-67+ of CD8+CLA+CD39+ EM
%CD4+CLA+CD39+ TRM of T cells
RNASE3
TIGAR
ZBTB17
%Ki-67+ of CD8+KLRG1+CD57+ EMRA
%HLA-DR+ of CD8+ CM
%Ki-67+ of CD8+ CM
%Ki-67+ of CD8+
%Ki-67+ of CD8+ EM
%HLA-DR+ of CD8+ EM
%HLA-DR+ of CD8+
%CD38+ of CD8+ CM
%CD38+ of CD8+ EM
il18
mean CD98 of Treg
%CD38+ of CD8+KLRG1+ EM
mean pS6 of γδ- non-MAIT/NKT CD4-CD8-
%CXCR3+ of γδ+
NBL1
CD79B
%CD38+ of γδ- non-MAIT/NKT CD4-CD8-
%CD38+ of CD4-CD8-
%CD38+ of CD4-CD8- CM
−1.0 −0.5 0.0 0.5 1.0
Mean Z−score
Mycosis fungoides
Sezary syndrome
BL
C2

Figure S8: Kinetics of immune features stratifying MF and SS – related to Figure 6

Mean Z-scores of differential features identified at BL, C2 or the change from BL to C2, stratified by status (color), timepoint (shape), and feature kinetics (panels). Facets are ordered by BL effect size difference.

Table S1: Cohort demographics – related to Figure 1

subject	age	gender	status	response	study
C10.10	76	M	MF	PR	10
C10.11	61	M	MF	PR	10
C10.15	66	M	SS	SD	10
C10.21	52	M	SS	SD	10
C10.19	72	F	SS	PD	10
C10.08	85	F	SS	SD	10
C10.23	59	M	SS	SD	10
C10.24	66	M	MF	PR	10
C10.07	72	F	SS	CR	10
C10.03	62	M	MF	SD	10
C10.04	75	M	MF	PR	10
C10.13	70	M	SS	SD	10
C10.22	82	M	SS	SD	10
C10.05	47	M	MF	SD	10
C10.09	47	F	MF	PD	10
C10.16	73	M	SS	SD	10
C10.17	44	F	SS	CR	10
C10.12	72	M	SS	PR	10
C10.18	64	M	MF	PD	10
C10.06	67	M	SS	PD	10
C10.14	69	M	SS	PD	10
C10.02	67	M	SS	PD	10
C10.01	63	M	MF	PR	10
C10.20	78	F	SS	PR	10
C13.MFSS.08	72	F	SS	PR	13
C13.MFSS.10	53	M	MF	PR	13
C13.MFSS.13	43	F	MF	SD	13
C13.MFSS.07	55	M	MF	PD	13
C13.MFSS.14	71	F	MF	PD	13
C13.MFSS.15	57	M	MF	PR	13
C13.MFSS.16	70	M	SS	SD	13
C13.MFSS.02	62	F	SS	SD	13
C13.MFSS.05	74	M	SS	SD	13
C13.MFSS.09	56	M	SS	SD	13

C13.MFSS.12	56	M	MF	PR	13
C13.MFSS.01	63	F	SS	CR	13
C13.MFSS.03	73	M	SS	PD	13
C13.MFSS.04	76	M	MF	CR	13
C13.MFSS.06	56	M	SS	SD	13
HC.02	23	F	HC		
HC.17	47	M	HC		
HC.20	37	M	HC		
HC.23	39	F	HC		
HC.52	26	F	HC		
HC.71	32	F	HC		
HC.81	40	M	HC		

Table S3: Mass cytometry panel – related to Figure 1

Mass	Element	Marker	Localization	Clone	Company	Cat	Stain (ug/test)
89	Y	Barcode_1	Pre-stain	HI30	Standard Bio-tools	3089003B	0.2
103	Rh	DNA	Intracellular	-	Standard Bio-tools	201103A	NA
106	Cd	Barcode_2	Pre-stain	HI30	Standard Bio-tools	3106001B	0.2
110	Cd	Barcode_3	Pre-stain	HI30	Standard Bio-tools	3110001B	0.2
111	Cd	Barcode_4	Pre-stain	HI30	Standard Bio-tools	3111001B	0.2
112	Cd	CD8	Surface	RPA-T8	BioLegend	301002	0.05
113	Cd	CD14	Surface	M5E2	BioLegend	301802	0.8
114	Cd	Barcode_5	Pre-stain	HI30	Standard Bio-tools	3114001B	0.2
115	In	CD57	Surface	HNK-1	BioLegend	359602	0.4
116	Cd	Barcode_6	Surface	HI30	Standard Bio-tools	3116001B	0.2
140	Ce	CD56	Surface	REA196	Miltenyi Biotec	130-108-016	0.025
141	Pr	CLA	Surface	HECA-452	BioLegend	321302	0.2
142	Nd	HLA-DR	Surface	L243	BioLegend	307602	0.8
143	Nd	CXCR5	Surface	J252D4	BioLegend	356902	0.4
144	Nd	CD4	Surface	OKT4	BioLegend	317402	0.8
145	Nd	CD45RA	Surface	HI100	BioLegend	304102	0.05
146	Nd	CD103	Surface	Ber-ACT8	BioLegend	350202	0.2
147	Sm	ICOS	Surface	C398.4A	BioLegend	313502	0.2
148	Nd	CD45RO	Surface	UCHL1	BioLegend	304202	0.8

149	Sm	CCR4	Surface	L291H4	BioLegend	359402	0.2
150	Nd	CD38	Surface	HIT2	BioLegend	303502	0.4
151	Eu	CD127	Surface	QA18A44	BioLegend	375702	0.8
152	Sm	IgG4	Surface	HP6025	Southern Biotech	9200-01	0.1
153	Eu	CD161	Surface	W18070C	BioLegend	307502	0.8
154	Sm	CD26	Surface	BA5b	BioLegend	302702	0.1
155	Gd	Ki-67	Intracellular	Ki-67	BioLegend	350502	0.05
156	Gd	TCRgd	Surface	B1	BioLegend	331202	0.8
157	Gd	CXCR3	Surface	G025H7	BioLegend	353702	0.8
158	Gd	CD27	Surface	M-T271	BioLegend	356401	0.4
159	Tb	VDAC1	Intracellular	20B12AF2	Abcam	ab14734	0.1
160	Gd	CD95	Surface	EOS9.1	BioLegend	305702	0.4
161	Dy	Tetramer	Pre-stain	-		-	0.1
162	Dy	CD25	Surface	BC96	BioLegend	302602	0.4
163	Dy	Tetramer	Pre-stain	-		-	0.1
164	Dy	PD-L1	Surface	29E.2A3	BioLegend	329702	0.2
165	Ho	CTLA-4	Intracellular	BNI3	BioLegend	369602	0.2
166	Er	KIR3DL2	Surface	539304	R&D systems	MAB2878	0.8
167	Er	Tetramer	Pre-stain	-		-	0.1
168	Er	CCR7	Surface	G043H7	BioLegend	353202	0.8
169	Tm	HK2	Intracellular	EPR20839	Abcam	ab209847	0.8
170	Er	Tetramer	Pre-stain	-		-	0.1
171	Yb	FoxP3	Intracellular	206D	BioLegend	320102	0.8
172	Yb	CD39	Surface	A1	BioLegend	328202	0.4
173	Yb	CD28	Surface	CD28.2	BioLegend	302902	0.8
174	Yb	ATP5A	Intracellular	15H4C4	Abcam	ab14748	0.8
175	Lu	pS6	Intracellular	N7-548	Standard Bio-tools	3175009A	0.8
176	Yb	CPT1A	Intracellular	8F6AE9	Abcam	ab128568	0.2
194	Pt	Lamin A/C	Intracellular	E-1	Santa Cruz Biotechnology	sc-376248	0.05
195	Pt	Viability	Pre-stain	-	Standard Bio-tools	201195	NA
196	Pt	KLRG1	Surface	13F12F2	Invitrogen	16-9488-85	0.8
198	Pt	CD98	Surface	UM7F8	BD Pharmingen	556074	0.4
209	Bi	CD3	Surface	UCHT1	BioLegend	300402	0.2
-	-	Pembrolizumab	Pre-stain	-	Selleckchem	A2005	0.5

Table S4: Mass cytometry peptides used in pMHC tetramers – related to Figure 1

Name	Sequence	Molecular weight (g/mol)	Species	HLA	Protein	Length	NetMHCpan EL 4.1 Score	Channel 1	Channel 2
A01_CMV_UL44_1	VTEHDTLLY	1090	CMV	A*01:01	UL44	9	0.996741	Dy161	Dy163
A01_CMV_pp65_1	YSEHPTFTSQY	1359	CMV	A*01:01	pp65	11	0.979006	Dy161	Er167
A01_InfA_NP_1	CTELKLSDY	1071	Influenza A	A*01:01	NP	9	0.803383	Dy161	Er170
A01_InfA_PB1_1	VSDGGPNLY	921	Influenza A	A*01:01	PB1	9	0.996282	Dy163	Er167
A01_HSV1_UL46_1	ATDSLNNEY	1026	HSV1	A*01:01	UL46	9	0.995817	Dy163	Er170
A01_HSV1_UL48_1	FTDALGIDEY	1143	HSV1	A*01:01	UL48	9	0.991648	Er167	Er170
A02_InfA_M1_1	GILGFVFTL	966	Influenza A	A*02:01	M1	9	0.991397	Dy161	Dy163
A02_EBV_BRLF1_1	YVLDHLIVV	1070	EBV	A*02:01	BRLF1	9	0.97253	Dy161	Er167
A02_EBV_BALF4_1	FLDKGTYTL	1057	EBV	A*02:01	BALF4	9	0.996447	Dy161	Er170
A02_CMV_pp65_1	NLVPMVATV	943	CMV	A*02:01	pp65	9	0.832363	Dy163	Er167
A02_CMV_IE1_1	VLEETSVML	1020	CMV	A*02:01	IE1	9	0.757418	Dy163	Er170
A02_HSV1_UL25_1	FLWEDQTLL	1164	HSV1	A*02:01	UL25	9	0.991397	Er167	Er170
A03_EBV_EBNA-3A_1	RLRAEAQVK	1070	EBV	A*03:01	EBNA-3A	9	0.788046	Dy161	Dy163
A03_CMV_IE1_1	KLGGALQAK	885	CMV	A*03:01	IE1	9	0.886184	Dy161	Er167
A03_HSV1_RS1_1	RLYPDAPPLR	1197	HSV1	A*03:01	RS1	9	0.925983	Dy161	Er170
B07_CMV_pp65_1	RPHERNGFTVL	1325	CMV	B*07:02	pp65	12	0.984307	Dy163	Er167
B07_EBV_BMRF1_1	RPQGGSRPEF	1130	EBV	B*07:02	BMRF1	10	0.951012	Dy163	Er170
B07_InfA_PB1_1	QPEWFRNVL	1188	Influenza A	B*07:02	PB1	9	0.911315	Er167	Er170

Table S5: Neoplastic TCRs – related to Figure 2

CDR3	v_gene	j_gene
CSAYTTGAREETQYF	TRBV20-1	TRBJ2-5
CSARDGRGGRNEQFF	TRBV20-1	TRBJ2-1
CSASLGDERGGARAYNEQFF	TRBV20-1	TRBJ2-1
CASRATYEQYF	TRBV6-1	TRBJ2-7
CASSVTSGQETQYF	TRBV5-1	TRBJ2-5
CASSQRDNSPLHF	TRBV3-1	TRBJ1-6
CSASPLAGGRDTGELFF	TRBV20-1	TRBJ2-2
CASSPPPGLADTQYF	TRBV18	TRBJ2-3
CASTPGGARTEAFF	TRBV19	TRBJ1-1
CATGWNTGELFF	TRBV7-9	TRBJ2-2
CASSLAGGHETQYF	TRBV7-2	TRBJ2-5
CASSLVPGVGNQPQHF	TRBV7-9	TRBJ1-5
CASSGRPGPGIPVGEQYF	TRBV2	TRBJ2-7
