# Supplementary material for: Multi-omic profiling reveals the endogenous and neoplastic responses to immunotherapies in cutaneous T cell lymphoma
Source: Cell Rep Med. 2024 Apr 25;5(5):101527. doi: 10.1016/j.xcrm.2024.101527 (PMC11148639; doi:10.1016/j.xcrm.2024.101527)
Supplement: Document S2. Article plus supplemental information [file mmc3.pdf]

Cell Reports Medicine

Article

Multi-omic profiling reveals the endogenous and neoplastic responses to immunotherapies in cutaneous T cell lymphoma

Graphical abstract

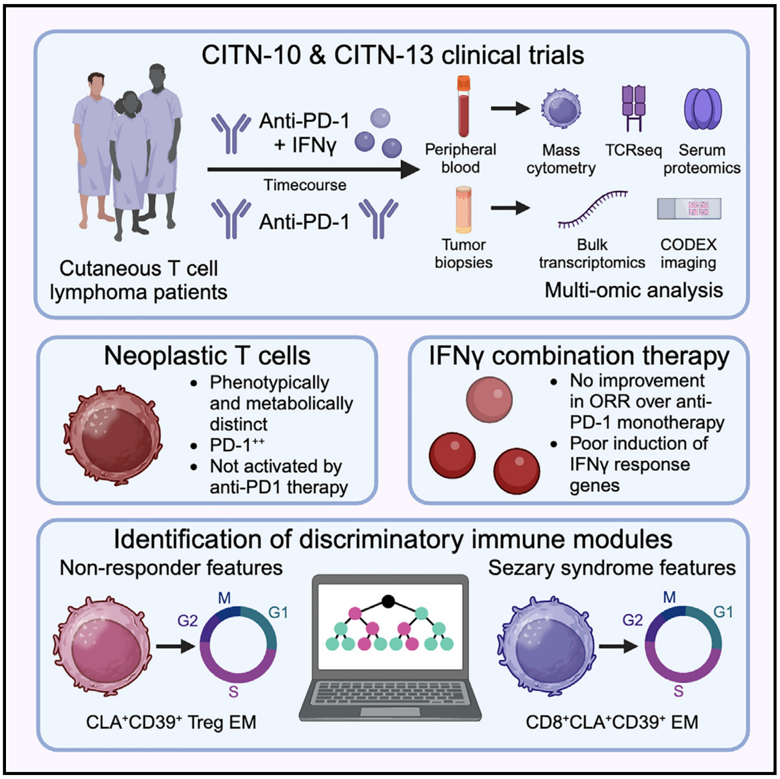

Authors

David R. Glass, Koshlan Mayer-Blackwell, Nirasha Ramchurren, ..., Steven P. Fling, Michael S. Khodadoust, Evan W. Newell

Correspondence

dglass@fredhutch.org (D.R.G.),
enewell@fredhutch.org (E.W.N.)

In brief

Glass et al. perform immune profiling of CTCL patients receiving anti-PD-1 monotherapy or anti-PD-1 + IFN-γ combination therapy. They find neoplastic T cells express high levels of PD-1 but are not activated by anti-PD-1 therapy, while peripheral CLA^+CD39^+ T cell subsets increase in abundance post-therapy and are associated with therapeutic response.

Highlights

- Neoplastic T cells express PD-1 but are not activated by anti-PD-1 therapy
- IFN-γ therapy induces muted endogenous immune responses and fails to improve ORR
- Activation of regulatory CLA^+CD39^+ T cell subsets is enriched in non-responders
- Activation of $CD8^+CLA^+CD39^+$ T cell subsets is enriched in Sezary patients

Glass et al., 2024, Cell Reports Medicine *5*, 101527
May 21, 2024 © 2024 The Authors. Published by Elsevier Inc.
https://doi.org/10.1016/j.xcrm.2024.101527

CellPress

Cell Reports Medicine

CellPress
OPEN ACCESS

Article

Multi-omic profiling reveals the endogenous and neoplastic responses to immunotherapies in cutaneous T cell lymphoma

David R. Glass,[1,*] Koshlan Mayer-Blackwell,[1] Nirasha Ramchurren,[1,2] K. Rachael Parks,[1] George E. Duran,[3] Anna K. Wright,[1,2] Armando N. Bastidas Torres,[3] Laura Islas,[1] Youn H. Kim,[3,4] Steven P. Fling,[1,2,4] Michael S. Khodadoust,[3,4] and Evan W. Newell[1,4,5,*]

[1]Vaccine and Infectious Disease Division, Fred Hutchinson Cancer Center, Seattle, WA 98109, USA
[2]Cancer Immunotherapy Trials Network, Fred Hutchinson Cancer Center, Seattle, WA 98109, USA
[3]Division of Oncology, Stanford University School of Medicine, Stanford, CA 94305, USA
[4]Senior author
[5]Lead contact
*Correspondence: dglass@fredhutch.org (D.R.G.), enewell@fredhutch.org (E.W.N.)
https://doi.org/10.1016/j.xcrm.2024.101527

SUMMARY

Cutaneous T cell lymphomas (CTCLs) are skin cancers with poor survival rates and limited treatments. While immunotherapies have shown some efficacy, the immunological consequences of administering immune-activating agents to CTCL patients have not been systematically characterized. We apply a suite of high-dimensional technologies to investigate the local, cellular, and systemic responses in CTCL patients receiving either mono- or combination anti-PD-1 plus interferon-gamma (IFN-γ) therapy. Neoplastic T cells display no evidence of activation after immunotherapy. IFN-γ induces muted endogenous immunological responses, while anti-PD-1 elicits broader changes, including increased abundance of CLA$^+$CD39$^+$ T cells. We develop an unbiased multi-omic profiling approach enabling discovery of immune modules stratifying patients. We identify an enrichment of activated regulatory CLA$^+$CD39$^+$ T cells in non-responders and activated cytotoxic CLA$^+$CD39$^+$ T cells in leukemic patients. Our results provide insights into the effects of immunotherapy in CTCL patients and a generalizable framework for multi-omic analysis of clinical trials.

INTRODUCTION

Mycosis fungoides (MF) and Sezary syndrome (SS) are the two most common subtypes of cutaneous T cell lymphoma (CTCL).[1] Derived from skin-homing CD4$^+$ memory T cells, these malignancies can manifest as skin patches, plaques, tumors, and inflammation. The presence of circulating neoplastic T cells in blood and lymph nodes is less common in MF, but is a definitive feature of SS. Five-year overall survival rates for patients with advanced MF/SS (stage IIB-IV) range from 0% to 65%, and these figures diminish with increasing disease stage.[2] Checkpoint blockade and CTCL-targeting immunotherapies hold promise for eliciting durable and potentially curative responses.[3] More insight into the *in vivo* effects of these therapies in MF and SS patients is needed to inform patient selection and effective drug combinations.

PD-1 is a checkpoint molecule expressed by activated T cells that limits T cell receptor (TCR) signaling. Blockade of PD-1 or its ligand, PD-L1, enables effective anti-tumor immunity and has shown clinical efficacy in several cancers.[4] Cancer Immunotherapy Trial Network 10 (CITN-10) was a clinical trial testing the efficacy of anti-PD-1 monotherapy in 24 CTCL patients with relapsed or refractory disease. CITN-10 yielded an overall response rate (ORR) of 38%,[5] similar to another recent anti-PD-1 trial with CTCL patients.[6] Interferon-gamma (IFN-γ) is also important for effective anti-tumor immunity, as it enhances antigen presentation but may be limited by the induction of PD-L1 on antigen-presenting cells (APCs).[7] IFN-γ therapy was tested on 16 early- and late-stage CTCL patients with refractory disease and achieved an ORR of 30%.[8] It was reasoned that combining IFN-γ therapy with anti-PD-1 would result in synergistic effects in which IFN-γ would activate the innate immune system, while anti-PD-1 treatment would avoid PD-L1-mediated immune inhibition and enable T cell-mediated tumor clearance. The clinical trial, CITN-13, evaluated the efficacy of IFN-γ and anti-PD-1 combination therapy on 15 CTCL patients with relapsed or refractory disease. However, this combination regimen showed little improvement over anti-PD-1 monotherapy, achieving an ORR of 40%.[9]

Here, we employed a multi-omic systems immunology approach to deeply profile the endogenous and neoplastic immune responses to mono (CITN-10) and combination (CITN-13) therapy in patients with advanced CTCL. We described local, cellular, and systemic immune features pre- and post-IFN-γ and anti-PD-1 therapies and developed a computational framework enabling identification of immune modules differentiating

This is an open access article under the CC BY license (http://creativecommons.org/licenses/by/4.0/).

responders and non-responders as well as SS and MF patients. This study provides insight into the endogenous and neoplastic responses to two immunotherapy regimens in CTCL patients and can serve as a template for multi-omic analysis of primary clinical cohorts.

RESULTS

Multi-omic profiling delineates disease status in CTCL patients

To characterize the immune state and *in vivo* response of CTCL patients receiving immunotherapy, we assembled a cohort of 39 late-stage MF or SS patients enrolled in either CITN-10,[5] testing the efficacy of anti-PD-1 (Pembrolizumab) monotherapy, or CITN-13, testing the efficacy of IFN-γ and anti-PD-1 (Pembrolizumab) combination therapy (Table S1). We focused our analyses on samples taken at baseline (BL), and cycles 1, 2, and 6 (C1, C2, C6), which reflect 1, 3–4, and 15–16 weeks after initiation of therapy (Figure 1A).

To interrogate local tumor effects, we applied NanoString targeted bulk transcriptomics and reanalyzed high-dimensional proteomic imaging data (CODEX)[10] collected on formalin-fixed paraffin-embedded tumor biopsy samples (Figure 1A; Table S2). To quantify systemic effects, we performed Olink-targeted bulk serum proteomics and tracked clonal dynamics by TCR sequencing (TCRseq). We developed a mass cytometry panel to profile peripheral blood mononuclear cells (PBMCs), with a focus on neoplastic and reactive T cells (Tables S3 and S4). Mass cytometry experiments included healthy controls (HCs) (n = 7) selected for HLA-type for identification of viral antigen-specific T cells, so they were not age-matched to CTCL subjects (Figure S1A). Age and gender were not significantly different by disease subtype or therapeutic response (Figures S1B–S1E), but there were significantly more male MF responders and significantly more female SS responders ($q < 0.1$; Figure S1F).

After quality filtering and batch correction (Figure S1G; STAR Methods), we recovered single-cell profiles from 6,323,900 cells, which we clustered into eight major cell types (Figures 1B and S1H; STAR Methods). As expected, neoplastic T cells displayed distinct phenotypic profiles (Figure S1I) and were enriched in SS patients and absent in HCs (Figure 1B). Neoplastic T cell quantitation by mass cytometry was highly and significantly correlated with clinical flow cytometry and TCRseq (Figure S1J). Neoplastic T cells comprised a large proportion of PBMCs in SS patients (mean 47.43%) and were also detectable in some MF patients (Figure 1C). The presence of clonal neoplastic T cells manifested as significantly lower TCR diversity and evenness in SS patients (Figures 1D and S1K). This observation was not confounded by TCR counts (Figure S1L), but SS patients were on average older than MF patients (Figure S1A), which can affect repertoire diversity metrics.[11] After discounting neoplastic TCRs, the repertoire diversity and evenness of SS patients remained lower than MF patients, although not statistically significant (Figures 1D and S1K). After discounting neoplastic T cells, SS patients had fewer CD8$^+$ T cells and more myeloid cells and CTCL patients had fewer CD4$^-$CD8$^-$ T cells than HCs (Figure 1E).

To delineate T cell phenotype and function at different resolutions, we clustered non-neoplastic T cells into 12 coarse populations (e.g., CD8$^+$ naive) and 48 granular populations (e.g., CD4$^+$CLA$^+$CD39$^+$CD103$^+$ Trcm) and provided interpretable cluster annotations and abbreviations (Figure S1M; STAR Methods). Several T cell populations were differentially abundant between HC, MF, and SS subjects, including a noticeable enrichment in CTCL patients for cells expressing cutaneous lymphocyte antigen (CLA), a receptor facilitating skin homing,[12] and CD39, an ectoenzyme often upregulated on tumor-specific T cells[13,14] (Figure S1N).

In summary, multi-omic profiling of CTCL patients from two anti-PD-1 clinical trials revealed differences in immune cell abundances and TCR clonality by disease status.

Neoplastic T cells display no evidence of activation anti-PD-1 therapy

To evaluate neoplastic T cell phenotypes, we projected an equal sampling of neoplastic T cells from leukemic subjects (those with >0.9% neoplastic T cells) onto a UMAP[15] (Figure 2A). Cells largely co-localized by subject, indicating greater inter-subject heterogeneity than intra-subject heterogeneity. While the majority of neoplastic T cells displayed a skin-tropic memory phenotype (CLA$^+$CD45RO$^+$), we also observed both CD45RO$^-$ and CLA$^-$ cells. Neoplastic T cells exhibited substantial expression of checkpoint molecules, as well as other markers noted by others, including CD39, CCR4, and KIR3DL2.[16–20]

To compare neoplastic T cell phenotypes to normal, non-naive CD4$^+$ T cells, we performed differential expression analysis of their median protein expression profiles (Figure 2B). Strikingly, the majority of proteins queried were significantly differentially expressed (27 of 37 proteins; $q < 0.1$), primarily enriched on neoplastic T cells (21 of 27 proteins). Hierarchical clustering of differentially expressed proteins stratified the cell populations with 94% accuracy (Figure S2A).

Neoplastic T cells are quantified by clinical flow cytometry as CD4$^+$CD26$^-$,[21] but many normal CD4$^+$ T cells are CD26$^-$ (Figure 2C) and we observed one patient with CD26$^+$ neoplastic T cells (Figure 2A).[22] To generate an improved gating strategy, we applied linear discriminant analysis with hybrid subset selection (HSS-LDA)[23,24] to discriminate neoplastic and normal CD4$^+$ T cells in leukemic CTCL patients and HCs (Figure S2B). To avoid bias, we excluded patients who had previously been treated with anti-CCR4 and anti-KIR3DL2 therapy. HSS-LDA identified CD39, CD95, and KIR3DL2 as the three most discriminatory markers, yielding a classification accuracy of >90%. We converted this classifier into a gating scheme in which CD4$^+$ neoplastic T cells were gated as CD95$^-$(CD39$^+$ or KIR3DL2$^+$) and compared the purity and accuracy of this gate to the clinical CD26$^-$ gate (Figure 2C). Our gating strategy significantly increased true negatives, decreased false positives, and had higher overall accuracy (Figure S2C). To validate our marker choice, we employed the Hypergate algorithm,[25] which also selected CD39 and CD95 as the most discriminatory markers (Figure S2D).

FOXP3, a transcription factor that can be expressed by both regulatory T cells (Tregs)[26] and activated, non-regulatory CD4$^+$ T cells[27] had higher expression in neoplastic T cells (Figure 2B). To test if FOXP3^{hi} neoplastic T cells adopted a Treg phenotype, we gated these cells and compared their expression of canonical Treg markers to non-naive CD4$^+$ T cells, Tregs, and

CellPress
OPEN ACCESS

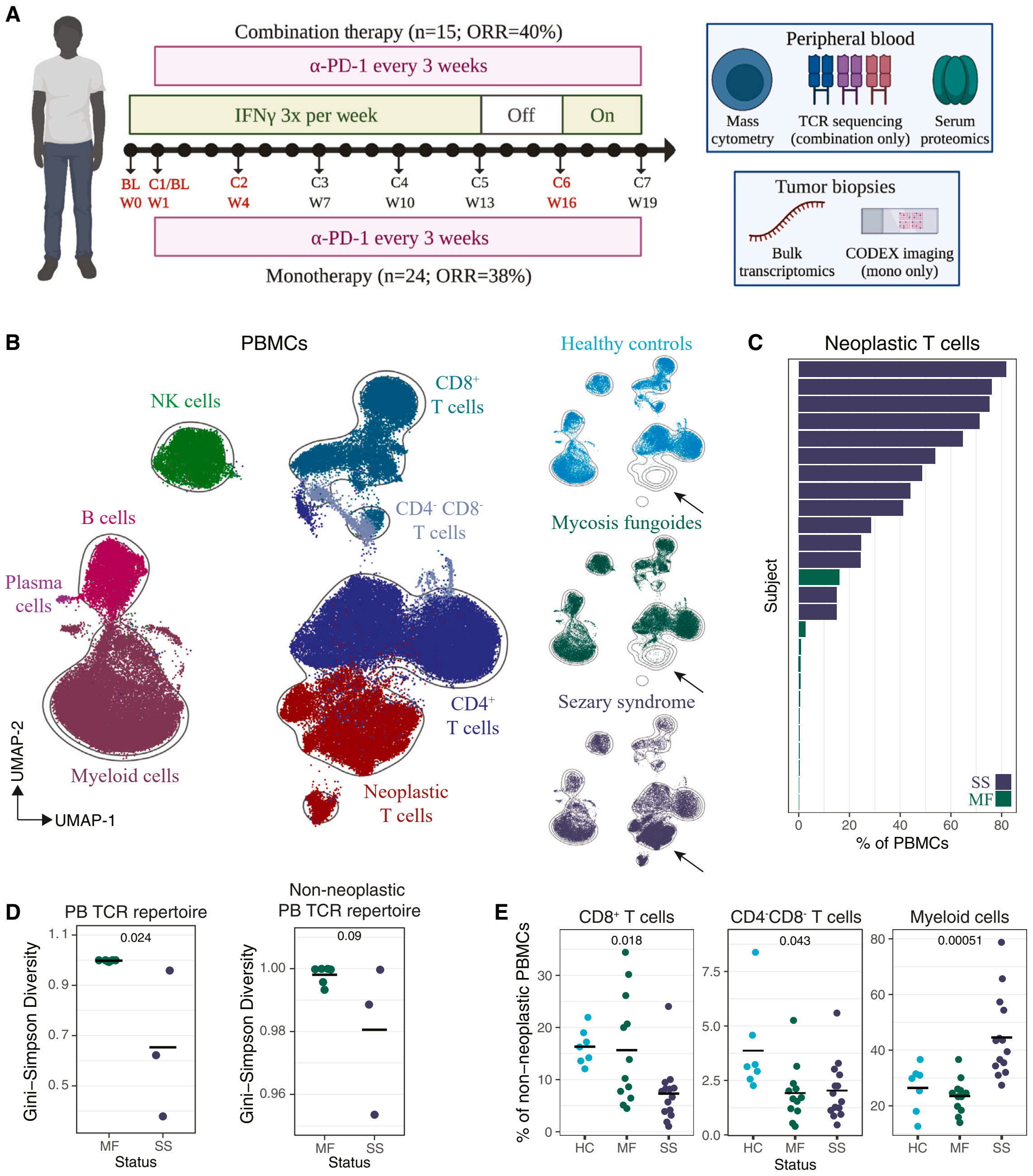

Figure 1. Multi-omic profiling delineates disease status in CTCL patients

(A) Diagram of clinical trial time course and experimental approaches.

(B) UMAP of an equal sampling of PBMCs from HC, MF, and SS subjects' BL samples. Colored by cell type or disease status.

(C) Percent neoplastic T cells for each CTCL subject, colored by disease status.

(D) Gini-Simpson diversity of blood TCR repertoire at BL by disease status, with or without neoplastic clonotypes. Crossbar indicates mean. Wilcoxon rank sum.

(E) BL non-neoplastic frequencies by disease status. Crossbar indicates mean. FDR-corrected Kruskal-Wallis.

FOXP3$^{lo/-}$ neoplastic T cells (Figure 2D). FOXP3^{hi} neoplastic T cells had increased expression of ICOS, but did not adopt prototypical expression patterns associated with Tregs (e.g., CD25^{+}CD127^{-}).

One hallmark of cancer is the reprogramming of cellular metabolism to support cell growth and proliferation.[28] We observed that all five metabolic regulators quantified in our mass cytometry panel (ATP5A, CD98, CPT1A, HK2, and VDAC1), representing five different metabolic pathways (tricarboxylic acid cycle, amino acid metabolism, fatty acid metabolism, glycolysis, and mitochondrial dynamics),[29] were significantly upregulated in neoplastic T cells (Figure 2B). To investigate their

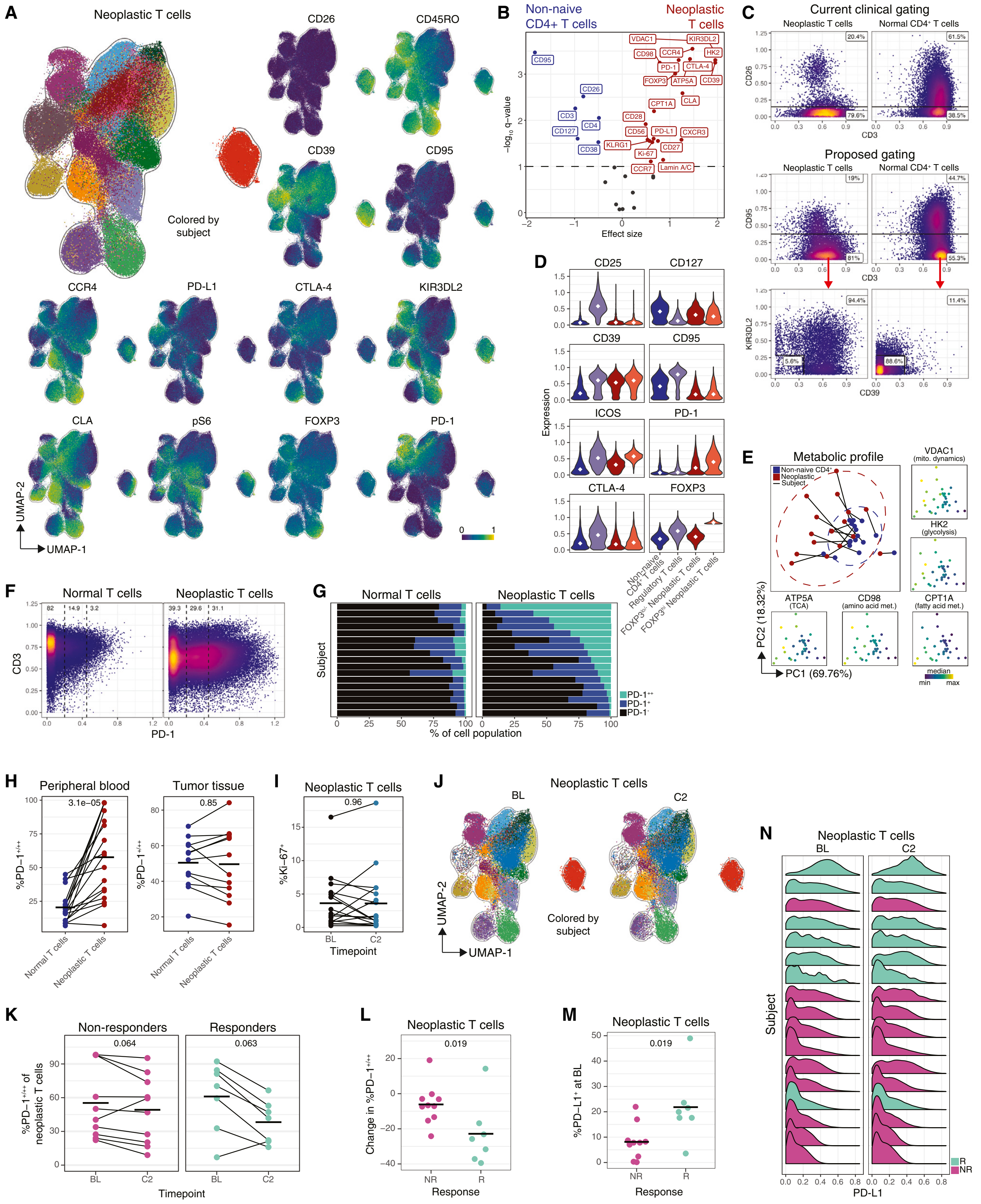

(legend on next page)

high-dimensional metabolic profiles, we performed principal-component analysis (PCA) on the medoids of neoplastic T cells and normal, non-naive $CD4^+$ T cells (Figure 2E). Neoplastic T cells had larger confidence interval (CI) ellipses than normal T cells, indicating greater diversity of metabolic expression values between subjects. Lower PC1 values were indicative of higher metabolic expression values (Figure S2E) and, within each subject, neoplastic medoids always had lower PC1 values than their normal counterparts (Figure S2F), yet the median metabolic expression across regulators was highly correlated between neoplastic and normal T cells from the same subject (Figure S2G). Notably, the metabolic profiles of neoplastic T cells changed minimally in response to anti-PD-1 therapy (Figure S2H).

The checkpoint molecules CTLA-4, PD-1, and PD-L1 were all significantly upregulated in neoplastic T cells compared with non-naive $CD4^+$ T cells (Figures 2B, S2I, and S2J). Within blood, we observed substantially greater expression of PD-1 on neoplastic T cells over normal T cells across and within patients (Figures 2F, 2G, and S2K–S2M). There was, however, no difference in expression between the two populations in the tumor due to upregulation of PD-1 by normal T cells in the tumor, as reported previously[30,31] (Figure 2H).

Post-anti-PD-1 therapy, there was a small but significant decrease in the frequency of neoplastic T cells (Figure S2N). Despite the substantial expression of PD-1, we observed no change in expression of Ki-67 (Figure 2I), HLA-DR (Figure S2O), or any other activation-associated markers (Figure S2P) by neoplastic T cells post-therapy. In fact, we observed no discernable shift in overall cell phenotype, as evident by the common localizations of cells in UMAP space pre- and post-therapy (Figure 2J). The only protein significantly differentially expressed was PD-1, which was expressed by a smaller frequency of neoplastic T cells post-therapy (Figures S2P and S2Q). This observation was not an artifact of steric hindrance of the PD-1 epitope, as all samples were pre-stained with Pembrolizumab and PD-1 was quantified using an anti-human IgG4 secondary antibody, as described previously[32] (Table S3; STAR Methods). Both responders and non-responders had lower proportions of $PD\text{-}1^+$ neoplastic T cells post-therapy (Figure 2K), although the magnitude of difference was significantly greater for responders (Figure 2L). We then asked if any other neoplastic cell-intrinsic features stratified responders and non-responders and found only the expression of PD-L1 was significantly higher in responders, both pre- and post-therapy (Figures 2M and 2N).

We then investigated if neoplastic T cells shifted phenotype under the selective pressure of anti-PD-1 therapy. We found there were significant increases in the proportion of $CD38^+$ and $CD56^+$ neoplastic T cells at C6, 15 weeks post-therapy (Figures S2R and S2S), but no other changes at that time point reached statistical significance. Limiting this analysis to only non-responders yielded similar results (data not shown). As expected, we observed a substantial reduction of neoplastic T cells at C6 in responders, but not in non-responders (Figure S2T).

We investigated neoplastic TCR sequences (Table S5) for unique properties. We quantified the probability of generation (pgen)[33] and found no statistical difference between neoplastic and endogenous TCRs (Figure S2U). Neoplastic TCRs had no known antigen specificity reported in VDJdb,[34] nor any consistent gene usage or common CDR3 motifs based on the TCRdist metric.[35]

In summary, we observed substantial differences in the expression patterns of neoplastic T cells compared with normal T cells, including significantly higher levels of PD-1, but found no evidence of neoplastic activation or phenotypic shift induced by anti-PD-1 therapy.

IFN-γ induces muted immune activation

It was hypothesized that IFN-γ therapy would activate the innate immune system and that anti-PD-1 treatment would overcome IFN-γ-induced, PD-L1-mediated immune inhibition, enabling T cell-mediated tumor clearance. Combination therapy, however, exhibited little improvement in response (ORR = 40%) over monotherapy (ORR = 38%). To understand the lack of synergy between therapeutic agents and to investigate the *in vivo* effects of IFN-γ monotherapy, we examined the changes induced at the C1 time point.

There was no significant induction of PD-L1 in any cell population and no individual demonstrated substantial upregulation of PD-L1, regardless of response and disease status (Figures 3A and S3A). Likewise, there was no significant

Figure 2. Neoplastic T cells display no evidence of activation anti-PD-1 therapy

(A) UMAP of an equal sampling of neoplastic T cells from leukemic subjects. Colored by subject or protein expression.

(B) Differentially expressed markers between neoplastic and normal non-naive $CD4^+$ T cells based on median expression. Colored by enriched population. Annotated markers are significant ($q < 0.1$). FDR-corrected Wilcoxon signed rank.

(C) Current (top) and proposed (middle, bottom) clinical gating schemes for neoplastic and normal $CD4^+$ T cells from an equal sampling of cells from HC and leukemic subjects, colored by density. Numbers indicate percent in gate.

(D) Expression levels of an equal sampling of indicated T cell populations. Diamond indicates median.

(E) PCA derived from median expression of metabolic markers in neoplastic and normal non-naive $CD4^+$ T cells, colored by cell or median expression. Ellipses indicate 95th percentile CI assuming a multivariate t-distribution.

(F) Biaxials of an equal sampling of normal or neoplastic T cells at BL, colored by density. Numbers indicate percent in gate.

(G) %PD-1 expression by normal or neoplastic T cells by subject.

(H) $\%PD\text{-}1^{+/++}$ normal or neoplastic T cells in PB or tumor. Crossbar indicates mean. Wilcoxon signed rank.

(I) $\%Ki\text{-}67^+$ neoplastic T cells by time point. Crossbar indicates mean. FDR-corrected Wilcoxon signed rank.

(J) UMAP of an equal sampling of neoplastic T cells; same manifold as (A). Colored by subject and separated by time point.

(K) $\%PD\text{-}1^+/^{++}$ neoplastic T cells by time point and response. Crossbar indicates mean. FDR-corrected Wilcoxon signed rank.

(L) Change in $\%PD\text{-}1^+/^{++}$ neoplastic T cells at C2. Crossbar indicates mean. Wilcoxon rank sum.

(M) $\%PD\text{-}L1^+$ neoplastic T cells at BL by response. Crossbar indicates mean. Wilcoxon rank sum.

(N) PD-L1 expression in neoplastic T cells by time point. Rows ordered by BL $\%PD\text{-}L1^+$ and colored by response.

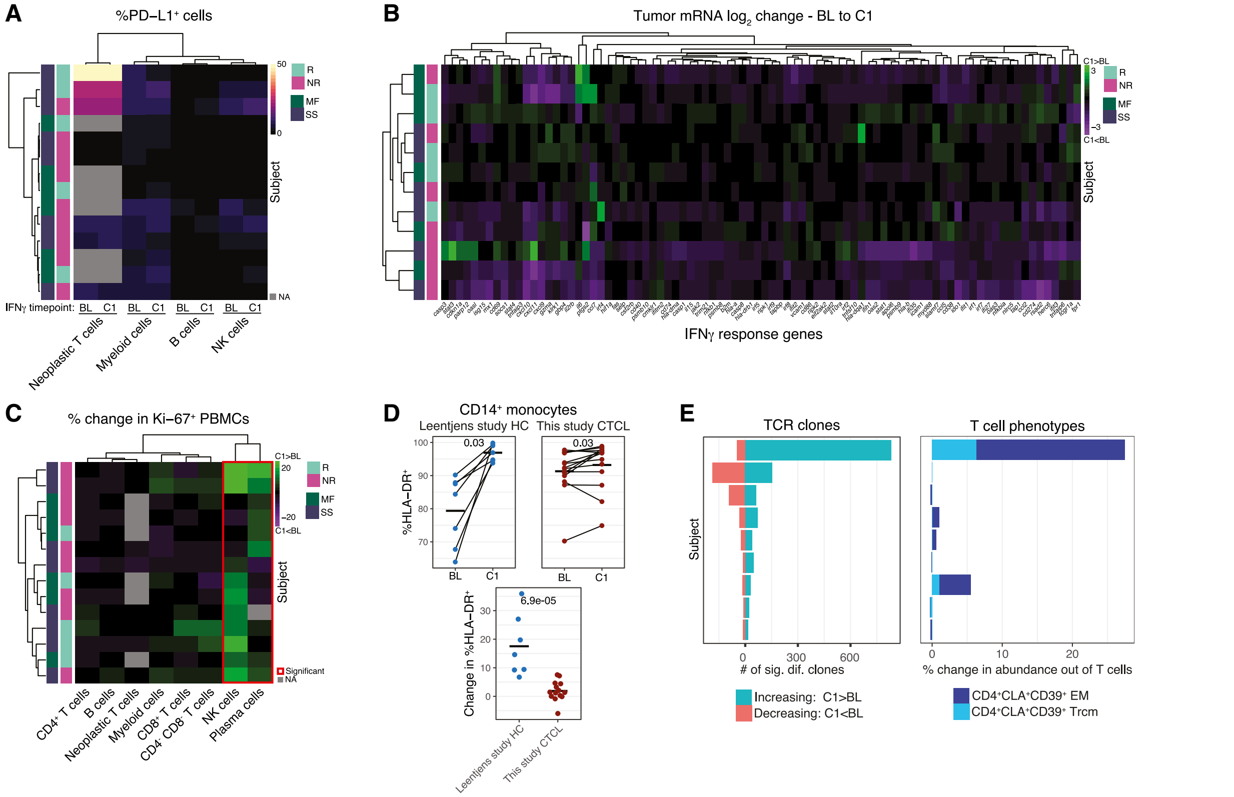

Figure 3. IFN-γ induces muted immune activation

(A) %PD-L1$^+$ cells by subject, cell type, and time point.

(B) Log_2 expression change of IFN-γ response genes in tumor mRNA between BL and C1, by subject.

(C) Change in %Ki-67$^+$ cells by cell type at C1. Red box indicates significance ($q < 0.1$). FDR-corrected Wilcoxon signed rank.

(D) %HLA-DR$^+$ CD14$^+$ monocytes by time point and disease status (top). Wilcoxon signed rank. Change in %HLA-DR$^+$ CD14$^+$ monocytes at C1 by disease status (bottom) Wilcoxon rank sum.

(E) Significantly differentially abundant TCR clone counts ($q < 0.1$) between time points (left) and change in T cell subset frequency out of non-neoplastic T cells between time points (right). Subject order is conserved. FDR-corrected Fisher's exact.

upregulation of PD-L1 or PD-L2 tumor mRNA or serum protein (Figure S3B), although responders had significantly higher PD-L2 mRNA in the tumor post-therapy (Figure S3C).

We also found no significant upregulation of major histocompatibility complex (MHC) or IFN-γ response genes in the tumor biopsy samples and no emergent organization by response or disease status after hierarchical clustering of IFN-γ response gene log_2 change values (Figure 3B). Most patients actually had lower mean expression of IFN-γ response genes post-therapy (Figure S3D). Likewise, no IFN-γ response serum proteins were significantly upregulated post-therapy (Figure S3E), nor was IFN-γ itself (Figure S3F). MARCO, a pattern recognition receptor expressed by macrophages,[36] was the only serum protein significantly upregulated post-IFN-γ therapy (Figure S3G). Several other serum proteins were significantly downregulated as was one tumor mRNA species, *hdac5*, a class IIa histone deacetylase (HDAC) (Figure S3H).[37] Several HDAC inhibitors are approved for the treatment of CTCL,[38] although repression of HDAC5 can have pro-angiogenic effects.[39]

We observed significantly increased cell cycling in NK and plasma cells (Figure 3C) but no significant changes in PBMC subset abundances (Figure S3I). To understand the muted responses of myeloid cells, we utilized a dataset from a study investigating IFN-γ as a treatment for immunoparalysis.[40] HCs received intravenous LPS and then three subcutaneous injections of IFN-γ over the course of 6 days, before receiving a second dose of LPS. We compared the pre-LPS time points, before and after IFN-γ therapy, with the current study. While monocytes from both HCs and CTCL patients significantly upregulated HLA-DR after administration of IFN-γ, the magnitude of upregulation was significantly greater in HCs (Figure 3D). HCs from both the Leentjens study[40] and the current study had significantly lower expression of HLA-DR on monocytes than CTCL patients at BL (Figure S3J), enabling upregulation after therapy. Indeed, the difference in %HLA-DR$^+$ monocytes between HCs and CTCL patients was abrogated after IFN-γ therapy (Figure S3K).

It was also hypothesized that IFN-γ would bias the immune response toward a Th1 response, but we observed no significant upregulation of CXCR3, the canonical Th1 surface marker, in any CD4$^+$ populations and only a slight upregulation of CXCR3 in CD8$^+$ CM T cells (Figure S3L). The only significant changes in T cell abundances post-therapy were diminished proportions

of total MAIT/NKT and CD8$^+$ KLRG1$^+$ MAIT/NKT cells (Figure S3M). Both $\gamma\delta^+$ T cells and CD8$^+$ EMRAs, however, significantly increased the proportion of cycling cells post-therapy (Figure S3N). CD8$^+$ EMRAs also significantly upregulated VDAC1 and ATP5A post-therapy (Figure S3O), indicating a bias toward oxidative phosphorylation. No significant changes in expression of PD-1, CTLA-4, or HLA-DR were observed in any T cell population between pre- and post-IFN-γ time points (data not shown).

Finally, we asked if IFN-γ altered the TCR repertoire. The number of significantly differentially abundant TCR clones between time points varied greatly between patients (Figure 3E). In one SS responder, 825 clones significantly increased in abundance post-therapy (compared with a median of 21 clones), which coincided with a dramatic increase in the proportion of CD4$^+$CLA$^+$CD39$^+$ EM and CD4$^+$CLA$^+$CD39$^+$ Trcm cells, suggesting that many of these clones represent T cells displaying these phenotypes. We queried clones of interest (STAR Methods) for known specificity but found no matches in the VDJ database.[34] To identify TCR clones potentially recognizing common antigens, we performed TCR sequence similarity clustering by TCRdist3 on clones of interest. We grouped clones into metaclonotypes, defined as clusters of sequences with shared TRBV gene usage and highly biochemically similar CDR3s (STAR Methods; Figure S3P).[35] We identified three metaclonotypes comprised of four or more clones in which all member clones increased in abundance post-IFN-γ therapy (Figure S3Q). One metaclonotype was shared by two individuals and included multiple clones present in both the tumor and periphery. We report those CDR3 sequences, as these metaclonotypes may represent tumor-specific TCRs with therapeutic utility (Figure S3R).

In summary, IFN-γ monotherapy induced muted and inconsistent induction of IFN-γ response genes, PD-1 ligands, and cellular activation.

CLA$^+$CD39$^+$ T cells resemble TILs and increase in circulation after anti-PD-1 therapy

We then asked how anti-PD-1 therapy altered the immune states of patients across both trials. We observed significant downregulation of PD-1 post-anti-PD-1 therapy in both neoplastic and normal T cells (Figure 4A). Indeed, 34 normal T cell subpopulations significantly downregulated PD-1 post-therapy (Figure S4A), although serum PD-1 and PD-1 tumor mRNA were not significantly changed (Figure S4B).

The composition of major PBMC populations was not significantly altered by anti-PD-1 therapy (data not shown), so we queried the T cell compartment. Four CLA$^+$CD39$^+$ T cell populations, representing CD4$^+$ and CD8$^+$ EM and CM T cells, were significantly more abundant after anti-PD-1 therapy (Figure 4B). We projected non-neoplastic T cells onto a UMAP and found that the four CLA$^+$CD39$^+$ populations expressed high levels of CCR4, a skin-homing chemokine receptor,[41] as well as several activation and checkpoint molecules (Figure 4C). Indeed, CD39, CLA, and CCR4 expression were highly correlated, as were HLA-DR and Ki-67 (Figure S4C). Our panel included peptide-MHC (pMHC) tetramers to identify bystander, cytomegalovirus (CMV)-specific T cells (Figure S4D). CMV-specific T cells did not co-localize on the UMAP with CLA$^+$CD39$^+$ T cells (Figure 4C), despite their enrichment for the EM phenotype (Figure S4E), and did not express CLA or CD39 (Figure S4F).

CMV-specific CD8$^+$ EM T cells had only modest levels of activation at BL, and therapy had little effect on that activation profile (Figure 4D). In contrast, CLA$^+$CD39$^+$CD8$^+$ EM T cells displayed substantial levels of activation, which diminished post-IFN-γ therapy, and rebounded after anti-PD-1 therapy. Across a range of CD4$^+$ and CD8$^+$ subpopulations at BL and C2, CLA$^+$CD39$^+$ T cells had significantly higher expression of CD38, HLA-DR, Ki-67, and PD-1 as compared with their CLA$^-$CD39$^-$ counterparts (Figures 4E and S4G). We then asked how these patterns compared with tumor-infiltrating lymphocytes (TILs) at BL, as measured by CODEX imaging. Both CD4$^+$ and CD8$^+$ TILs expressed significantly more HLA-DR and PD-1 than CLA$^-$CD39$^-$ T cells in the blood (Figure 4F). CD4$^+$ TILs expressed similar levels of HLA-DR and PD-1 to their CLA$^+$CD39$^+$ counterparts in the blood, while CD8$^+$ TILs had significantly higher PD-1 and significantly lower HLA-DR than CD8$^+$CLA$^+$CD39$^+$ peripheral T cells.

We then compared the number of significantly differentially abundant clones pre- and post-IFN-γ therapy with those pre- and post-anti-PD-1 therapy. There were significantly more differentially abundant clones after anti-PD-1 therapy (Figure S4H), although the larger time period (3 weeks versus 1 week) is confounding. We observed no difference in TCR repertoire similarity between time points when stratified by therapeutic response (Figure S4I).

We performed differential expression analysis to investigate conserved changes in tumor mRNA and serum protein after anti-PD-1 therapy. We found no significantly differentially expressed genes in our bulk transcriptomics data, although several genes related to immune activation and antigen presentation were upregulated (Figure S4J). MPO, an anti-microbial peroxidase highly expressed by neutrophils and monocytes,[23] and CLEC1B, a C-type lectin receptor also expressed by myeloid cells,[42] were significantly more abundant in serum post-anti-PD-1 therapy (Figure S4K).

In summary, anti-PD-1 therapy increased the abundance of several activated CLA$^+$CD39$^+$ T cell populations in blood, which were not specific to bystander CMV pMHC tetramers and had similar expression profiles to TILs.

Multi-omic immune modules identify features associated with therapeutic response

To discover features differentiating responders from non-responders, we developed a computational framework enabling integration of our high-dimensional, multi-omic datasets and identification of discriminatory immune modules (STAR Methods; Figure 5A). We compiled serum protein values, tumor transcriptomics values, and single-cell summary statistics from both trials into a consolidated feature set. For each time point parameter (BL, C2, and the difference between C2 and BL), we calculated pairwise correlation of all multi-omic features and clustered those features into immune modules based on their weighted correlation coefficient vectors. We performed PCA on features within each immune module and used PC1 to summarize that immune module, as PC1 explained the majority of the variance of the underlying features (Figure S5A). We stratified

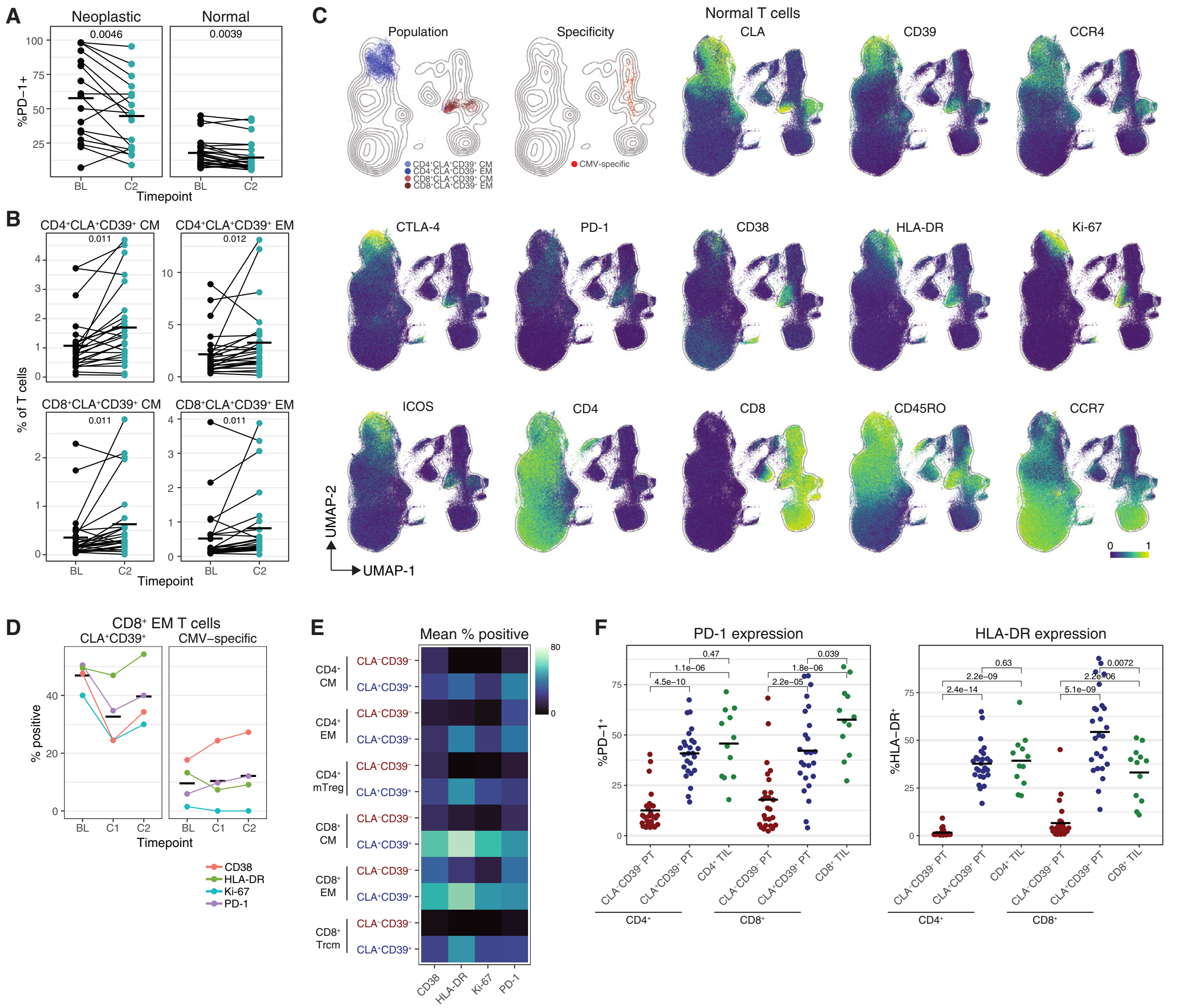

Figure 4. CLA$^+$CD39$^+$ T cells resemble TILs and increase in circulation after anti-PD-1 therapy

(A) %PD-1$^+$ neoplastic and normal T cells by time point. Crossbar indicates mean. FDR-corrected Wilcoxon signed rank.

(B) T cell population frequency out of non-neoplastic T cells by time point. Crossbar indicates mean. FDR-corrected Wilcoxon signed rank.

(C) UMAP of non-neoplastic T cells from an equal sampling of subjects pre- and post-anti-PD-1 treatment. Colored by population, specificity, or marker expression.

(D) %$^+$ activation markers from CLA$^+$CD39$^+$ or CMV-specific CD8$^+$ EM T cells from one subject across time points. Crossbar indicates mean.

(E) Mean %$^+$ activation markers across CLA$^+$CD39$^+$ and CLA$^-$CD39$^-$ T cell subsets.

(F) %$^+$ T cells by cell type at BL. Crossbar indicates mean. FDR-corrected Wilcoxon rank sum.

patients by therapeutic response, trained random forest classifiers to discriminate the two groups with immune modules as inputs, and evaluated the importance of each immune module by calculating Gini index. We performed this approach independently for the three time point parameters but focused our analysis on the C2, post-anti-PD-1 time point, as it had the lowest out-of-bag (OOB) error, a metric of classifier accuracy (Figure S5B). We only considered immune modules with variable importance above a minimum threshold for further investigation (Figure 5B).

The model identified nine immune modules comprised of 16 features discriminating responders from non-responders at C2 (Figure 5C). Responders were enriched for higher serum levels of the cytotoxic molecules GZMA, GZMB, and GZMH, as well as higher expression of activation molecules on CD8$^+$CLA$^+$ EM T cells. Responders also had higher serum abundances of the chemokines CCL2, CXCL1, and CXCL6, associated with myeloid trafficking and activation.[43] Non-responders had higher proportions of cell cycling in both total Tregs and a CLA$^+$CD39$^+$ EM subset of Tregs, as well as greater HLA-DR

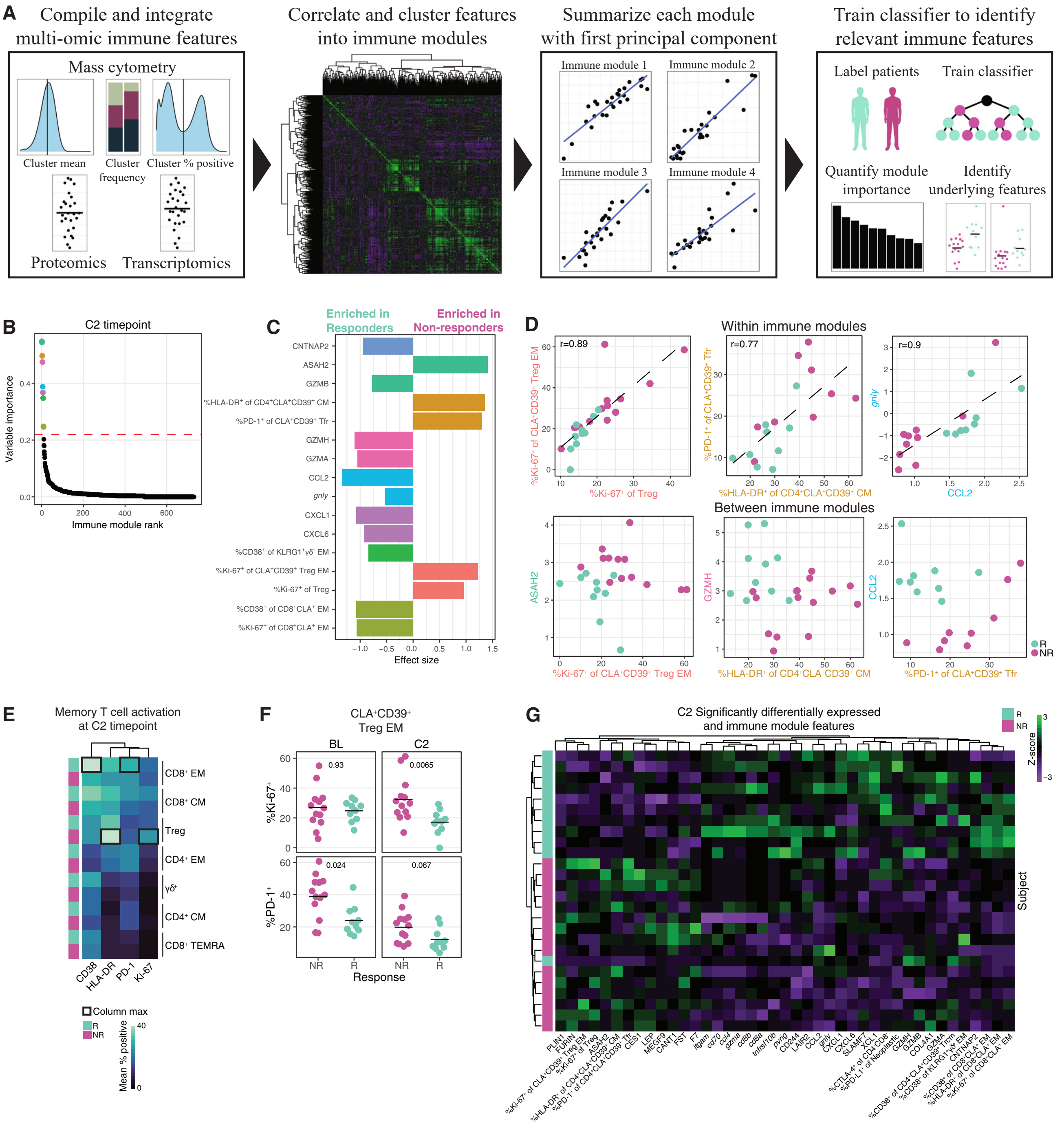

Figure 5. Multi-omic immune modules identify features associated with therapeutic response

(A) Diagram of immune modules approach.

(B) Gini index of predictors separating response and non-response at C2. Dashed line indicates threshold for differential immune modules. Colors same as (C).

(C) Effect size of features comprising immune modules selected in (B). Color denotes immune module. Rows ordered by Gini index.

(D) Differential immune features in the same (top) or different (bottom) immune modules. R calculated by Pearson method. Fitting line calculated by linear regression.

(E) Mean %$^+$ activation markers for T cell populations by response.

(F) %$^+$ of CLA$^+$CD39$^+$ Treg EM by response and time point. Crossbars indicate mean. FDR-corrected Wilcoxon rank sum.

(G) *Z* scores of features selected by model and/or significantly differentially expressed at C2.

expression on $CD4^+CLA^+CD39^+$ CM T cells and greater PD-1 expression on CLA^+CD39^+ Tfr cells. These two features clustered into the same immune module as they were highly correlated (Figure 5D), suggesting some biological co-regulation such as response to a common stimulus or phenotypic plasticity between the subsets. Immune modules also captured highly correlated inter-tissue/inter-omic features, such as granulysin (*gnly*) mRNA expression in tumor biopsy samples and serum CCL2 protein, which were higher in responders. Whereas features within the same immune module were highly correlated, features from different immune modules provided enhanced separation of patient groups, as the model selected combinations of immune modules that stratify patients. For example, patients with high expression of PD-1 on CLA^+CD39^+ Tfr cells were all non-responders, but patients with a low or intermediate PD-1 expression on CLA^+CD39^+ Tfr were non-responders only if they also had low levels of serum CCL2 (Figure 5D, lower right).

The expression of activation markers varied among T cell subsets between responders and non-responders (Figure 5C). We further explored post-therapy T cell activation profiles and found the highest proportions of CD38 and PD-1 expression in responders' $CD8^+$ EM T cells, and the highest proportions of HLA-DR and Ki-67 expression in non-responders' Tregs (Figure 5E). Non-responders exhibited significantly more cell cycling in CLA^+CD39^+ Treg EM cells at C2, but not at BL (Figure 5F). We also observed no difference in Treg abundance or activation in the tumors of responders and non-responders at BL (Figure S5C). Interestingly, we found significantly more PD-1 expression in CLA^+CD39^+ Treg EM cells at BL, potentiating activation after administration of anti-PD-1 therapy (Figure 5F).

In addition, we performed differential distribution analysis on our multi-omic feature set, which replicated several findings from our immune modules model and yielded additional hits (Figure S5D). Responders were enriched for several immune transcripts in the tumor mRNA, so we utilized a public single-cell RNA sequencing (scRNA-seq) dataset conducted on CTCL tumor samples to identify the cell populations expressing genes of interest from both the statistical and model approaches (Figure S5E).[44] Responders were enriched for *cd8a*, *cd8b*, *cd70*, *gnly*, and *gzma* transcripts, which were predominantly expressed by $CD8^+$ T cells, although we also detected the cytotoxic genes *gzma* and *gnly* in neoplastic T cells and Tregs. There was also significantly more *itgam* and *ccl4* in responders, predominantly expressed by macrophages.

To explore the co-occurrence of features selected by our two approaches, we visualized the *Z* scores of differential features (Figure 5G). Unsupervised hierarchical clustering resulted in near perfect stratification of responders and non-responders, highlighting the local, cellular, and systemic immune features characteristic of a successful therapeutic response.

We performed the same immune module and statistical approaches to stratify responders and non-responders at BL (Figures S5F–S5H). Several features were enriched in responders at both BL and C2, including increased abundance of serum CCL2 and GZMA and tumor *gzma* and *gzmh* mRNA. Responders had higher BL expression of activation markers in $CD8^+CLA^+$ EM T cells, although the observation did not reach statistical significance (Figure S5I). In addition, we performed our immune module and statistical approaches to stratify responders and non-responders at the differential of C2 and BL features, representing the change that occurred in each feature between the time points (Figures S5J–S5L).

In summary, we developed a computational framework enabling discovery of immune modules stratifying responders and non-responders. Post-anti-PD-1 therapy, we found that non-responders were enriched for activation of CLA^+CD39^+ Treg populations, while responders had higher abundances of cytotoxic molecules in serum and tumors, as well as greater activation of $CD8^+CLA^+$ EM T cells.

Mono and combination therapy induce differential abundance of PD-1

While there was little difference in ORR of the two clinical trials (Figure 1A), we asked if there were differential immune features. To account for BL differences between cohorts, we analyzed changes induced between BL and C2. We applied our immune modules approach and our models had exceedingly low OOB errors (Figure S6A). We identified four immune modules predictive of therapy regimen, comprising seven features (Figures S6B and S6C). The model selected only changes in serum protein abundances, and the strongest predictor was serum PD-1. In the monotherapy, there was a significant decrease in serum PD-1 after anti-PD-1 therapy, consistent in every patient, while in combination therapy there was a significant increase in serum PD-1 (Figure S6D). This increase in serum PD-1 was not evident at the post-IFN-γ C1 time point. We observed no difference in PD-1 induction in tumor mRNA (Figure S6E) and there was significant downregulation of cellular PD-1 in both regimens (Figure S6F). One outlier receiving combination therapy had a decrease in serum PD-1 after anti-PD-1 therapy (Figure S6D). This SS patient was an NR with the highest serum PD-1 and the highest cellular PD-1 expression at BL and C1, but unremarkable levels of PD-1 tumor mRNA (Figures S6D–S6F). We identified five populations, comprised of various CM and EM subsets, that downregulated PD-1 significantly more in response to combination therapy (Figure S6G). We also observed significantly more downregulation of the activation marker, HLA-DR, in two populations, $CD39^+$ Treg EM and $CD8^+KLRG1^+CD57^+$ EM T cells, after combination therapy, although the magnitude of the difference between therapy regimens was smaller than for PD-1.

Finally, we applied our statistical approach to identify additional features differentially changed from BL to C2 between the two therapy regimens. We identified many additional immune features (Figure S6H), including the tumor mRNA abundance of the transcription factor, *irf4*, which was substantially downregulated in combination, but not monotherapy. Conversely, the inhibitory receptor, FCRL6, was substantially upregulated in the serum of patients receiving combination, but not monotherapy. In summary, we identified many immune features differentially induced between combination and monotherapy, most notably serum PD-1, which was significantly downregulated after monotherapy and significantly upregulated after combination therapy.

CellPress
OPEN ACCESS

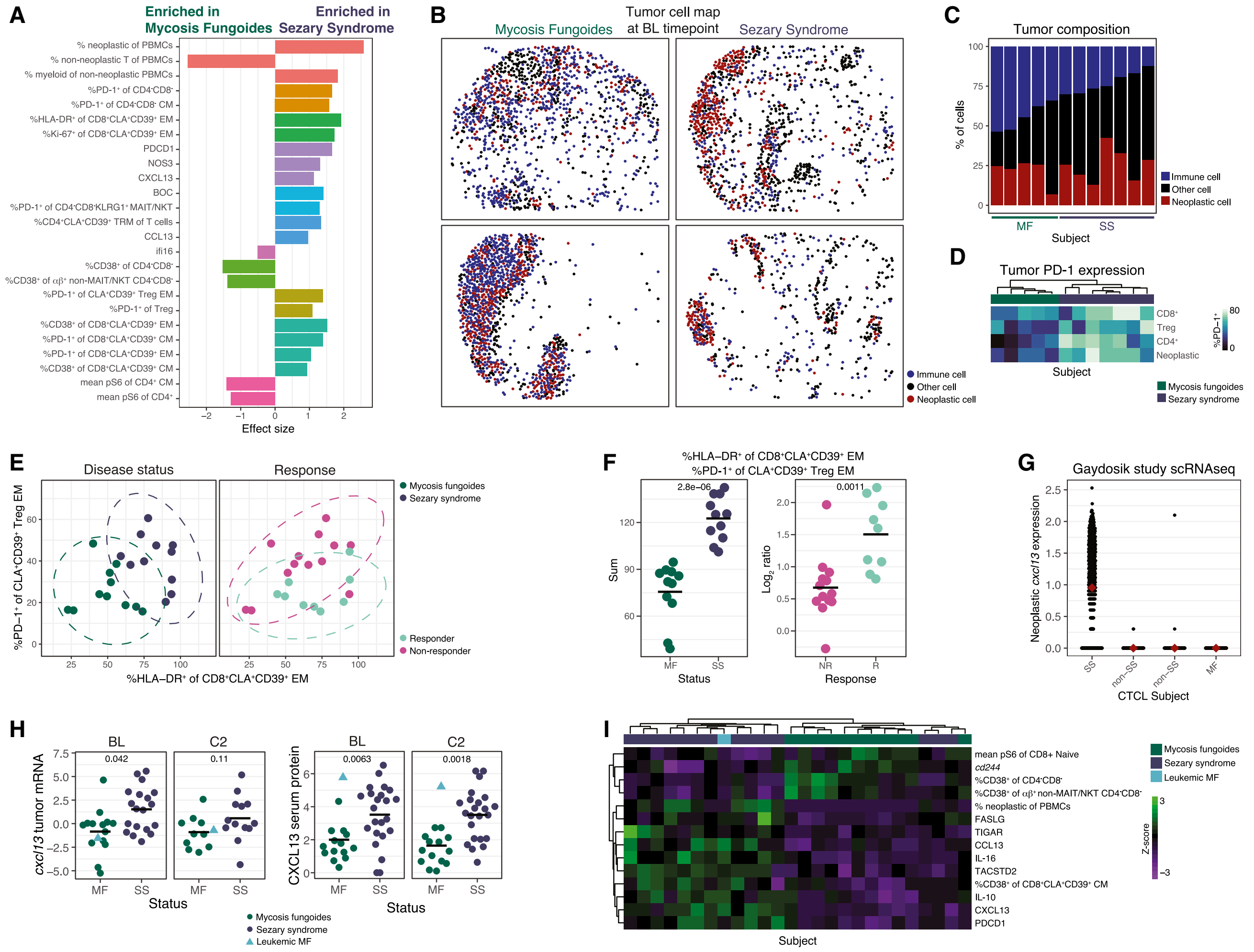

Figure 6. Systemic and local immune features differentiate MF and SS patients pre- and post-anti-PD-1 therapy

(A) Effect size of features comprising immune modules differentiating MF and SS patients at BL, colored by immune module, Rows ordered by Gini index.

(B) Tumor cell map showing coordinates of cells from representative MF and SS tumors at BL, colored by cell type.

(C) Percent cell types in tumors at BL ordered by percent immune cells.

(D) %PD-1^+ of tumor-present T cell populations at BL.

(E) Differential immune features at BL colored by disease status or response. Ellipses indicate 95th percentile CI assuming a multivariate t-distribution.

(F) Sum or $\log_2$ ratio of features in (E) by response or disease status. Crossbar indicates mean. Wilcoxon rank sum.

(G) *cxcl13* expression by tumor-present neoplastic T cells from CTCL subjects. Diamond indicates mean.

(H) Bulk expression of *cxcl13* tumor mRNA and CXCL13 serum protein by time point and disease status. Blue triangle indicates MF subject with >10% neoplastic T cells out of PBMCs.

(I) *Z* scores of differential features at BL in which the leukemic MF subject more closely resembles SS subjects than MF subjects (Δ Mahalanobis distance > 0.3).

Systemic and local immune features differentiate MF and SS patients before and after anti-PD-1 therapy

MF and SS patients are both subtypes of CTCL but there are differences in their clinical manifestations, most notably the high frequency of circulating neoplastic T cells in SS patients, a defining criteria for SS diagnosis (Figure 1C).[45] We applied our immune modules model approach to discriminate MF and SS patients at BL and discovered 11 differential modules comprised of 25 features (Figures 6A, S7A, and S7B). We also applied our statistical approach to confirm and identify additional differential features (Figure S7C). As expected, the abundance of neoplastic T cells was a strong predictor of disease status. There was also significant enrichment for myeloid cells in the non-neoplastic PBMCs of SS patients (Figure 1E), but we did not observe a difference in the abundance of macrophages in the tumors of MF and SS patients (Figure S7D). There were, however, significantly more immune cells in the tumors of MF patients (Figure S7E), which was readily apparent in tumor cell maps (Figure 6B). Ordering patients by the abundance of tumor-infiltrating immune cells perfectly stratified MF and SS patients (Figure 6C).

The model identified greater PD-1 expression in SS patients across several T cell populations, including $CD8^+CLA^+CD39^+$ CM and EM cells (Figure 6A). In the tumor, we also found greater PD-1 expression in $CD4^+$, $CD8^+$, neoplastic, and Tregs in SS

tumors (Figure S7F). Unsupervised clustering based on PD-1 expression on tumor-present T cells perfectly stratified SS and MF patients (Figure 6D), but clustering based on HLA-DR expression failed to stratify SS and MF patients (Figure S7G). HLA-DR expression was not significantly different in tumor T cells between MF and SS patients (data not shown).

The proportion of PD-1$^+$ of CLA$^+$CD39$^+$ Treg EM cells was significantly higher in non-responders as well as in SS patients, and we found evidence of peripheral CD8$^+$ T cell activation differentiating both therapeutic response and disease status (Figures 5F and 6A). We visualized %PD-1$^+$ of CLA$^+$CD39$^+$ Treg EM cells and %HLA-DR$^+$ of CD8$^+$CLA$^+$CD39$^+$ EM cells at BL and colored plots by response and disease status (Figure 6E). Summing the features perfectly stratified MF and SS patients, while the ratio of the features significantly separated responders and non-responders (Figure 6F), highlighting the relevance of these cell populations.

An immune module containing serum PD-1 and CXCL13 was highly enriched in SS patients (Figure 6A). As we observed substantial expression of PD-1 on neoplastic T cells (Figures 2F–2H), we asked if CXCL13 was also expressed by neoplastic T cells. Utilizing a public scRNA-seq dataset,[44] we observed substantial expression of *cxcl13* in an SS patient's neoplastic T cells, but not in neoplastic T cells from patients with other CTCL subtypes (Figure 6G). Likewise, there was significantly greater *cxcl13* in SS tumors at BL, despite the greater immune infiltrate observed in MF patients (Figure 6H). This trend continued at C2 but did not reach statistical significance, while serum CXCL13 remained significantly higher in SS patients at both BL and C2. Interestingly, one MF patient who presented with abundant circulating neoplastic T cells (Figure 1C) also had elevated levels of serum CXCL13, but not tumor *cxcl13* (Figure 6H). This patient had elevated proportions of CD4$^+$CLA$^+$CD39$^+$ Trcm cells, characteristic of SS patients (Figure S1M), as well as the highest proportion of CD8$^+$CLA$^+$CD39$^+$ Trcm cells across both studies (Figure S7H). We utilized this leukemic MF patient to identify features differentiating MF and SS that were likely due to leukemic presentation rather than other aspects of the diseases. We compiled differential features selected by our immune module and statistical approaches, calculated the Mahalanobis distance between the leukemic MF patient and the MF and SS distributions, and visualized features in which the leukemic MF patient more closely resembled SS patients (Figure 6I). As expected, the higher levels of serum CXCL13 and PD-1 observed in SS patients were also found in this leukemic MF patient and were therefore likely due to the presence of circulating neoplastic T cells. Strikingly, we found elevated levels of serum CCL13, a chemoattractant enriched in chronic inflammatory disease,[46] serum IL-10, an immunoregulatory cytokine, and serum IL-16, a CD4$^+$ T cell chemoattractant and growth factor, were associated with leukemic presentation.

Phosphorylated ribosomal protein S6 (pS6), a proxy for translational activity,[47] was significantly lower in several CD4$^+$ and CD8$^+$ T cell populations in SS patients (Figures 6A and S7C). We also observed notably lower pS6 expression in total CD4$^+$ and CD8$^+$ T cells in SS compared with MF, and in CTCL patients compared with HCs (Figure S7I).

We also applied our immune module model and statistical approach to identifying differential features at C2 (Figures S7J–S7L). Several of the same features elevated in SS patients emerged again, including neoplastic T cell abundance and CD8$^+$CLA$^+$CD39$^+$ EM and CM T cell activation markers. Interestingly, several genes expressed by APCs in the tumor mRNA were significantly higher in SS patients, including *mrc1*, *axl*, *cd163*, *fcgr2b*, *marco*, *il18*, *siglec1*, *pecam1*, *csf1r*, and *b2m*. Applying our immune module model and statistical approaches to the change in features between BL and C2 revealed more substantial changes in the mRNA profile of SS tumors as compared with MF patients (Figures S7M–S7O).

To visualize the myriad differences in immune response kinetics between MF and SS patients, we compiled all differential features and visualized their mean *Z* scores at BL and C2, stratified by disease status, organized by feature kinetics, and ordered by BL effect size differences (Figure S8). Strikingly, there was a substantial increase over time (mean *Z* score difference > 0.5) in MF patients in only 2 differential features compared with 32 differential features in SS patients, including several APC genes.

In summary, we identified significant differences between SS and MF patients in immune composition and state in the tumor and periphery, defined features associated with leukemic presentation, and described the differential immune kinetics induced by anti-PD-1 therapy in these two subtypes of CTCL.

DISCUSSION

Here, we applied a systems immunology approach to characterize the endogenous and neoplastic immune responses to two immunotherapy regimens in patients with advanced CTCL. As the cancer cells in CTCLs are T cells, and T cells are the target of anti-PD-1 therapy, we deeply profiled the neoplastic T cell response to immunotherapy. We observed high PD-1 expression by neoplastic T cells but no signs of cellular activation or phenotypic shifts induced post-therapy. It is, however, possible that unobserved features and/or pathways were altered in neoplastic T cells after immunotherapy. A recent case study found an MF patient experienced neoplastic T cell activation and disease hyperprogression after CS1003 (anti-PD-1) therapy.[48] The mechanism for this neoplastic activation is unknown, but the authors speculated that PRKCQ amplification may have contributed. We observed no evidence of hyperprogression or neoplastic T cell activation in any of our patients administered with Pembrolizumab (anti-PD-1, n = 39), and hyperprogression was also not observed in CTCL patients receiving Tislelizumab (anti-PD-1, n = 11),[49] Atezolizumab (anti-PD-L1, n = 17),[50] or Durvalumab (anti-PD-L1, n = 23).[51] Our findings and these studies suggest that neoplastic activation and hyperprogression is uncommon in CTCLs following disruption of the PD-1 axis. Given that PRKCQ amplification has been observed in 20%–30% of CTCL patients,[52,53] it is unlikely that this somatic mutation alone is sufficient to potentiate neoplastic T cell activation. We observed high expression of CLA, CCR4, and CD39 in both neoplastic T cells and various reactive T cell subsets, so targeting these antigens may impair the endogenous response. A recent anti-CCR4 clinical trial resulted in an ORR of 28%,[3,54]

but the systemic effects on the endogenous T cell response and its potential for combination immunotherapy are unknown.

Quantifying $CD4^+$ neoplastic T cells as $CD95^-$ ($CD39^+$ or $KIR3DL2^+$) was more accurate than the current clinical $CD26^-$ gate.[1] The low false negative rate is attractive for early detection of leukemic involvement to inform diagnosis and treatment options,[3] but TCRseq would likely be more sensitive for quantifying rare neoplastic T cells once the neoplastic TCR is identified.[55,56] Some neoplastic T cells expressed the transcription factor, FOXP3, but did not adopt a Treg phenotype, although the immunoregulatory potential of neoplastic T cell subsets could be further explored through cytokine profiling and co-culture assays.[57,58]

Combination therapy showed no improvement in ORR over monotherapy. IFN-γ therapy induced muted and inconsistent induction of cellular activation, IFN-γ response genes, and PD-1 ligands. We did not observe this tepid response to IFN-γ in HCs, which had lower BL activation of monocytes than CTCL patients, providing greater opportunity for activation. Chronic exposure to endogenous IFN-γ may have rendered IFN-γ response pathways less sensitive to therapeutic IFN-γ in CTCL patients, consistent with negative feedback regulatory control of interferons.[59] The timing and duration of interferon treatment are also important considerations.[60]

After anti-PD-1 therapy, we observed increased abundance of T cells co-expressing CLA, a molecule facilitating skin homing,[12] and CD39, an ectoenzyme upregulated on tumor-specific T cells.[14,61–63] CLA^+CD39^+ T cells expressed activation molecules, were not specific to CMV peptide, and were associated with both therapeutic response and leukemic presentation. A recent study found circulating CLA^+CD39^+ T cells were associated with progression-free survival and therapeutic response to anti-PD-1 therapy in Merkel cell carcinoma, another skin cancer.[64] CLA^+CD39^+ cells were enriched for specificity to tumor-related antigens and expressed high levels of stemness and exhaustion genes, including *il7r*, *tcf7*, *tox*, and *tigit*. The observations from Ryu et al. and the current study suggest that CLA^+CD39^+ T cells may represent recirculating TILs in patients with skin cancers. These cells could provide a window into the immune state of the tumor without the requirement of a tumor biopsy. In non-responders, we observed increased activation of CLA^+CD39^+ Treg EM and CLA^+CD39^+ Tfr cells. Others have made similar observations about Treg activation in non-responders' tumors,[65,66] but we discovered these features in blood, even in patients lacking leukemic involvement. In SS patients, several CLA^+CD39^+ T cell populations displayed signs of greater activation, which may indicate that these cells are directly engaging neoplastic T cells in circulation.

We introduced immune modules to identify differential features stratifying therapeutic response, disease status, and immunotherapy regiment. The approach is akin to using weighted gene correlation network analysis[67] for integration and decorrelation of multi-omic data and a random forest classifier for discovery of biologically relevant discriminatory features.[68] Classifier performance can suffer with many correlated input variables,[69] so our approach reduces dimensionality through clustering of correlated features, which also enables exploring clustered features for biological co-regulation. This flexible approach is not limited to CTCL, immunotherapy, or even just human data, but could be applied for unbiased discovery of biomarkers or immunological insights between any two groups of samples, using any available quantitative measurements. Our approach represents a compromise between the competing goals of enabling biological discoveries while adhering to rigorous statistical practices to ensure reproducibility. Future methods should further explore integration of immune features into interpretable networks so we can derive knowledge from the intersection of features.[70]

In conclusion, our comprehensive profiling of CTCL patients revealed prominent features of the endogenous and neoplastic responses to immunotherapies and provides a framework for multi-omic profiling of clinical samples. Insights into the mechanisms of action and *in vivo* effects of immunotherapy in humans enables identification of biomarkers for patient selection, generates hypotheses about underlying biological mechanisms, and informs therapeutic target selection for our preclinical models.[71] A deeper understanding of the human immune system is vital for the progression of discovery-based science and its translation into healthier patients.

Limitations of the study

Neoplastic T cells were annotated based on their unique high-dimensional profiles, but without clonotype information we were only able to establish their identity based on proxies such as their expression patterns, their enrichment in SS patients, and the high correlation between neoplastic T cell quantitation by mass cytometry and bulk TCRseq. As such, it is possible that some $CD4^+$ T cells are annotated incorrectly. Our mass cytometry, tumor transcriptomics, and serum proteomics data represent targeted measurement of pre-selected analytes, so unobserved features may also play an important role in CTCL biology and the immunotherapy response. Results were based on patients from two clinical trials, so not all findings may generalize to other cohorts. Furthermore, the tumor reactivity of CLA^+CD39^+ T cells was not experimentally validated.

STAR★METHODS

Detailed methods are provided in the online version of this paper and include the following:

- KEY RESOURCES TABLE
- RESOURCE AVAILABILITY
  - Lead contact
  - Materials availability
  - Data and code availability
- EXPERIMENTAL MODEL AND STUDY PARTICIPANT DETAILS
  - Clinical cohorts
- METHOD DETAILS
  - Isolation of mononuclear cells from whole blood by density gradient separation
  - Serum and plasma processing
  - Biopsy sample collection and processing
  - Clinical flow cytometery quantitation of circulating neoplastic T cells
  - Bulk transcriptomics
  - Serum proteomics
  - TCR sequencing of peripheral blood
  - TCR sequencing of tumor biopsies
  - Antibody conjugation and cryopreservation

- Peptide-MHC tetramer generation and cryopreservation
- Mass cytometry workflow
- QUANTIFICATION AND STATISTICAL ANALYSES
  - Data preprocessing
  - Cell clustering
  - TCR analysis
  - Dimensionality reduction
  - Immune module generation
  - Classifier models
  - Statistical analyses

SUPPLEMENTAL INFORMATION

Supplemental information can be found online at https://doi.org/10.1016/j.xcrm.2024.101527.

ACKNOWLEDGMENTS

We thank Christian Schürch, Magdalena Matusiak, Peter Pickkers, Matthijs Kox, Sergei Koralov, and Anthony Cheng for sharing data and Heeju Ryu for helpful discussions. D.R.G. was supported by a Fred Hutchinson Cancer Center Mahan Fellowship, a Cancer Research Institute Irvington Postdoctoral Fellowship, and an American Society of Hematology Fellow Scholar Award. M.S.K. was supported by the National Institutes of Health, National Cancer Institute grant K08 CA207882 and an American Cancer Society-Stanford Cancer Institute Pilot Grant Award. This work was supported in part by the following grants: National Institutes of Health (R01 CA264646 to E.W.N), Fred Hutchinson Cancer Center New Development funds (to E.W.N.), The Andy Hill Endowment Distinguished Researcher CARE fund (to E.W.N), 1U01CA154967 and Cancer Center Support Grant P30 CA015704 from the National Cancer Institute, and Merck Sharp & Dohme Corp. MISP grant 57583 and funds from Horizon Therapeutics. We are deeply indebted to the patients and their families and to staff at all clinical sites.

AUTHOR CONTRIBUTIONS

Conceptualization, D.R.G., S.P.F., M.S.K., and E.W.N.; methodology, D.R.G.; software, D.R.G. and K.M.-B.; formal analysis, D.R.G., K.M.-B., A.N.B.T., and M.S.K.; investigation, D.R.G., N.R., K.R.P., G.D., A.N.B.T., L.I., Y.H.K., S.P.F., and M.S.K.; data curation, D.R.G., N.R., and A.K.W.; writing – original draft, D.R.G.; writing – review & editing, D.R.G., K.M.-B., K.R.P., S.P.F., M.S.K., and E.W.N.; visualization, D.R.G. and K.M.-B.; resources, N.R., Y.H.K., S.P.F., M.S.K., and E.W.N.; project administration, A.K.W., S.P.F., and E.W.N.; supervision, S.P.F., M.S.K., and E.W.N.; funding acquisition, S.P.F. and E.W.N.

DECLARATION OF INTERESTS

E.W.N. is a co-founder, advisor, and shareholder of ImmunoScape and is an advisor for Neogene Therapeutics and NanoString Technologies.

Received: September 14, 2023
Revised: February 17, 2024
Accepted: April 3, 2024
Published: April 25, 2024

REFERENCES

1. Pulitzer, M.P., Horna, P., and Almeida, J. (2021). Sézary syndrome and mycosis fungoides: An overview, including the role of immunophenotyping. Cytometry B Clin. Cytom. *100*, 132–138. https://doi.org/10.1002/cyto.b.21888.
2. Scarisbrick, J.J., Kim, Y.H., Whittaker, S.J., Wood, G.S., Vermeer, M.H., Prince, H.M., and Quaglino, P. (2014). Prognostic factors, prognostic indices and staging in mycosis fungoides and Sézary syndrome: where are we now? Br. J. Dermatol. *170*, 1226–1236. https://doi.org/10.1111/bjd.12909.
3. Khodadoust, M.S., Mou, E., and Kim, Y.H. (2023). Integrating novel agents into the treatment of advanced mycosis fungoides and Sézary syndrome. Blood *141*, 695–703. https://doi.org/10.1182/blood.2020008241.
4. Gong, J., Chehrazi-Raffle, A., Reddi, S., and Salgia, R. (2018). Development of PD-1 and PD-L1 inhibitors as a form of cancer immunotherapy: a comprehensive review of registration trials and future considerations. J. Immunother. Cancer *6*, 8. https://doi.org/10.1186/s40425-018-0316-z.
5. Khodadoust, M.S., Rook, A.H., Porcu, P., Foss, F., Moskowitz, A.J., Shustov, A., Shanbhag, S., Sokol, L., Fling, S.P., Ramchurren, N., et al. (2020). Pembrolizumab in Relapsed and Refractory Mycosis Fungoides and Sézary Syndrome: A Multicenter Phase II Study. J. Clin. Orthod. *38*, 20–28. https://doi.org/10.1200/JCO.19.01056.
6. Bachy, E., Savage, K.J., Huang, H., Kwong, Y.L., Gritti, G., Zhang, Q., Liberati, A.M., Cao, J., Yang, H., Hao, S., et al. (2023). Treating relapsed/refractory mature T- and NK-cell neoplasms with tislelizumab: a multicenter open label phase 2 study. Blood Adv. *7*, 4435–4447. https://doi.org/10.1182/bloodadvances.2022009575.
7. Ni, L., and Lu, J. (2018). Interferon gamma in cancer immunotherapy. Cancer Med. *7*, 4509–4516. https://doi.org/10.1002/cam4.1700.
8. Kaplan, E.H., Rosen, S.T., Norris, D.B., Roenigk, H.H., Jr., Saks, S.R., and Bunn, P.A., Jr. (1990). Phase II Study of Recombinant Human Interferon Gamma for Treatment of Cutaneous T-Cell Lymphoma. J. Natl. Cancer Inst. *82*, 208–212. https://doi.org/10.1093/jnci/82.3.208.
9. Khodadoust, M.S. (2023). Testing the Combination of Two Experimental Drugs MK-3475 (Pembrolizumab) and Interferon-gamma for the Treatment of Mycosis Fungoides and Sézary Syndrome and Advanced Synovial Sarcoma. ClinicalTrials.gov identifier: NCT03063632. Updated October 25 2023. https://clinicaltrials.gov/study/NCT03063632.
10. Phillips, D., Matusiak, M., Gutierrez, B.R., Bhate, S.S., Barlow, G.L., Jiang, S., Demeter, J., Smythe, K.S., Pierce, R.H., Fling, S.P., et al. (2021). Immune cell topography predicts response to PD-1 blockade in cutaneous T cell lymphoma. Nat. Commun. *12*, 6726. https://doi.org/10.1038/s41467-021-26974-6.
11. Britanova, O.V., Putintseva, E.V., Shugay, M., Merzlyak, E.M., Turchaninova, M.A., Staroverov, D.B., Bolotin, D.A., Lukyanov, S., Bogdanova, E.A., Mamedov, I.Z., et al. (2014). Age-Related Decrease in TCR Repertoire Diversity Measured with Deep and Normalized Sequence Profiling. J. Immunol. *192*, 2689–2698. https://doi.org/10.4049/jimmunol.1302064.
12. Fuhlbrigge, R.C., Kieffer, J.D., Armerding, D., and Kupper, T.S. (1997). Cutaneous lymphocyte antigen is a specialized form of PSGL-1 expressed on skin-homing T cells. Nature *389*, 978–981. https://doi.org/10.1038/40166.
13. Duhen, T., Duhen, R., Montler, R., Moses, J., Moudgil, T., de Miranda, N.F., Goodall, C.P., Blair, T.C., Fox, B.A., McDermott, J.E., et al. (2018). Co-expression of CD39 and CD103 identifies tumor-reactive CD8 T cells in human solid tumors. Nat. Commun. *9*, 2724. https://doi.org/10.1038/s41467-018-05072-0.
14. Simoni, Y., Becht, E., Fehlings, M., Loh, C.Y., Koo, S.-L., Teng, K.W.W., Yeong, J.P.S., Nahar, R., Zhang, T., Kared, H., et al. (2018). Bystander CD8 + T cells are abundant and phenotypically distinct in human tumour infiltrates. Nature *557*, 575–579. https://doi.org/10.1038/s41586-018-0130-2.
15. Becht, E., McInnes, L., Healy, J., Dutertre, C.-A., Kwok, I.W.H., Ng, L.G., Ginhoux, F., and Newell, E.W. (2018). Dimensionality reduction for visualizing single-cell data using UMAP. Nat. Biotechnol. *37*, 38–44. https://doi.org/10.1038/nbt.4314.
16. Buus, T.B., Willerslev-Olsen, A., Fredholm, S., Blümel, E., Nastasi, C., Gluud, M., Hu, T., Lindahl, L.M., Iversen, L., Fogh, H., et al. (2018). Single-cell heterogeneity in Sézary syndrome. Blood Adv. *2*, 2115–2126. https://doi.org/10.1182/bloodadvances.2018022608.

CellPress
OPEN ACCESS

17. Roelens, M., Delord, M., Ram-Wolff, C., Marie-Cardine, A., Alberdi, A., Maki, G., Homyrda, L., Bensussan, A., Bagot, M., Toubert, A., and Moins-Teisserenc, H. (2017). Circulating and skin-derived Sézary cells: clonal but with phenotypic plasticity. Blood *130*, 1468–1471. https://doi.org/10.1182/blood-2017-03-772996.

18. Bahler, D.W., Hartung, L., Hill, S., Bowen, G.M., and Vonderheid, E.C. (2008). CD158k/KIR3DL2 is a useful marker for identifying neoplastic T-cells in Sézary syndrome by flow cytometry. Cytometry B Clin. Cytometry *74B*, 156–162. https://doi.org/10.1002/cyto.b.20395.

19. Vergnolle, I., Douat-Beyries, C., Boulinguez, S., Rieu, J.-B., Vial, J.P., Baracou, R., Boudot, S., Cazeneuve, A., Chaugne, S., Durand, M., et al. (2022). CD158k and PD-1 expressions define heterogeneous subtypes of Sezary syndrome. Blood Adv. *6*, 1813–1825. https://doi.org/10.1182/bloodadvances.2021005147.

20. Bensussan, A., Janela, B., Thonnart, N., Bagot, M., Musette, P., Ginhoux, F., and Marie-Cardine, A. (2019). Identification of CD39 as a Marker for the Circulating Malignant T-Cell Clone of Sézary Syndrome Patients. J. Invest. Dermatol. *139*, 725–728. https://doi.org/10.1016/j.jid.2018.09.026.

21. Olsen, E.A., Whittaker, S., Kim, Y.H., Duvic, M., Prince, H.M., Lessin, S.R., Wood, G.S., Willemze, R., Demierre, M.-F., Pimpinelli, N., et al. (2011). Clinical End Points and Response Criteria in Mycosis Fungoides and Sézary Syndrome: A Consensus Statement of the International Society for Cutaneous Lymphomas, the United States Cutaneous Lymphoma Consortium, and the Cutaneous Lymphoma Task Force of the European Organisation for Research and Treatment of Cancer. J. Clin. Orthod. *29*, 2598–2607. https://doi.org/10.1200/JCO.2010.32.0630.

22. Willemze, R., Cerroni, L., Kempf, W., Berti, E., Facchetti, F., Swerdlow, S.H., and Jaffe, E.S. (2019). The 2018 update of the WHO-EORTC classification for primary cutaneous lymphomas. Blood *133*, 1703–1714. https://doi.org/10.1182/blood-2018-11-881268.

23. Tsai, A.G., Glass, D.R., Juntilla, M., Hartmann, F.J., Oak, J.S., Fernandez-Pol, S., Ohgami, R.S., and Bendall, S.C. (2020). Multiplexed single-cell morphometry for hematopathology diagnostics. Nat. Med. *26*, 408–417. https://doi.org/10.1038/s41591-020-0783-x.

24. Amouzgar, M., Glass, D.R., Baskar, R., Averbukh, I., Kimmey, S.C., Tsai, A.G., Hartmann, F.J., and Bendall, S.C. (2022). Supervised dimensionality reduction for exploration of single-cell data by HSS-LDA. Patterns *3*, 100536. https://doi.org/10.1016/j.patter.2022.100536.

25. Becht, E., Simoni, Y., Coustan-Smith, E., Evrard, M., Cheng, Y., Ng, L.G., Campana, D., and Newell, E.W. (2019). Reverse-engineering flow-cytometry gating strategies for phenotypic labelling and high-performance cell sorting. Bioinformatics *35*, 301–308. https://doi.org/10.1093/bioinformatics/bty491.

26. Rudensky, A.Y. (2011). Regulatory T Cells and Foxp3. Immunol. Rev. *241*, 260–268. https://doi.org/10.1111/j.1600-065X.2011.01018.x.

27. Wang, J., Ioan-Facsinay, A., van der Voort, E.I.H., Huizinga, T.W.J., and Toes, R.E.M. (2007). Transient expression of FOXP3 in human activated nonregulatory CD4+ T cells. Eur. J. Immunol. *37*, 129–138. https://doi.org/10.1002/eji.200636435.

28. Hanahan, D., and Weinberg, R.A. (2011). Hallmarks of Cancer: The Next Generation. Cell *144*, 646–674. https://doi.org/10.1016/j.cell.2011.02.013.

29. Hartmann, F.J., Mrdjen, D., McCaffrey, E., Glass, D.R., Greenwald, N.F., Bharadwaj, A., Khair, Z., Verberk, S.G.S., Baranski, A., Baskar, R., et al. (2021). Single-cell metabolic profiling of human cytotoxic T cells. Nat. Biotechnol. *39*, 186–197. https://doi.org/10.1038/s41587-020-0651-8.

30. Thommen, D.S., Koelzer, V.H., Herzig, P., Roller, A., Trefny, M., Dimeloe, S., Kiialainen, A., Hanhart, J., Schill, C., Hess, C., et al. (2018). A transcriptionally and functionally distinct PD-1+ CD8+ T cell pool with predictive potential in non-small-cell lung cancer treated with PD-1 blockade. Nat. Med. *24*, 994–1004. https://doi.org/10.1038/s41591-018-0057-z.

31. Lee, Y.J., Kim, J.Y., Jeon, S.H., Nam, H., Jung, J.H., Jeon, M., Kim, E.-S., Bae, S.J., Ahn, J., Yoo, T.-K., et al. (2022). CD39+ tissue-resident memory CD8+ T cells with a clonal overlap across compartments mediate antitumor immunity in breast cancer. Sci. Immunol. *7*, eabn8390. https://doi.org/10.1126/sciimmunol.abn8390.

32. Huang, A.C., Postow, M.A., Orlowski, R.J., Mick, R., Bengsch, B., Manne, S., Xu, W., Harmon, S., Giles, J.R., Wenz, B., et al. (2017). T-cell invigoration to tumour burden ratio associated with anti-PD-1 response. Nature *545*, 60–65. https://doi.org/10.1038/nature22079.

33. Sethna, Z., Elhanati, Y., Callan, C.G., Jr., Walczak, A.M., and Mora, T. (2019). OLGA: fast computation of generation probabilities of B- and T-cell receptor amino acid sequences and motifs. Bioinformatics *35*, 2974–2981. https://doi.org/10.1093/bioinformatics/btz035.

34. Goncharov, M., Bagaev, D., Shcherbinin, D., Zvyagin, I., Bolotin, D., Thomas, P.G., Minervina, A.A., Pogorelyy, M.V., Ladell, K., McLaren, J.E., et al. (2022). VDJdb in the pandemic era: a compendium of T cell receptors specific for SARS-CoV-2. Nat. Methods *19*, 1017–1019. https://doi.org/10.1038/s41592-022-01578-0.

35. Mayer-Blackwell, K., Schattgen, S., Cohen-Lavi, L., Crawford, J.C., Souquette, A., Gaevert, J.A., Hertz, T., Thomas, P.G., Bradley, P., and Fiore-Gartland, A. (2021). TCR meta-clonotypes for biomarker discovery with tcrdist3 enabled identification of public, HLA-restricted clusters of SARS-CoV-2 TCRs. Elife *10*, e68605. https://doi.org/10.7554/eLife.68605.

36. Kraal, G., van der Laan, L.J., Elomaa, O., and Tryggvason, K. (2000). The macrophage receptor MARCO. Microbes Infect. *2*, 313–316. https://doi.org/10.1016/S1286-4579(00)00296-3.

37. Parra, M. (2015). Class IIa HDACs – new insights into their functions in physiology and pathology. FEBS J. *282*, 1736–1744. https://doi.org/10.1111/febs.13061.

38. Yoon, S., and Eom, G.H. (2016). HDAC and HDAC Inhibitor: From Cancer to Cardiovascular Diseases. Chonnam Med. J. *52*, 1–11. https://doi.org/10.4068/cmj.2016.52.1.1.

39. Urbich, C., Rössig, L., Kaluza, D., Potente, M., Boeckel, J.-N., Knau, A., Diehl, F., Geng, J.-G., Hofmann, W.-K., Zeiher, A.M., and Dimmeler, S. (2009). HDAC5 is a repressor of angiogenesis and determines the angiogenic gene expression pattern of endothelial cells. Blood *113*, 5669–5679. https://doi.org/10.1182/blood-2009-01-196485.

40. Leentjens, J., Kox, M., Koch, R.M., Preijers, F., Joosten, L.A.B., van der Hoeven, J.G., Netea, M.G., and Pickkers, P. (2012). Reversal of Immunoparalysis in Humans In Vivo. Am. J. Respir. Crit. Care Med. *186*, 838–845. https://doi.org/10.1164/rccm.201204-0645OC.

41. Yoshie, O., and Matsushima, K. (2015). CCR4 and its ligands: from bench to bedside. Int. Immunol. *27*, 11–20. https://doi.org/10.1093/intimm/dxu079.

42. Dambuza, I.M., and Brown, G.D. (2015). C-type lectins in immunity: recent developments. Curr. Opin. Immunol. *32*, 21–27. https://doi.org/10.1016/j.coi.2014.12.002.

43. Griffith, J.W., Sokol, C.L., and Luster, A.D. (2014). Chemokines and Chemokine Receptors: Positioning Cells for Host Defense and Immunity. Annu. Rev. Immunol. *32*, 659–702. https://doi.org/10.1146/annurev-immunol-032713-120145.

44. Gaydosik, A.M., Tabib, T., Geskin, L.J., Bayan, C.-A., Conway, J.F., Lafyatis, R., and Fuschiotti, P. (2019). Single-Cell Lymphocyte Heterogeneity in Advanced Cutaneous T-cell Lymphoma Skin Tumors. Clin. Cancer Res. *25*, 4443–4454. https://doi.org/10.1158/1078-0432.CCR-19-0148.

45. Hwang, S.T., Janik, J.E., Jaffe, E.S., and Wilson, W.H. (2008). Mycosis fungoides and Sézary syndrome. Lancet *371*, 945–957. https://doi.org/10.1016/S0140-6736(08)60420-1.

46. Mendez-Enriquez, E., and García-Zepeda, E.A. (2013). The multiple faces of CCL13 in immunity and inflammation. Inflammopharmacol *21*, 397–406. https://doi.org/10.1007/s10787-013-0177-5.

47. Kimmey, S.C., Borges, L., Baskar, R., and Bendall, S.C. (2019). Parallel analysis of tri-molecular biosynthesis with cell identity and function in single cells. Nat. Commun. *10*, 1185. https://doi.org/10.1038/s41467-019-09128-7.

48. Gao, Y., Hu, S., Li, R., Jin, S., Liu, F., Liu, X., Li, Y., Yan, Y., Liu, W., Gong, J., et al. (2023). Hyperprogression of cutaneous T cell lymphoma after anti–PD-1 treatment. JCI Insight *8*, e164793. https://doi.org/10.1172/jci.insight.164793.

49. Bachy, E., Savage, K.J., Huang, H., Kwong, Y.-L., Gritti, G., Zhang, Q., Liberati, A.M., Cao, J., Yang, H., Hao, S., et al. (2022). Tislelizumab, a PD-1 inhibitor for relapsed/refractory mature T/NK-cell neoplasms: Results from a phase 2 study. J. Clin. Orthod. *40*, 7552. https://doi.org/10.1200/JCO.2022.40.16_suppl.7552.

50. Stadler, R., Romero, P.O., Bagot, M., Quaglino, P., Guenova, E., Jonak, C., Papadavid, E., Stranzenbach, R., Sartori, D., Musoro, J.Z., et al. (2021). Phase II trial of atezolizumab (anti-PD-L1) in the treatment of stage IIb–IVB mycosis fungoides/Sézary syndrome patients relapsed/refractory after a previous systemic treatment (PARCT). Eur. J. Cancer *156*, S22–S23. https://doi.org/10.1016/S0959-8049(21)00668-7.

51. Querfeld, C., Chen, L., Wu, X., Han, Z., Su, C., Banez, M., Quach, J., Barnhizer, T., Crisan, L., Rosen, S.T., and Zain, J.M. (2023). Preliminary Analysis Demonstrates Durvalumab (Anti-PD-L1) & Lenalidomide Is Superior to Single-Agent Durvalumab (anti-PD-L1) in Refractory/Advanced Cutaneous T Cell Lymphoma in a Randomized Phase 2 Trial. Blood *142*, 3077. https://doi.org/10.1182/blood-2023-188053.

52. Woollard, W.J., Pullabhatla, V., Lorenc, A., Patel, V.M., Butler, R.M., Bayega, A., Begum, N., Bakr, F., Dedhia, K., Fisher, J., et al. (2016). Candidate driver genes involved in genome maintenance and DNA repair in Sézary syndrome. Blood *127*, 3387–3397. https://doi.org/10.1182/blood-2016-02-699843.

53. Choi, J., Goh, G., Walradt, T., Hong, B.S., Bunick, C.G., Chen, K., Bjornson, R.D., Maman, Y., Wang, T., Tordoff, J., et al. (2015). Genomic landscape of cutaneous T cell lymphoma. Nat. Genet. *47*, 1011–1019. https://doi.org/10.1038/ng.3356.

54. Kim, Y.H., Bagot, M., Pinter-Brown, L., Rook, A.H., Porcu, P., Horwitz, S.M., Whittaker, S., Tokura, Y., Vermeer, M., Zinzani, P.L., et al. (2018). Mogamulizumab versus vorinostat in previously treated cutaneous T-cell lymphoma (MAVORIC): an international, open-label, randomised, controlled phase 3 trial. Lancet Oncol. *19*, 1192–1204. https://doi.org/10.1016/S1470-2045(18)30379-6.

55. Kirsch, I.R., Watanabe, R., O'Malley, J.T., Williamson, D.W., Scott, L.-L., Elco, C.P., Teague, J.E., Gehad, A., Lowry, E.L., LeBoeuf, N.R., et al. (2015). TCR sequencing facilitates diagnosis and identifies mature T cells as the cell of origin in CTCL. Sci. Transl. Med. *7*, 308ra158. https://doi.org/10.1126/scitranslmed.aaa9122.

56. Weng, W.-K., Armstrong, R., Arai, S., Desmarais, C., Hoppe, R., and Kim, Y.H. (2013). Minimal Residual Disease Monitoring with High-Throughput Sequencing of T Cell Receptors in Cutaneous T Cell Lymphoma. Sci. Transl. Med. *5*, 214ra171. https://doi.org/10.1126/scitranslmed.3007420.

57. Glass, M.C., Glass, D.R., Oliveria, J.-P., Mbiribindi, B., Esquivel, C.O., Krams, S.M., Bendall, S.C., and Martinez, O.M. (2022). Human IL-10-producing B cells have diverse states that are induced from multiple B cell subsets. Cell Rep. *39*, 110728. https://doi.org/10.1016/j.celrep.2022.110728.

58. Cherukuri, A., Rothstein, D.M., Clark, B., Carter, C.R., Davison, A., Hernandez-Fuentes, M., Hewitt, E., Salama, A.D., and Baker, R.J. (2014). Immunologic Human Renal Allograft Injury Associates with an Altered IL-10/TNF-α Expression Ratio in Regulatory B Cells. J. Am. Soc. Nephrol. *25*, 1575–1585. https://doi.org/10.1681/ASN.2013080837.

59. Khabar, K.S.A., and Young, H.A. (2007). Post-transcriptional control of the interferon system. Biochimie *89*, 761–769. https://doi.org/10.1016/j.biochi.2007.02.008.

60. Zemek, R.M., Chin, W.L., Fear, V.S., Wylie, B., Casey, T.H., Forbes, C., Tilsed, C.M., Boon, L., Guo, B.B., Bosco, A., et al. (2022). Temporally restricted activation of IFNβ signaling underlies response to immune checkpoint therapy in mice. Nat. Commun. *13*, 4895. https://doi.org/10.1038/s41467-022-32567-8.

61. Caushi, J.X., Zhang, J., Ji, Z., Vaghasia, A., Zhang, B., Hsiue, E.H.-C., Mog, B.J., Hou, W., Justesen, S., Blosser, R., et al. (2021). Transcriptional programs of neoantigen-specific TIL in anti-PD-1-treated lung cancers. Nature *596*, 126–132. https://doi.org/10.1038/s41586-021-03752-4.

62. Zheng, C., Fass, J.N., Shih, Y.-P., Gunderson, A.J., Sanjuan Silva, N., Huang, H., Bernard, B.M., Rajamanickam, V., Slagel, J., Bifulco, C.B., et al. (2022). Transcriptomic profiles of neoantigen-reactive T cells in human gastrointestinal cancers. Cancer Cell *40*, 410–423.e7. https://doi.org/10.1016/j.ccell.2022.03.005.

63. Krishna, S., Lowery, F.J., Copeland, A.R., Bahadiroglu, E., Mukherjee, R., Jia, L., Anibal, J.T., Sachs, A., Adebola, S.O., Gurusamy, D., et al. (2020). Stem-like CD8 T cells mediate response of adoptive cell immunotherapy against human cancer. Science *370*, 1328–1334. https://doi.org/10.1126/science.abb9847.

64. Ryu, H., Bi, T.M., Pulliam, T.H., Sarkar, K., Church, C.D., Kumar, N., Mayer-Blackwell, K., Jani, S., Ramchurren, N., Hansen, U.K., et al. (2024). Merkel cell polyomavirus-specific and CD39+CLA+ CD8 T cells as blood-based predictive biomarkers for PD-1 blockade in Merkel cell carcinoma. Cell Rep. Med. *5*, 101390. https://doi.org/10.1016/j.xcrm.2023.101390.

65. Huang, A.C., Orlowski, R.J., Xu, X., Mick, R., George, S.M., Yan, P.K., Manne, S., Kraya, A.A., Wubbenhorst, B., Dorfman, L., et al. (2019). A single dose of neoadjuvant PD-1 blockade predicts clinical outcomes in resectable melanoma. Nat. Med. *25*, 454–461. https://doi.org/10.1038/s41591-019-0357-y.

66. Kumagai, S., Togashi, Y., Kamada, T., Sugiyama, E., Nishinakamura, H., Takeuchi, Y., Vitaly, K., Itahashi, K., Maeda, Y., Matsui, S., et al. (2020). The PD-1 expression balance between effector and regulatory T cells predicts the clinical efficacy of PD-1 blockade therapies. Nat. Immunol. *21*, 1346–1358. https://doi.org/10.1038/s41590-020-0769-3.

67. Langfelder, P., and Horvath, S. (2008). WGCNA: an R package for weighted correlation network analysis. BMC Bioinf. *9*, 559. https://doi.org/10.1186/1471-2105-9-559.

68. Risom, T., Glass, D.R., Averbukh, I., Liu, C.C., Baranski, A., Kagel, A., McCaffrey, E.F., Greenwald, N.F., Rivero-Gutiérrez, B., Strand, S.H., et al. (2022). Transition to invasive breast cancer is associated with progressive changes in the structure and composition of tumor stroma. Cell *185*, 299–310.e18. https://doi.org/10.1016/j.cell.2021.12.023.

69. Toloşi, L., and Lengauer, T. (2011). Classification with correlated features: unreliability of feature ranking and solutions. Bioinformatics *27*, 1986–1994. https://doi.org/10.1093/bioinformatics/btr300.

70. Keidar Haran, T., and Keren, L. (2022). From genes to modules, from cells to ecosystems. Mol. Syst. Biol. *18*, e10726. https://doi.org/10.15252/msb.202110726.

71. Pulendran, B., and Davis, M.M. (2020). The science and medicine of human immunology. Science *369*, eaay4014. https://doi.org/10.1126/science.aay4014.

72. Chabon, J.J., Hamilton, E.G., Kurtz, D.M., Esfahani, M.S., Moding, E.J., Stehr, H., Schroers-Martin, J., Nabet, B.Y., Chen, B., Chaudhuri, A.A., et al. (2020). Integrating genomic features for non-invasive early lung cancer detection. Nature *580*, 245–251. https://doi.org/10.1038/s41586-020-2140-0.

73. Hartmann, F.J., Simonds, E.F., Vivanco, N., Bruce, T., Borges, L., Nolan, G.P., Spitzer, M.H., and Bendall, S.C. (2019). Scalable Conjugation and Characterization of Immunoglobulins with Stable Mass Isotope Reporters for Single-Cell Mass Cytometry Analysis. In Mass Cytometry: Methods and Protocols Methods in Molecular Biology, H.M. McGuire and T.M. Ashhurst, eds. (Springer), pp. 55–81. https://doi.org/10.1007/978-1-4939-9454-0_5.

74. Hartmann, F.J., Simonds, E.F., and Bendall, S.C. (2018). A Universal Live Cell Barcoding-Platform for Multiplexed Human Single Cell Analysis. Sci. Rep. *8*, 10770. https://doi.org/10.1038/s41598-018-28791-2.

75. Simoni, Y., Fehlings, M., and Newell, E.W. (2019). Multiplex MHC Class I Tetramer Combined with Intranuclear Staining by Mass Cytometry. In

Mass Cytometry: Methods and Protocols Methods in Molecular Biology, H.M. McGuire and T.M. Ashhurst, eds. (Springer), pp. 147–158. https://doi.org/10.1007/978-1-4939-9454-0_11.

76. Finck, R., Simonds, E.F., Jager, A., Krishnaswamy, S., Sachs, K., Fantl, W., Pe'er, D., Nolan, G.P., and Bendall, S.C. (2013). Normalization of mass cytometry data with bead standards. Cytometry A. *83*, 483–494. https://doi.org/10.1002/cyto.a.22271.

77. Chevrier, S., Crowell, H.L., Zanotelli, V.R.T., Engler, S., Robinson, M.D., and Bodenmiller, B. (2018). Compensation of Signal Spillover in Suspension and Imaging Mass Cytometry. Cell Syst. *6*, 612–620.e5. https://doi.org/10.1016/j.cels.2018.02.010.

78. Robinson, M.L., Glass, D.R., Duran, V., Agudelo Rojas, O.L., Sanz, A.M., Consuegra, M., Sahoo, M.K., Hartmann, F.J., Bosse, M., Gelvez, R.M., et al. (2023). Magnitude and kinetics of the human immune cell response associated with severe dengue progression by single-cell proteomics. Sci. Adv. *9*, eade7702. https://doi.org/10.1126/sciadv.ade7702.

79. Talhouk, A., Kommoss, S., Mackenzie, R., Cheung, M., Leung, S., Chiu, D.S., Kalloger, S.E., Huntsman, D.G., Chen, S., Intermaggio, M., et al. (2016). Single-Patient Molecular Testing with NanoString nCounter Data Using a Reference-Based Strategy for Batch Effect Correction. PLoS One *11*, e0153844. https://doi.org/10.1371/journal.pone.0153844.

80. Bolotin, D.A., Poslavsky, S., Mitrophanov, I., Shugay, M., Mamedov, I.Z., Putintseva, E.V., and Chudakov, D.M. (2015). MiXCR: software for comprehensive adaptive immunity profiling. Nat. Methods *12*, 380–381. https://doi.org/10.1038/nmeth.3364.

81. Herrera, A., Cheng, A., Mimitou, E.P., Seffens, A., George, D., Bar-Natan, M., Heguy, A., Ruggles, K.V., Scher, J.U., Hymes, K., et al. (2021). Multimodal single-cell analysis of cutaneous T-cell lymphoma reveals distinct subclonal tissue-dependent signatures. Blood *138*, 1456–1464. https://doi.org/10.1182/blood.2020009346.

82. Lause, J., Berens, P., and Kobak, D. (2021). Analytic Pearson residuals for normalization of single-cell RNA-seq UMI data. Genome Biol. *22*, 258. https://doi.org/10.1186/s13059-021-02451-7.

83. Hao, Y., Stuart, T., Kowalski, M.H., Choudhary, S., Hoffman, P., Hartman, A., Srivastava, A., Molla, G., Madad, S., Fernandez-Granda, C., and Satija, R. (2024). Dictionary learning for integrative, multimodal and scalable single-cell analysis. Nat. Biotechnol. *42*, 293–304. https://doi.org/10.1038/s41587-023-01767-y.

84. Glass, D.R., Tsai, A.G., Oliveria, J.P., Hartmann, F.J., Kimmey, S.C., Calderon, A.A., Borges, L., Glass, M.C., Wagar, L.E., Davis, M.M., and Bendall, S.C. (2020). An Integrated Multi-omic Single-Cell Atlas of Human B Cell Identity. Immunity *53*, 217–232.e5. https://doi.org/10.1016/j.immuni.2020.06.013.

85. Van Gassen, S., Callebaut, B., Van Helden, M.J., Lambrecht, B.N., Demeester, P., Dhaene, T., and Saeys, Y. (2015). FlowSOM: Using self-organizing maps for visualization and interpretation of cytometry data. Cytometry A. *87*, 636–645. https://doi.org/10.1002/cyto.a.22625.

86. Dash, P., Fiore-Gartland, A.J., Hertz, T., Wang, G.C., Sharma, S., Souquette, A., Crawford, J.C., Clemens, E.B., Nguyen, T.H.O., Kedzierska, K., et al. (2017). Quantifiable predictive features define epitope-specific T cell receptor repertoires. Nature *547*, 89–93. https://doi.org/10.1038/nature22383.

87. Britanova, O.V., Shugay, M., Merzlyak, E.M., Staroverov, D.B., Putintseva, E.V., Turchaninova, M.A., Mamedov, I.Z., Pogorelyy, M.V., Bolotin, D.A., Izraelson, M., et al. (2016). Dynamics of Individual T Cell Repertoires: From Cord Blood to Centenarians. J. Immunol. *196*, 5005–5013. https://doi.org/10.4049/jimmunol.1600005.

88. Murtagh, F., and Legendre, P. (2014). Ward's Hierarchical Clustering Method: Clustering Criterion and Agglomerative Algorithm. J. Classif. *31*, 274–295. https://doi.org/10.1007/s00357-014-9161-z.

89. Rousseeuw, P.J. (1987). Silhouettes: A graphical aid to the interpretation and validation of cluster analysis. J. Comput. Appl. Math. *20*, 53–65. https://doi.org/10.1016/0377-0427(87)90125-7.

STAR★METHODS

KEY RESOURCES TABLE

REAGENT or RESOURCE	SOURCE	IDENTIFIER
Antibodies		
Anti-Human CD45 (HI30)-89Y	Standard Bio-tools	CAT#3089003B; RRID:AB_2938863
Anti-Human CD45 (HI30)-106CD	Standard Bio-tools	CAT#3106001B
Anti-Human CD45 (HI30)-110CD	Standard Bio-tools	CAT#3110001B
Anti-Human CD45 (HI30)-111CD	Standard Bio-tools	CAT#3111001B
Anti-Human CD45 (HI30)-114CD	Standard Bio-tools	CAT#3114001B
Anti-Human CD45 (HI30)-116CD	Standard Bio-tools	CAT#3116001B
Anti-pS6 [S235/S236] (N7-548)-175Lu	Standard Bio-tools	CAT#3175009A; RRID:AB_2811251
CD56 Antibody, anti-human (REA196)	Miltenyi Biotec	CAT#130-108-016; RRID:AB_2658728
Mouse Anti-Human IgG4 Fc-UNLB (HP6025)	SouthernBiotech	CAT#9200-01; RRID:AB_2796689
Anti-VDAC1/Porin + VDAC3 antibody [20B12AF2]	abcam	CAT#ab14734; RRID:AB_443084
Recombinant Anti-Hexokinase II antibody [EPR20839]	abcam	CAT#ab209847; RRID:AB_2904621
Anti-ATP5A antibody [15H4C4] - Mitochondrial Marker	abcam	CAT#ab14748; RRID:AB_301447
Anti-CPT1A antibody [8F6AE9]	abcam	CAT#ab128568; RRID:AB_11141632
Human KIR3DL2/CD158k Antibody (539304)	R&D Systems	CAT#MAB2878; RRID:AB_3086694
Lamin A/C Antibody (E−1)	Santa Cruz Biotechnology	CAT#sc-376248; RRID:AB_10991536
KLRG1 Monoclonal Antibody (13F12F2)	Invitrogen	CAT#16-9488-85; RRID:AB_2637116
Purified Mouse Anti-Human CD98 (UM7F8)	BD Pharmingen	CAT#556074; RRID:AB_396341
Pembrolizumab (anti-PD-1)	Selleckchem	CAT#A2005; RRID:AB_3076193
Purified anti-human CD8a (RPA-T8)	BioLegend	CAT#301002; RRID:AB_314120
Purified anti-human CD14 (M5E2)	BioLegend	CAT#301802; RRID:AB_314184
Purified anti-human CD57 (HNK-1)	BioLegend	CAT#359602; RRID:AB_2562403
Purified anti-human/mouse Cutaneous Lymphocyte Antigen (CLA) (HECA-452)	BioLegend	CAT#321302; RRID:AB_492894
Purified anti-human HLA-DR (L243)	BioLegend	CAT#307602; RRID:AB_314680
Purified anti-human CD185 (CXCR5) (J252D4)	BioLegend	CAT#356902; RRID:AB_2561810
Purified anti-human CD4 (OKT4)	BioLegend	CAT#317402; RRID:AB_571963
Purified anti-human CD45RA (HI100)	BioLegend	CAT#304102; RRID:AB_314406
Purified anti-human CD103 (Integrin αE) (Ber-ACT8)	BioLegend	CAT#350202; RRID:AB_10639864
Purified anti-human/mouse/rat CD278 (ICOS) (C398.4A)	BioLegend	CAT#313502; RRID:AB_416326
Purified anti-human CD45RO (UCHL1)	BioLegend	CAT#304202; RRID:AB_314418
Purified anti-human CD194 (CCR4) (L291H4)	BioLegend	CAT#359402; RRID:AB_2562364
Purified anti-human CD38 (HIT2)	BioLegend	CAT#303502; RRID:AB_314354
Purified anti-human CD127 (IL-7Rα) (QA18A44)	BioLegend	CAT#375702; RRID:AB_2888838
Purified anti-human CD161 (W18070C)	BioLegend	CAT#307502; RRID:AB_2860820
Purified anti-human CD26 (BA5b)	BioLegend	CAT#302702; RRID:AB_314286
Purified anti-human Ki-67 (Ki-67)	BioLegend	CAT#350502; RRID:AB_10662749
Purified anti-human TCR γ/δ (B1)	BioLegend	CAT#331202; RRID:AB_1089222
Purified anti-human CD183 (CXCR3) (G025H7)	BioLegend	CAT#353702; RRID:AB_10983073
Purified anti-human CD27 (M-T271)	BioLegend	CAT#356401; RRID:AB_2561786
Purified anti-human CD95 (Fas) (EOS9.1)	BioLegend	CAT#305702; RRID:AB_314554
Purified anti-human CD25 (BC96)	BioLegend	CAT#302602; RRID:AB_314272
Purified anti-human CD274 (B7-H1, PD-L1) (29E.2A3)	BioLegend	CAT#329702; RRID:AB_940372
Purified anti-human CD152 (CTLA-4)	BioLegend	CAT#369602; RRID:AB_2566610
Purified anti-human CD197 (CCR7) (G043H7)	BioLegend	CAT#353202; RRID:AB_10945157

(*Continued on next page*)

Continued		
REAGENT or RESOURCE	SOURCE	IDENTIFIER
Purified anti-human FOXP3 (206D)	BioLegend	CAT#320102; RRID:AB_430881
Purified anti-human CD39 (A1)	BioLegend	CAT#328202; RRID:AB_940438
Purified anti-human CD28 (CD28.2)	BioLegend	CAT#302902; RRID:AB_314304
Purified anti-human CD3 (UCHT1)	BioLegend	CAT#300402; RRID:AB_314056
Chemicals, peptides, and recombinant proteins		
Cell-ID^{TM} Intercalator-Rh	Standard Bio-tools	CAT#201103A
Cell-ID^{TM} Cisplatin-195Pt	Standard Bio-tools	CAT#201195
Human TruStain FcX^{TM} (Fc Receptor Blocking Solution)	BioLegend	CAT#422301
Viral peptides	Mimotopes	Custom
UV-cleavable MHC class I monomers	Fred Hutchinson Cancer Center	Custom
Streptavidin	Fred Hutchinson Cancer Center	Custom
Critical commercial assays		
Maxpar X8 Antibody Labeling Kit	Standard Bio-tools	CAT#201300
DNeasy Blood & Tissue Kit	Qiagen	CAT#69504
AllPrep DNA/RNA FFPE Kit	Qiagen	CAT#80234
KAPA HyperPlus Kit	Roche	CAT# 7962380001 – KK8510
Deposited data		
All original data	Data are deposited in Zenodo	Zenodo: https://doi.org/10.5281/zenodo.8213279
Software and algorithms		
CellEngine	CellCarta	N/A
R	R Foundation for Statistical Computing	Version 4.2.0
RStudio	Posit	Version 2023.09.0 + 463
Immune modules code	Code is deposited in Zenodo	Zenodo: https://doi.org/10.5281/zenodo.10846411

RESOURCE AVAILABILITY

Lead contact

Further information and requests for resources and reagents should be directed to and will be fulfilled by the lead contact, Evan W. Newell (enewell@fredhutch.org).

Materials availability

All unique and stable reagents generated in this study are available to the scientific community upon request and following execution of materials transfer agreement by contacting the lead contact.

Data and code availability

All data generated for this manuscript has been deposited at Zenodo: https://doi.org/10.5281/zenodo.8213279. Immune modules code has been deposited at Zenodo: https://doi.org/10.5281/zenodo.10846411. Any additional information required to reanalyze the data reported here is available upon request of the corresponding authors.

EXPERIMENTAL MODEL AND STUDY PARTICIPANT DETAILS

Clinical cohorts

All patients enrolled in this study provided written informed consent, and the analysis was performed according to the IRB approval numbers FHIRB0008268 and FHIRB0008588. CITN-10 (NCT02243579) was a multicenter, phase II clinical trial testing the efficacy of intravenous anti-PD-1 (Pembrolizumab) monotherapy in 24 patients with MF or SS, two common subtypes of CTCL, clinical stage IB to IV. CITN-13 Treatment Group 1 (NCT03063632) was a multicenter, phase II clinical trial testing the efficacy of subcutaneous IFN-γ and intravenous anti-PD-1 (Pembrolizumab) combination therapy in 16 patients with MF or SS, also clinical stage IB to IV. One patient from CITN-13 died before receiving treatment and was therefore not utilized for analysis or

calculation of ORR in the current study. All patients from both trials had relapsed, were refractory to, or had progressed after at least one standard systemic therapy. All patients received intravenous anti-PD-1 every three weeks and CITN-13 patients additionally received subcutaneous IFN-γ three times per week, including a one-week lead-in preceding the onset of anti-PD-1 therapy. Cryopreserved peripheral blood mononuclear cells (PBMCs), cryopreserved serum, and formalin-fixed paraffin-embedded (FFPE) tumor biopsy samples were available from longitudinal timepoints collected before, during, and after therapy. Clinical information, including therapeutic response and progression-free survival, was also available for every patient. The response rate was 38% for CITN-10 and 40% for CITN-13.

METHOD DETAILS

Isolation of mononuclear cells from whole blood by density gradient separation

Peripheral blood was obtained longitudinally from study participants. Whole blood was collected by venipuncture into heparinized tubes (BD) and shipped ambient overnight to the CITN Central Lab (CIML). Peripheral blood mononuclear cells (PBMCs) were isolated by density gradient separation. Briefly, fresh blood (<36 h post-draw) was diluted 1.5-fold in HBSS (w/o Ca or Mg) and PBMCs were purified by Ficoll-Hypaque density gradient centrifugation protocol. PBMCs were resuspended in freezing media (RPMI +10% DMSO +12.5% Human Serum Albumin) and frozen in aliquots of 5–20 million cells per cryovial and stored in temperature-monitored LiqN2 vapor phase freezers until later use. Prior to use, PBMCs were gradually thawed in a 37°C water bath and resuspended in prewarmed complete RPMI (RPMI +10% FBS +1% Penicillin/Streptomycin) and then centrifuged for 1,500 rpm for 7 min at room temperature. Cells were then re-suspended in complete RPMI and benzonase nuclease (1 μL benzonase/1 mL of media) for 10 min at 37°C to avoid cell clumping and quantified.

Serum and plasma processing

Whole blood was collected from study participants by venipuncture into Red Top tubes (serum) and heparinized tubes (plasma). Serum and plasma were processed at the local clinical labs within 3hrs of draw and isolated using standard ultracentrifugation procedures and stored at −80′C. Frozen vials were shipped in batch over-night on dry ice from clinical sites to the CIML for long term storage at-80′C.

Biopsy sample collection and processing

Pre-treatment and post-treatment skin biopsies were collected according to standard pathology surgical procedures. Biopsies were immediately fixed in formalin, sent ambient over-night to the CIML and immediately embedded in paraffin and stored at 4′C on desiccant until later use. FFPE sections/curls (10 and 20 μm each) were prepared by microtome from blocks of embedded tissue by Fred Hutch Experimental Histopathology for isolation of RNA for bulk transcriptomics.

Clinical flow cytometery quantitation of circulating neoplastic T cells

For the clinical trials, neoplastic T cells were defined as T cells which are either $CD4^{+}CD7^{-}$ or $CD4^{+}CD26^{-}$.[21] For an individual patient only one of the phenotypes was used. The protocols allowed the use of either gate since there is variation in which phenotype best represents an individual patient's neoplastic phenotype. This cytometry was performed on fresh PBMCs at the individual research sites at baseline and at specified post-treatment time points.

Bulk transcriptomics

Bulk transcriptomic gene expression analyses from biopsies was performed using the 770-gene Pan Cancer Immune Profiling Panel (Research Only) NanoString Technologies (Seattle, WA) on RNA extracted from FFPE samples. RNA was prepared from FFPE curls using Purigen Biosystems Ionic Purification System technology. RNA was quatified by Nanodrop and QC'd by Tapestation 4200. ∼100ng of total RNA was input into each hybridization. Each RNA sample was combined with 5′-fluorescently barcoded reporter probe, 3′-biotinylated capture probe, hybridization buffer and then hybridized at 65°C. Hybridized probe-RNA complexes were purified on the nCounter Prep Station using 'High Sensitivity' protocol, followed by digital counting on nCounter Digital Analyzer and raw data collected at the 280 field-of-view setting and analyzed using nCounter with Advanced Analysis module.

Serum proteomics

Olink Proximity Extension Assay (PEA) technology was used to assess circulating levels of proteins. For CITN-10, serum samples were analyzed using a total of fourteen human Olink Target 96 panels (Target 96 Immuno-Oncology; Cardiovascular; Neurology; Oncology; Inflammation; Biological Process Panels). For CITN-13, plasma samples were analyzed using four Olink Explore 384-plex panels.

TCR sequencing of peripheral blood

Bulk TCR sequencing was performed on CITN-13 PBMC samples. DNA was extracted from cryopreserved PBMCs (10^6 cells/sample) using the Qiagen DNAeasy blood and tissue kit according to the manufacturer's instruction and sequenced using the TCRbeta immunoSEQ Assay (Adaptive Biosciences, Seattle, WA).

TCR sequencing of tumor biopsies

DNA was extracted from FFPE skin tumor biopsies using Qiagen FFPE Allprep Kit (Qiagen). Barcoded DNA libraries were prepared using Kapa HyperPlus kit (Roche) as previously described.[72] Briefly, 10–75 ng of DNA were subjected to 20-min enzymatic fragmentation, end repair/A-tailing, adapter ligation, barcode-grafting PCR, and universal PCR. Library enrichment was performed by hybrid capture with a custom 2.3 Mb oligonucleotide panel (Twist Bioscience) that included full coverage of the T cell receptor gene loci (180 genomic regions) and 225 genes known to be recurrently mutated or of known biological significance in CTCL. Libraries were sequenced on an Illumina HiSeq4000 with 2x150bp paired-end reads. Median fragment length and median barcode-deduped sequencing depth were 293bp and 2450x, respectively.

Antibody conjugation and cryopreservation

Antibody conjugation was performed as previously described.[73] Briefly, metal isotope–labeled antibodies used in this study were conjugated using the MaxPar X8 antibody labeling kit per the manufacturer's instructions (Standard Biotools) or purchased from Standard Biotools preconjugated. Each conjugated antibody was quality checked and titrated to optimal staining concentration on healthy human PBMCs. Aliquots of the optimized surface antibody and CD45 live cell barcoding (LCB) cocktails[74] were cryopreserved in cell-staining media [CSM: PBS, 0.5% bovine serum albumin, and 0.02% sodium azide (Sigma-Aldrich], while optimized intracellular antibody cocktails were cryopreserved in FoxP3/transcription factor permeabilization buffer (eBiosciences) at -80°C.

Peptide-MHC tetramer generation and cryopreservation

Tetramer generation and conjugation was performed as previously described.[75] Briefly, a unique code was generated by conjugating streptavidin to two metal isotopes as above and each pMHC was associated with a specific metal combination (Table S4). For each pMHC, 5 μL peptide (1 mM) was added to 100 μL HLA monomer (100 μg/mL, diluted in PBS) and exposed to UV (365 nm) for 10 min for peptide exchange. For tetramerization, 5 μL of labeled streptavidin (50 μg/mL) was mixed with pMHC and incubated for 10 min at 25°C. This was repeated three additional times for a total addition of 20 μL. The solutions were incubated for an additional hour at 25°C and then combined into tetramer cocktails, aliquoted, and cryopreserved as above.

Mass cytometry workflow

Samples were processed in five batches of 16–18 samples, with balanced representation of samples by response, status, and timepoint. For each batch, an aliquot of a common cryopreserved PBMC sample from a healthy donor was added for batch correction. Up to two million cells from each sample were washed in CSM and stained with LCB, tetramers, and Pembrolizumab for 30 min at 25°C. Cells were washed with CSM, combined into a single tube, and incubated with FC block (FcX, BioLegend) for 10 min at 25°C. Cells were stained with surface antibodies for 30 min at 25°C and then resuspended in an additional 1 mL of 500 nM PdCl in PBS for 5 min. Cells were washed in CSM, then fixed and permeabilized according to manufacturer's instructions (eBioscience FoxP3/transcription factor permeabilization buffer). Cells were stained with intracellular antibodies for 30 min at 25°C and washed in CSM. Cells were incubated in 4mL intercalator solution [PBS, 1.6% paraformaldehyde, and 0.5 mM rhodium (Standard Biotools)] for 1 h, washed, and resuspended in 1 mL CSM before cryopreservation at -80°C. Cells were thawed and washed in Cell Acquisition Solution (CAS), filtered through a 35-mm nylon mesh cell strainer, and resuspended in CAS with 1 x EQ four-element calibration beads (Standard Biotools). Barcoded samples were acquired in several fcs files on a Helios mass cytometer (Standard Biotools).

QUANTIFICATION AND STATISTICAL ANALYSES

Data preprocessing

Mass cytometry

Fcs files were bead normalized[76] and compensated.[77] Within each batch, channels with remaining time-based drift were subject to quantile normalization after inverse hyperbolic sine (asinh) transform. Files were debarcoded with the *premessa* R package and uploaded to cellengine.com for viable singlet gating using DNA, center, event length, bead distance, and viability stain. Batch correction was then applied using a common control sample as previously described.[78] Fcs files were combined and individual protein channels were arcsinh-transformed with a cofactor of 5 and scaled to the 99.9th percentile. Individual tetramer^{+} gates were drawn by batch and class I HLA-type to identify antigen-specific CD8^{+} cells. CD4^{+} T cells were used as negative controls to set thresholds. Only antigens with >100 antigen-specific cells were included in downstream analyses.

Serum proteomics

Olink proteomics data with quality flags were filtered out. Any measurement below the limit of detection was replaced with limit of detection threshold. Any measurements with quality warnings were replaced with assay median. Each protein was shifted to have identical medians at baseline between the clinical trials to account for batch effect.

Bulk transcriptomics

Nanostring nCounter data with quality flags were filtered out. Data were $\log_2$ transformed and normalized to housekeeping genes.[79] Each gene was shifted to have identical medians at baseline between the clinical trials to account for batch effect.

TCR sequencing

V, D, J, and CDR3 calls were used as identified by Adaptive Biotechnology's bioinformatic pipeline for peripheral blood samples. MiXCR was used to process sequencing data from tumor biopsy samples.[80] Sequences with identical V genes, J genes, and CDR3 sequences from the same subject were grouped into clonal families. Non-productive CDR3s were filtered.

CODEX

The cell count matrix was used in analysis with original annotations and preprocessing steps from Phillips and colleagues.[10] Spots with <5% tumor cells were not included in analyses. For quantification of PD-1 and HLA-DR percent positivity, a per spot threshold was used to account for staining variation. For PD-1, the 95th percentile of PD-1 expression in stromal and vascular cells were used to set the threshold for positivity, while stroma and epithelium were used for HLA-DR expression.

scRNAseq

The cell count matrix was used in analysis with original annotations from Gaydosik and colleagues.[44] Gene counts were $\log_{10}$ transformed and scaled to each gene's maximum.

Single-cell multi-omics

The RNA cell count matrix and TCR clonotype matrix was used in analysis with original annotations from Herrera and colleagues.[81] TCR and Ig variable genes were removed from the RNA matrix and then data were transformed using SCTransform[82] from the Seurat R package.[83] PCA was performed and the first 26 PCs were selected by visual inspection of an ElbowPlot for downstream nearest neighbor analysis.

Cell clustering

A supervised hierarchical clustering approach was employed, as previously described.[78,84] Cells were overclustered by FlowSOM[85] using all markers as input and clusters were manually annotated as T cells or non-T cells based on expression of CD3. Neoplastic T cell clusters were annotated based on expression of KIR3DL2,[19] absence of CD8, absence of TCR$\gamma\delta$, and a high proportion of cells belong to SS subjects (>93%). Manual inspection of UMAPs derived from total subjects (Figure 1B) and individual subjects (Figure S1I, top row) demonstrated clear separation between neoplastic T cells and normal T cells in UMAP space. The same phenomenon was evident in published peripheral blood and tumor scRNAseq data of SS patients[81] (Figure S1I, middle row). In that study, single-cell TCRseq confirmed that neoplastic clonotypes were found exclusively in cells labeled as, and co-localizing with, neoplastic T cells (Figure S1I, bottom row). Non-T cells were reclustered using FlowSOM with all markers as input and clusters were manually annotated into NK cells, myeloid cells, B cells, and plasma cells (Figure S1H). Non-neoplastic T cells were reclustered using FlowSOM with all markers except CD14, CD38, CD98, HLA-DR, PD-1, Ki-67, CTLA-4, VDAC1, HK2, ATP5A, pS6, CPT1A, Lamin A/C. This facilitated unbiased quantification of these activation and metabolic markers across clusters for downstream analyses. Clusters were then manually annotated in coarse and granular cell populations based on marker expression (Figure S1M).

TCR analysis

Neoplastic TCRs were identified from the targeted TCR sequencing as the most abundant clone with at least 75 counts. Gini-Simpson diversity and Morista-Horn similarity were quantified using the *abdiv* R package. We computed TCR distances[86] using tcrdist3 software[35] on TCRβ receptors from clones of interest, defined as neoplastic clones, tumor-present clones, abundant clones (>0.01% of TCR templates), and significantly differentially abundant clones between any two timepoints ($q < 0.05$, false discovery rate (FDR)-corrected Fisher's exact test). Retaining all distances ≤ 12 TCR distance units (approximately 1–1.5 edit distance, and a highly similar TRBV gene), we visualized a similarity network using the Python package, *networkX*. We extracted the connected components within the sequence similarity network, and for each connected component, we sampled a background set of TCRs from umbilical cord blood[87] with the same TRBV- and TRBJ-gene usage to permit generation-background-subtracted CDR3 motifs, which emphasize conserved non-germline encoded residues.

Dimensionality reduction

UMAPs were generated using the *uwot* R package with n_neighbors = 15, min_dist = 0.4, and the PCA was generated using the *stats* R package. In Figure 1, the UMAP was comprised of an equal sampling of PBMCs from HC, MC, and SS subjects at baseline with all protein markers from the mass cytometry panel as input. In Figure 2, the UMAP was comprised of an equal sampling of neoplastic T cells from all subjects with leukemic presentation with all protein markers from the mass cytometry panel, except CD14, $\gamma\delta$TCR, CD8, Ki-67, and Lamin A/C as input. In Figure 2, The PCA was comprised of median marker expression of the displayed markers, by subject and indicated population. In Figure 4, the UMAP was comprised of an equal sampling of non-neoplastic T cells from all CTCL subjects at BL and C2 and all antigen-specific T cells with all protein markers from the mass cytometry panel, except CD14 and Lamin A/C.

Immune module generation

Immune features were calculated for BL, C2, and the difference between the two timepoints for all CTCL subjects. Immune features consisted of tumor transcriptomics expression levels, serum proteomics expression levels, and summary statistics of mass cytometry data. Summary statistics included cluster abundance, mean expression of metabolic markers by clusters, and percent positive expression of activation markers by clusters. If a cluster had less than 15 cells in a sample, only the cluster frequency was calculated

CellPress
OPEN ACCESS

for that sample to avoid subsampling error. Any missing values were replaced with the column mean. For each timepoint, Spearman correlation was calculated for all pairwise immune features across all CTCL subjects. The resulting matrix was transformed into a distance matrix with the following equation:

$$d_{ij} = 1 - \rho_{ij}^4$$

where d is the resulting distance matrix and ρ is the correlation matrix. Raising ρ to the fourth power removed the sign and increased the distance of features with only a moderate correlation.

The distance matrix was used as input to hierarchically cluster features into immune modules using the Ward method[88] across a large range of numbers of clusters. The optimal number of clusters was selected by choosing the clustering with the maximal silhouette score.[89] Features clustered together were summarized by performing principal component analysis on Z-scored features and calculating the first principal component.

Classifier models

For each patient stratification and timepoint, hyperparameters for a random forest classifier (*ranger* R package) were selected by minimizing out-of-bag error. The tuned model was rerun across ten random seeds to evaluate the stability of the model's error rate. To identify the most representative seed, the top ten predictive features were identified for each seed using Gini index, weighted by rank, and summed. The seed with top features most overlapping with this ranking was selected as the most representative and used for downstream analysis. The Gini index from this model was used to calculate variable importance and features above a threshold set for each model were further analyzed.

Statistical analyses

For unpaired comparison of distributions, Wilcoxon rank-sum test was used. For paired comparison of distributions, Wilcoxon signed rank test was used. For comparison of count data, Fisher's exact test was used. The Benjamini-Hochberg FDR correction was used for multiple hypothesis where indicated. In situations involving multiple comparisons, differences were considered statistically significant if $q < 0.1$ or ($p < 0.05$ and |effect size| > 1).

Cell Reports Medicine, Volume *5*

Supplemental information

Multi-omic profiling reveals the endogenous and neoplastic responses to immunotherapies in cutaneous T cell lymphoma

David R. Glass, Koshlan Mayer-Blackwell, Nirasha Ramchurren, K. Rachael Parks, George E. Duran, Anna K. Wright, Armando N. Bastidas Torres, Laura Islas, Youn H. Kim, Steven P. Fling, Michael S. Khodadoust, and Evan W. Newell

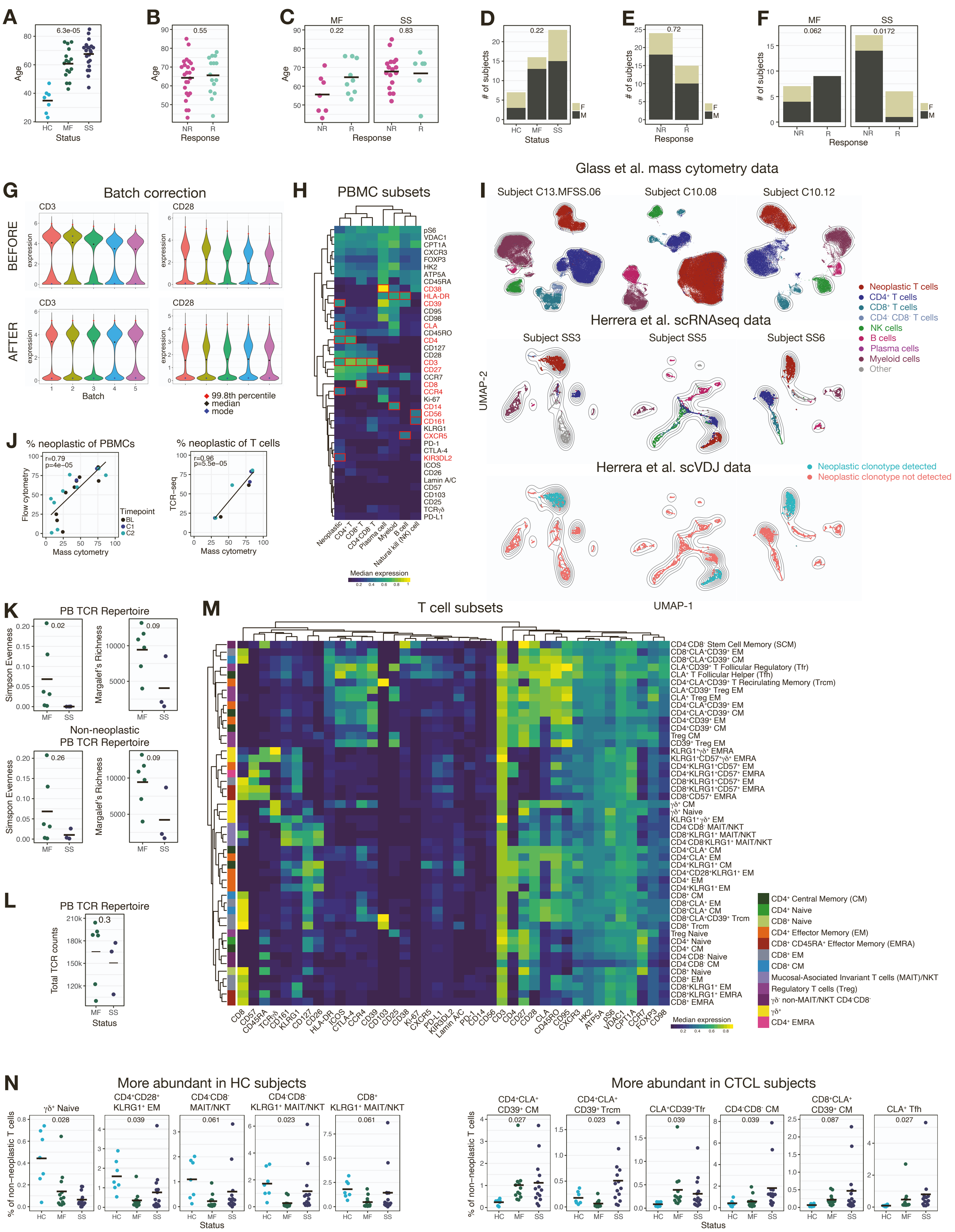

A
Age
6.3e-05
HC MF SS
Status
B
0.55
NR R
Response
C
MF SS
0.22 0.83
Response
D
# of subjects
0.22
F M
Status
E
0.72
Response
F
MF SS
0.062 0.0172
Response
G
Batch correction
CD3 CD28
BEFORE
AFTER
expression
Batch
99.8th percentile
median
mode
H
PBMC subsets
pS6 VDAC1 CPT1A CXCR3 FOXP3 HK2 ATP5A CD45RA CD38 HLA-DR CD39 CD95 CD98 CLA CD45RO CD4 CD127 CD28 CD3 CD27 CCR7 CD8 CCR4 Ki-67 CD14 CD56 CD161 KLRG1 CXCR5 PD-1 CTLA-4 KIR3DL2 ICOS CD26 Lamin A/C CD57 CD103 CD25 TCRγδ PD-L1
Neoplastic, CD4+ T, CD8+ T, CD4-CD8- T, Plasma cell, Myeloid, B cell, Natural kill (NK) cell
Median expression
Glass et al. mass cytometry data
I
Subject C13.MFSS.06
Subject C10.08
Subject C10.12
Herrera et al. scRNAseq data
Subject SS3
Subject SS5
Subject SS6
Herrera et al. scVDJ data
UMAP-2
UMAP-1
Neoplastic T cells
CD4+ T cells
CD8+ T cells
CD4- CD8- T cells
NK cells
B cells
Plasma cells
Myeloid cells
Other
Neoplastic clonotype detected
Neoplastic clonotype not detected
J
% neoplastic of PBMCs
r=0.79
p=4e-05
Flow cytometry
Mass cytometry
Timepoint
BL
C1
C2
% neoplastic of T cells
r=0.96
p=5.5e-05
TCR-seq
K
PB TCR Repertoire
Simpson Evenness
Margalef's Richness
0.02
0.09
Non-neoplastic PB TCR Repertoire
0.26
0.09
MF SS
L
PB TCR Repertoire
0.3
Total TCR counts
Status
M
T cell subsets
CD4-CD8- Stem Cell Memory (SCM)
CD8+CLA+CD39+ EM
CD8+CLA+CD39+ CM
CLA+CD39+ T Follicular Regulatory (Tfr)
CLA+ T Follicular Helper (Tfh)
CD4+CLA+CD39+ T Recirculating Memory (Trcm)
CLA+CD39+ Treg EM
CLA+ Treg EM
CD4+CLA+CD39+ EM
CD4+CLA+CD39+ CM
CD4+CD39+ EM
CD4+CD39+ CM
Treg CM
CD39+ Treg EM
KLRG1+γδ+ EMRA
KLRG1+CD57+γδ+ EMRA
CD4+KLRG1+CD57+ EM
CD4+KLRG1+CD57+ EMRA
CD8+KLRG1+CD57+ EM
CD8+KLRG1+CD57+ EMRA
CD8+CD57+ EMRA
γδ+ CM
γδ+ Naive
KLRG1+γδ+ EM
CD4-CD8- MAIT/NKT
CD8+KLRG1+ MAIT/NKT
CD4-CD8-KLRG1+ MAIT/NKT
CD4+CLA+ CM
CD4+CLA+ EM
CD4+KLRG1+ CM
CD4+CD28+KLRG1+ EM
CD4+ EM
CD4+KLRG1+ EM
CD8+ CM
CD8+CLA+ EM
CD8+CLA+ CM
CD8+CLA+CD39+ Trcm
CD8+ Trcm
Treg Naive
CD4+ Naive
CD4+ CM
CD4-CD8- Naive
CD4-CD8- CM
CD8+ Naive
CD8+ EM
CD8+KLRG1+ EM
CD8+KLRG1+ EMRA
CD8+ EMRA
CD8 CD57 CD45RA TCRγδ CD161 KLRG1 CD127 CD26 HLA-DR ICOS CTLA-4 CCR4 CD39 CD103 CD25 CD38 Ki-67 CXCR5 PD-L1 KIR3DL2 Lamin A/C PD-1 CD14 CD56 CD3 CD4 CD27 CD28 CLA CD45RO CD95 CXCR3 HK2 ATP5A pS6 VDAC1 CPT1A CCR7 FOXP3 CD98
Median expression
CD4+ Central Memory (CM)
CD4+ Naive
CD8+ Naive
CD4+ Effector Memory (EM)
CD8+ CD45RA+ Effector Memory (EMRA)
CD8+ EM
CD8+ CM
Mucosal-Asociated Invariant T cells (MAIT)/NKT
Regulatory T cells (Treg)
γδ- non-MAIT/NKT CD4-CD8-
γδ+
CD4+ EMRA
N
More abundant in HC subjects
% of non-neoplastic T cells
γδ+ Naive 0.028
CD4+CD28+ KLRG1+ EM 0.039
CD4-CD8- MAIT/NKT 0.061
CD4-CD8- KLRG1+ MAIT/NKT 0.023
CD8+ KLRG1+ MAIT/NKT 0.061
More abundant in CTCL subjects
CD4+CLA+ CD39+ CM 0.027
CD4+CLA+ CD39+ Trcm 0.023
CLA+CD39+Tfr 0.039
CD4-CD8- CM 0.039
CD8+CLA+ CD39+ CM 0.087
CLA+ Tfh 0.027
HC MF SS
Status

Figure S1: Quality control, clustering, and disease status differences – related to Figure 1

A) Subject age by disease status. Crossbar indicates mean. Kruskal-Wallis.
B) Subject age by response. Crossbar indicates mean. Wilcoxon rank sum.
C) Subject age by response and disease status. Crossbar indicates mean. FDR-corrected Wilcoxon rank sum.
D) Subject gender by disease status. Fisher's exact.
E) Subject gender by response. Fisher's exact.
F) Subject gender by response and disease status. FDR-corrected Fisher's exact.
G) Exemplative batch correction of three protein channels. Violins represent cell distributions from a common control sample run with each batch.
H) Median expression of proteins by cell types from an equal sampling of subjects at baseline. Canonical markers highlighted in red.
I) UMAPs of individual CTCL subjects in this study (top row) or a public scRNAseq dataset (middle, bottom rows) colored by cell type (top, middle rows) or by the detection of the neoplastic TCR clone (bottom row).
J) % neoplastic T cells of PBMCs or of total T cells by clinical flow cytometry, mass cytometry, and TCR sequencing. Fitting lines calculated by linear regression. R- and p-values calculated by Pearson method.
K) Evenness and richness scores of BL TCR repertoires by status with or without neoplastic T cell clones. Wilcoxon rank sum.
L) BL TCR counts as calculated by Adaptive Biotechnology template counting by disease status. Crossbar indicates mean. Wilcoxon rank sum.
M) Median expression of proteins by non-neoplastic T cell clusters, from an equal sampling of subjects at BL.
N) Percent of T cell clusters out of non-neoplastic T cells at baseline, separated by disease status. Crossbar indicates mean. FDR-corrected Kruskal-Wallis.

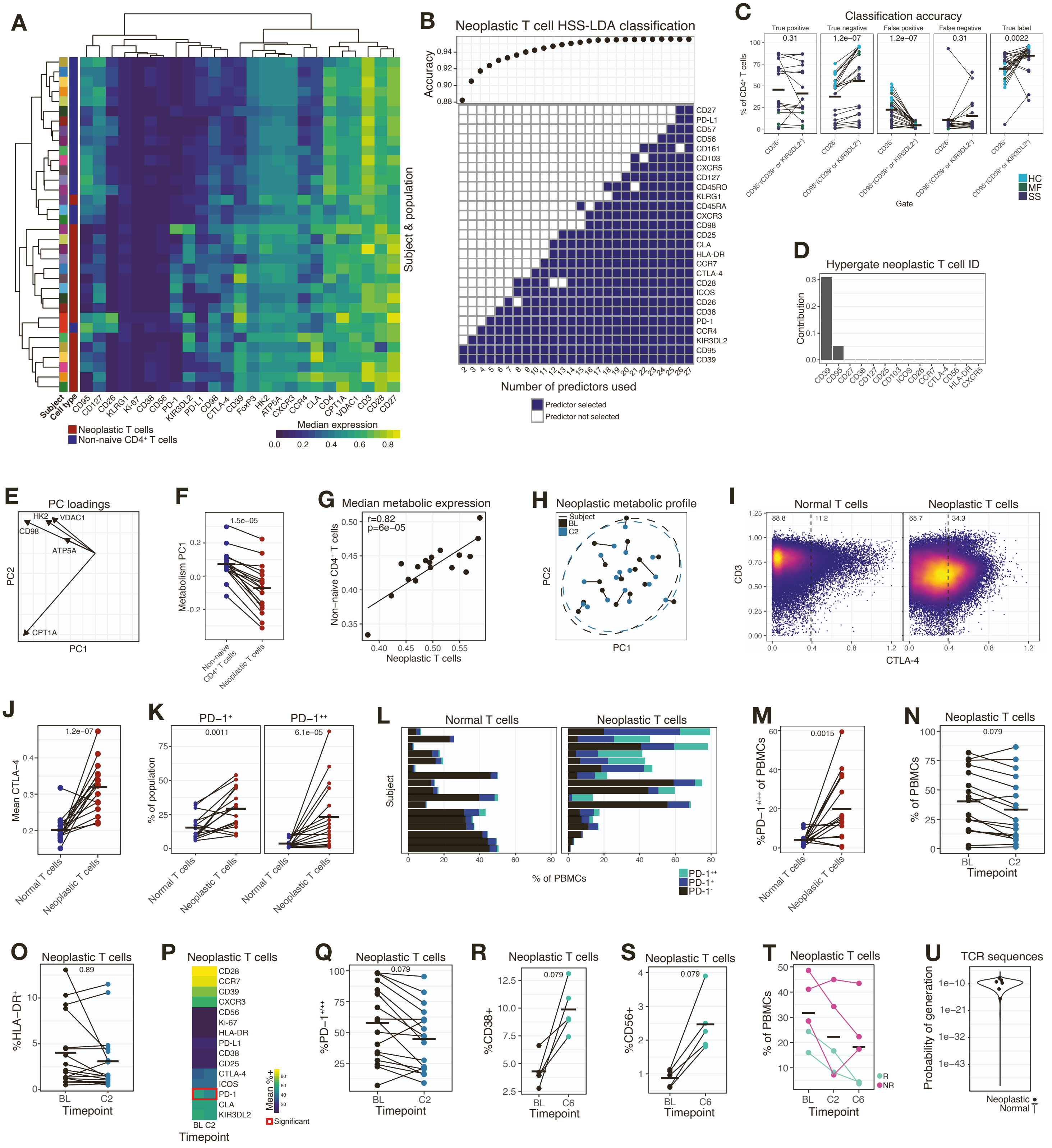
A
B
Neoplastic T cell HSS-LDA classification
Number of predictors used
Predictor selected
Predictor not selected
C
Classification accuracy
D
Hypergate neoplastic T cell ID
E
PC loadings
F
G
Median metabolic expression
H
Neoplastic metabolic profile
I
Normal T cells
Neoplastic T cells
J
K
L
M
N
O
P
Q
R
S
T
U
TCR sequences

Figure S2: Neoplastic T cell phenotype and response to therapy – related to Figure 2

A) Median expression of differential proteins by non-naïve CD4+ and neoplastic T cells by subject.
B) Predictors included in HSS-LDA models to separate neoplastic and normal CD4+ T cells in equal sampling of HC and leukemic subjects (bottom) and model accuracy (top).
C) Per subject accuracy metrics for manual gating schemes shown in Fig. 2C. Crossbar indicates mean. Wilcoxon signed rank.
D) Marker contribution quantified by Hypergate in model trained to separate neoplastic and normal CD4+ T cells in equal in equal sampling of HC and leukemic subjects.
E) PC loadings of PCA manifold from Fig. 2E
F) PC1 coordinates by cell type; same manifold as Fig. 2E. Crossbar indicates mean. Wilcoxon signed rank.
G) Mean of median expression of metabolic markers for non-naïve $CD4^+$ and neoplastic T cells. Fitting line calculated by linear regression. R- and p-value calculated by Pearson method.
H) PCA coordinates of neoplastic T cells, colored by timepoint; same manifold as Fig. 2E. Ellipses indicate 95th percentile CI assuming a multivariate t-distribution.
I) Expression of equal sampling of normal or neoplastic T cells at BL, colored by density. Numbers indicate percent in gate.
J) Mean CTLA-4 expression in normal and neoplastic T cells. Crossbar indicates mean. Wilcoxon signed rank.
K) % PD-1^+ and %PD-1^{++} normal or neoplastic T cells. Crossbar indicates mean. FDR-corrected Wilcoxon signed rank.
L) %PD-1 expression level by normal or neoplastic T cells of total PBMCs.
M) %PD-$1^{+/++}$ normal or neoplastic T cells of total PBMCs. Crossbar indicates mean. Wilcoxon signed rank.
N) % neoplastic T cells of PBMCs by timepoint. Crossbar indicates mean. Wilcoxon signed rank.
O) %HLA-DR^+ neoplastic T cells by timepoint. Crossbar indicates mean. FDR-adjusted Wilcoxon signed rank.
P) Mean %+ of activation markers in neoplastic T cells by timepoint. Red box indicates significance. FDR-corrected Wilcoxon signed rank.
Q) %PD-$1^{+/++}$ neoplastic T cells by timepoint. Crossbar indicates mean. Wilcoxon signed rank.
R) %$CD38^+$ neoplastic T cells by timepoint for subjects with late timepoint data. Crossbar indicates mean. FDR-adjusted Wilcoxon signed rank.
S) %$CD56^+$ neoplastic T cells by timepoint for subjects with late timepoint data. Crossbar indicates mean. FDR-adjusted Wilcoxon signed rank.
T) % neoplastic T cells of total PBMCs by timepoint for subjects with late timepoint data colored by therapeutic response. Crossbar indicates mean.
U) Probably of generation of TCR sequence for neoplastic TCRs (dots) and total normal repertoires (violin). Distributions were not significantly different (p=0.533, Wilcoxon rank sum).

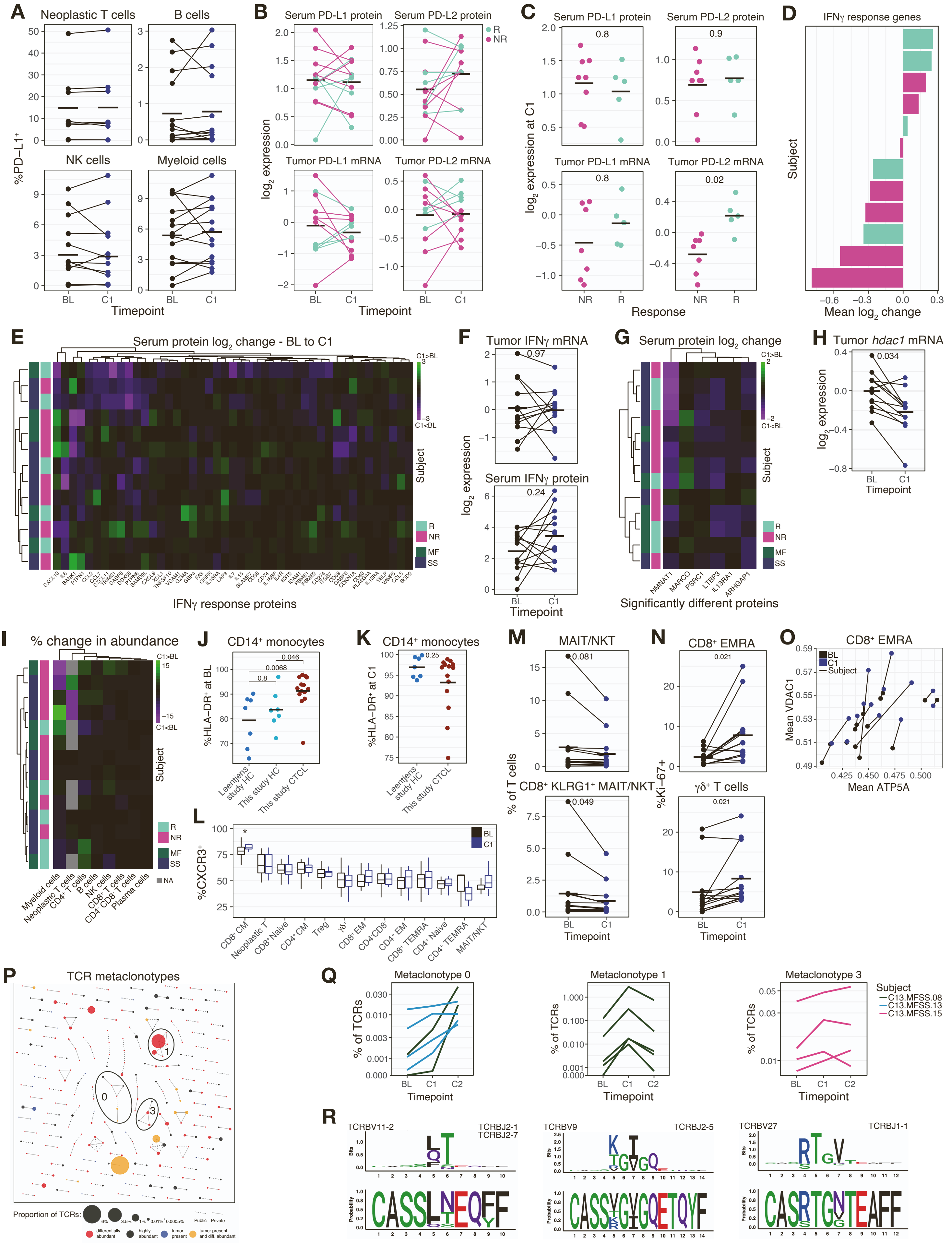
A
Neoplastic T cells
B cells
NK cells
Myeloid cells
%PD−L1⁺
BL
C1
Timepoint
B
Serum PD-L1 protein
Serum PD-L2 protein
Tumor PD-L1 mRNA
Tumor PD-L2 mRNA
R
NR
log2 expression
C
log2 expression at C1
Response
D
IFNγ response genes
Subject
Mean log2 change
E
Serum protein log2 change - BL to C1
C1>BL
C1<BL
Subject
MF
SS
IFNγ response proteins
F
Tumor IFNγ mRNA
Serum IFNγ protein
G
Serum protein log2 change
NMNAT1
MARCO
PSRC1
LTBP3
IL13RA1
ARHGAP1
Significantly different proteins
H
Tumor hdac1 mRNA
I
% change in abundance
Myeloid cells
Neoplastic T cells
CD4⁺ T cells
B cells
NK cells
CD8⁺ T cells
CD4⁻CD8⁻ T cells
Plasma cells
NA
J
CD14⁺ monocytes
%HLA−DR⁺ at BL
Leentjens study HC
This study HC
This study CTCL
K
CD14⁺ monocytes
%HLA−DR⁺ at C1
L
%CXCR3⁺
CD8⁺ CM
Neoplastic T
CD8⁺ Naive
CD4⁺ CM
Treg
γδ⁺
CD8⁺ EM
CD4⁻CD8⁻
CD4⁺ EM
CD8⁺ TEMRA
CD4⁺ Naive
CD4⁺ TEMRA
MAIT/NKT
M
MAIT/NKT
CD8⁺ KLRG1⁺ MAIT/NKT
% of T cells
N
CD8⁺ EMRA
γδ⁺ T cells
%Ki−67+
O
CD8⁺ EMRA
Subject
Mean VDAC1
Mean ATP5A
P
TCR metaclonotypes
Proportion of TCRs:
Public
Private
differentially abundant
highly abundant
tumor present
tumor present and diff. abundant
Q
Metaclonotype 0
Metaclonotype 1
Metaclonotype 3
% of TCRs
C2
C13.MFSS.08
C13.MFSS.13
C13.MFSS.15
R
TCRBV11-2
TCRBJ2-1
TCRBJ2-7
TCRBV9
TCRBJ2-5
TCRBV27
TCRBJ1-1
Bits
Probability

Figure S3: *In vivo* effects of interferon-gamma – related to Figure 3

A) %PD-L1$^+$ cells by cell type and timepoint. Crossbar indicates mean. Differences across timepoints were not significant ($q>0.1$). FDR-corrected Wilcoxon signed rank.
B) Log_2 expression of serum proteins and tumor mRNA by timepoint and colored by response. Crossbar indicates mean. Differences across timepoints were not significant ($q>0.1$). FDR-corrected Wilcoxon signed rank.
C) Log_2 expression of serum proteinsand tumor mRNA at C1 by therapeutic response. Crossbar indicates mean. FDR-corrected Wilcoxon rank sum.
D) Mean log_2 change in expression of IFNg response genes in tumor mRNA between BL and C1, by subject, colored by response.
E) Log_2 change in expression of IFNg response genes in serum proteins between BL and C1, by subject.
F) Log_2 expression of serum protein and tumor mRNA by timepoint. Crossbar indicates mean. Wilcoxon signed rank.
G) Log_2 change in expression of serum proteins between BL and C1, by subject. All proteins were significantly differentially expressed ($p<0.05$ & $|\text{effect size}|>1$). Wilcoxon signed rank.
H) Log_2 expression of tumor mRNA by timepoint. Crossbar indicates mean. Wilcoxon signed rank; $|\text{effect size}|>1$.
I) Difference in cell type abundance between BL and C1. Differences across timepoints were not significant ($q>0.1$). FDR-corrected Wilcoxon signed rank.
J) %HLA-DR$^+$ of CD14$^+$ monocytes at BL by cohort and disease status. Crossbar indicates mean. FDR-corrected Wilcoxon rank sum.
K) %HLA-DR$^+$ of CD14$^+$ monocytes at C1 by cohort and disease status. Crossbar indicates mean. Wilcoxon rank sum.
L) %CXCR3$^+$ of T cell populations, separated by timepoint. * indicates $q<0.1$. FDR-corrected Wilcoxon signed rank. Boxplots represent interquartile range (IQR); horizontal line indicates median; whiskers extend to the farthest data point within 1.5 × IQR.
M) % T cell populations of non-neoplastic T cells by timepoint. Crossbar indicates mean. FDR-corrected Wilcoxon signed rank.
N) % Ki-67$^+$ of T cell populations by timepoint. Crossbar indicates mean. FDR-corrected Wilcoxon signed rank.
O) Mean expression of metabolic markers by CD8$^+$ EMRA T cells across timepoints. Both markers were significantly differentially expressed between timepoints ($q<0.1$). FDR-corrected Wilcoxon signed rank.
P) TCR clones, colored by indicated criteria, organized into metaclones.
Q) % of TCRs by clone, colored by subject, separated by metaclone.
R) V and J gene usage and CDR3 sequence motifs of the metaclones indicated in (Q). The top sequence logo shows the information relative to amino acids found in a background set of randomly sampled CDR3 sequences with the same V and J genes.

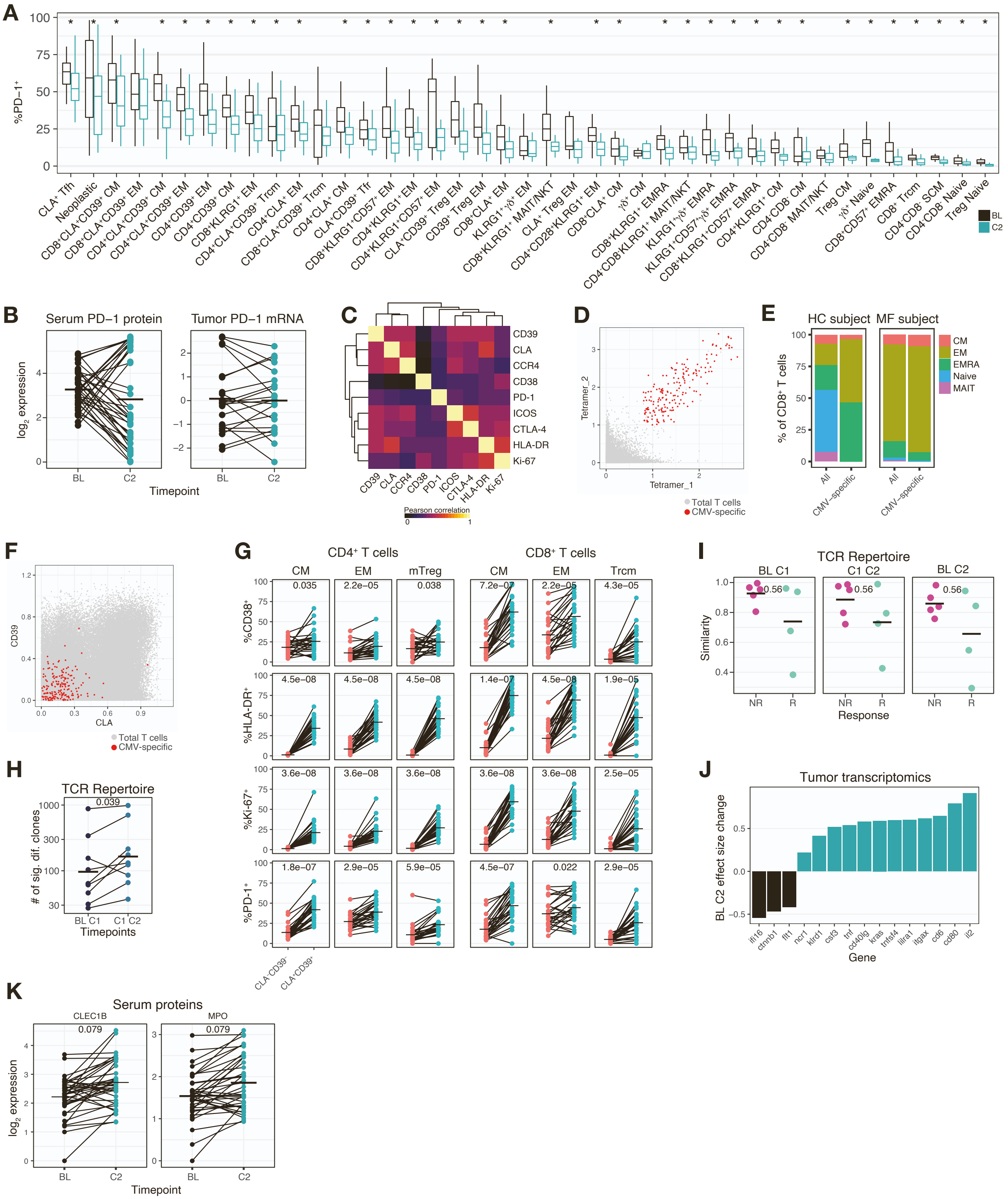

A
%PD-1⁺
CLA⁺ Tfh
Neoplastic
BL
C2
B
Serum PD−1 protein
Tumor PD−1 mRNA
log2 expression
Timepoint
C
CD39
CLA
CCR4
CD38
PD-1
ICOS
CTLA-4
HLA-DR
Ki-67
Pearson correlation
D
Tetramer_2
Tetramer_1
Total T cells
CMV-specific
E
HC subject
MF subject
% of CD8⁺ T cells
CM
EM
EMRA
Naive
MAIT
All
CMV−specific
F
CD39
CLA
Total T cells
CMV-specific
G
CD4⁺ T cells
CD8⁺ T cells
CM
EM
mTreg
Trcm
%CD38⁺
%HLA-DR⁺
%Ki-67⁺
%PD-1⁺
H
TCR Repertoire
# of sig. dif. clones
Timepoints
I
TCR Repertoire
BL C1
C1 C2
BL C2
Similarity
Response
NR
R
J
Tumor transcriptomics
BL C2 effect size change
Gene
K
Serum proteins
CLEC1B
MPO
log2 expression
Timepoint

Figure S4: *In vivo* effects of anti-PD-1 – related to Figure 4

A) %PD-1^+ T cell populations, separated by timepoint. * indicates $q<0.1$. FDR-corrected Wilcoxon signed rank. Boxplots represent IQR; horizontal line indicates median; whiskers extend to the farthest data point within 1.5 × IQR.

B) Log_2 expression of serum proteins and tumor mRNA by timepoint. Crossbar indicates mean. Differences across timepoints were not significant ($q>0.1$). FDR-corrected Wilcoxon signed rank.

C) Pearson correlation between proteins expressed on non-neoplastic T cells at C2, post-anti-PD-1 treatment.

D) Exemplative staining of T cells from an equal sampling of non-neoplastic T cells pre- and post-anti-PD-1 treatment.

E) Composition of T cell subsets comprising total $CD8^+$ T cells and CMV-specific $CD8^+$ T cells in two subjects.

F) Exemplative staining of T cells from an equal sampling of non-neoplastic T cells pre- and post-anti-PD-1 treatment.

G) $\%^+$ activation markers across T cell populations by timepoint. FDR-corrected Wilcoxon signed rank.

H) The number of significantly differentially abundant clones ($q<0.1$) between indicated timepoints as calculated by FDR-corrected Fisher's exact test. P-value calculated from Wilcoxon signed rank test.

I) One minus the Horn-Morista index of the non-neoplastic TCR repertoire similarity of CTCL subjects at indicated timepoints by response. Crossbar indicates mean. FDR-corrected Wilcoxon rank sum.

J) Effect size difference for genes not significantly differentially expressed after multiple hypothesis correction ($p<0.05$; $q>0.1$) between BL and C2. Uncorrected/FDR-corrected Wilcoxon signed rank.

K) Log_2 expression of serum proteins significantly differentially expressed ($q<0.1$) between BL and C2. Crossbar indicates mean. FDR-corrected Wilcoxon signed rank.

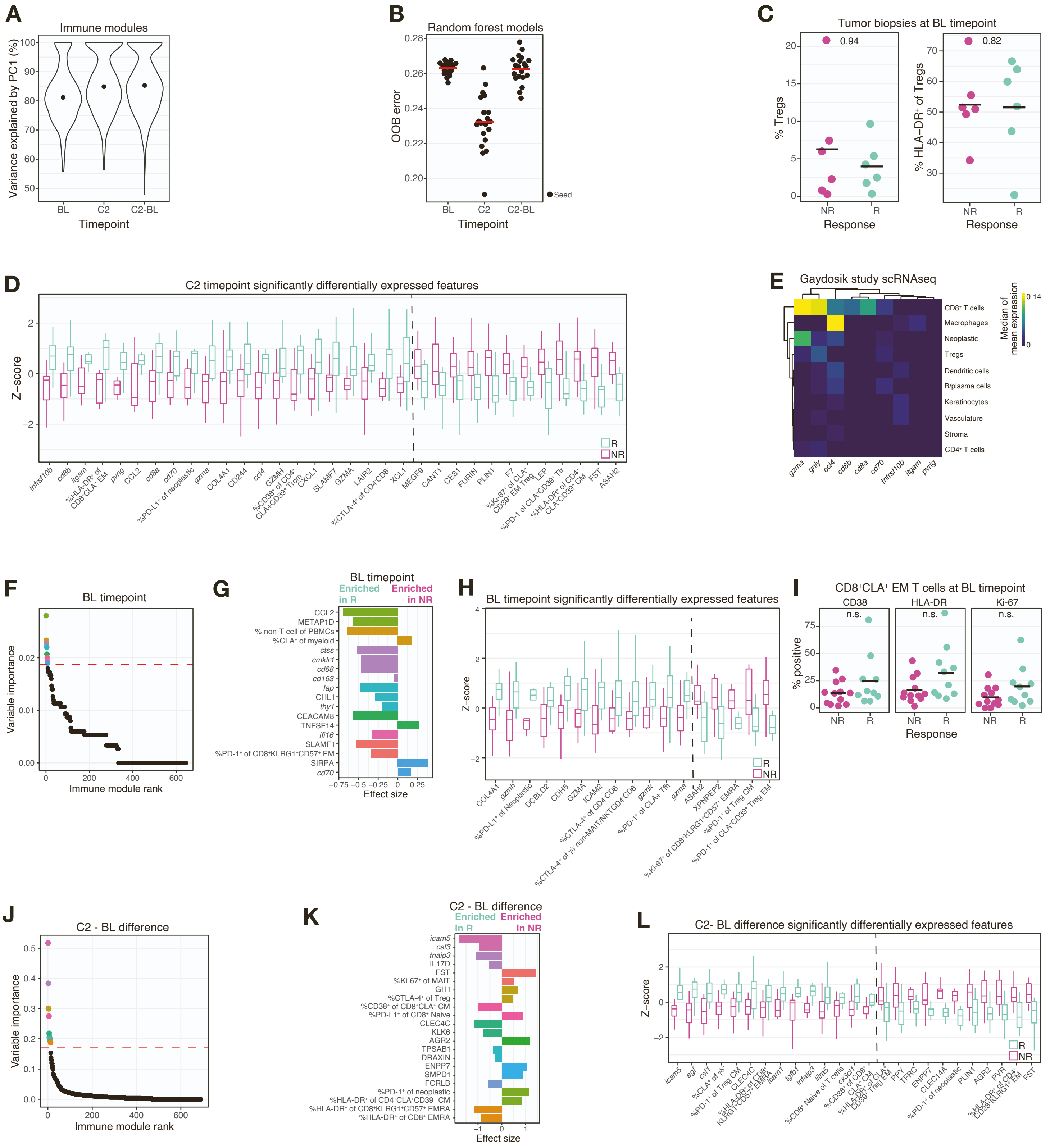
A
Immune modules
Variance explained by PC1 (%)
Timepoint
BL
C2
C2-BL
B
Random forest models
OOB error
Seed
C
Tumor biopsies at BL timepoint
% Tregs
% HLA−DR+ of Tregs
0.94
0.82
NR
R
Response
D
C2 timepoint significantly differentially expressed features
Z−score
tnfrsf10b
cd8b
itgam
%HLA-DR+ of CD8+CLA+ EM
pvrig
CCL2
cd8a
cd70
%PD-L1+ of neoplastic
gzma
COL4A1
CD244
ccl4
GZMH
%CD38+ of CD4+ CLA+CD39+ Tcm
CXCL1
SLAMF7
GZMA
LAIR2
%CTLA-4+ of CD4 CD8
XCL1
MEGF9
CANT1
CES1
FURIN
PLIN1
F7
%Ki-67+ of CLA+ CD39+ EM Treg
%PD-1 of CLA+CD39+ Tfr
LEP
%HLA-DR+ of CD4+ CLA+CD39+ CM
FST
ASAH2
R
NR
E
Gaydosik study scRNAseq
CD8+ T cells
Macrophages
Neoplastic
Tregs
Dendritic cells
B/plasma cells
Keratinocytes
Vasculature
Stroma
CD4+ T cells
gzma
gnly
ccl4
cd8b
cd8a
cd70
tnfrsf10b
itgam
pvrig
Median of mean expression
F
BL timepoint
Variable importance
Immune module rank
G
BL timepoint
Enriched in R
Enriched in NR
CCL2
METAP1D
% non-T cell of PBMCs
%CLA+ of myeloid
ctss
cmklr1
cd68
cd163
fap
CHL1
thy1
CEACAM8
TNFSF14
ifi16
SLAMF1
%PD-1+ of CD8+KLRG1+CD57+ EM
SIRPA
cd70
Effect size
H
BL timepoint significantly differentially expressed features
Z−score
COL4A1
gzmh
%PD-L1+ of Neoplastic
DCBLD2
CDH5
GZMA
ICAM2
%CTLA-4+ of CD4 CD8
%CTLA-4+ of γδ non-MAIT/NKT CD4 CD8
gzmk
%PD-1+ of CLA+ Tfr
gzma
ASAH2
XPNPEP2
%Ki-67+ of CD8+KLRG1+CD57+ EMRA
%PD-1+ of Treg CM
%PD-1+ of CLA+CD39+ Treg EM
R
NR
I
CD8+CLA+ EM T cells at BL timepoint
CD38
HLA-DR
Ki-67
n.s.
% positive
NR
R
Response
J
C2 - BL difference
Variable importance
Immune module rank
K
C2 - BL difference
Enriched in R
Enriched in NR
icam5
csf3
tnaip3
IL17D
FST
%Ki-67+ of MAIT
GH1
%CTLA-4+ of Treg
%CD38+ of CD8+CLA+ CM
%PD-L1+ of CD8+ Naive
CLEC4C
KLK6
AGR2
TPSAB1
DRAXIN
ENPP7
SMPD1
FCRLB
%PD-1+ of neoplastic
%HLA-DR+ of CD4+CLA+CD39+ CM
%HLA-DR+ of CD8+KLRG1+CD57+ EMRA
%HLA-DR+ of CD8+ EMRA
Effect size
L
C2- BL difference significantly differentially expressed features
Z−score
icam5
egf
csf1
%CLA+ of γδ
%PD-1+ of Treg CM
CLEC4C
%HLA-DR+ of CD8+ KLRG1+CD57+ EMRA
icam1
tgfb1
tnfaip3
lilra5
%CD8+ Naive of T cells
cx3cl1
%CD38+ of CD8+ CLA+ CM
%HLA-DR+ of CLA+ CD39+ Treg EM
PPY
TFRC
ENPP7
CLEC14A
%PD-1+ of neoplastic
PLIN1
AGR2
PVR
%HLA-DR+ of CD4+ CD28+KLRG1+ EM
FST
R
NR

Figure S5: Identification of immune features stratifying responders and non-responders – related to Figure 5

A) % variance explained by PC1 within each immune module by timepoint. Dots indicate median.

B) Out-of-bag errors across different seeds of the random forest models generated to predict therapeutic response using immune modules by timepoint. Crossbar indicates mean. Lower error indicates improved accuracy.

C) % Tregs of T cells and %Ki-67$^+$ Tregs at BL by response. Crossbar indicates mean. FDR-corrected Wilcoxon rank sum.

D) Z-scores of significantly differentially expressed features ($p<0.05$ & $|effect|>1$) stratifying therapeutic response at C2. Uncorrected Wilcoxon rank sum. Boxplots represent IQR; horizontal line indicates median; whiskers extend to the farthest data point within 1.5 × IQR. Dashed line separates features enriched in responders and features enriched in non-responders.

E) Median of mean expression of genes identified in (D) by cell population.

F) Gini index of immune module predictors separating response and non-response at the BL timepoint. Dashed red line indicates threshold set for immune modules shown in (G). Colors correspond to immune modules in (G).

G) Effect size of underlying features comprising immune modules selected in (F). Bar color denotes immune module membership. Features are ordered by Gini index.

H) Z-scores of significantly differentially expressed features ($p<0.05$ & $|effect|>1$) stratifying therapeutic response at BL. Wilcoxon rank sum. Boxplots represent IQR; horizontal line indicates median; whiskers extend to the farthest data point within 1.5 × IQR. Dashed line separates features enriched in responders and features enriched in non-responders.

I) %$^+$ of activation markers by CD8$^+$CLA$^+$ EM T cells at BL by response. Crossbar indicates mean. No comparison was statistically significant ($q>0.1$). FDR-corrected Wilcoxon rank sum test.

J) Gini index of immune module predictors separating response and non-response in the C2-BL timepoint. Dashed red line indicates threshold set for immune modules shown in K. Colors correspond to immune modules in (K).

K) Effect size of underlying features comprising immune modules selected in (J). Bar color denotes immune module membership. Features are ordered by Gini index.

L) Z-scores of significantly differentially expressed features ($p<0.05$ & $|effect|>1$) stratifying therapeutic response at C2-BL difference. Wilcoxon rank sum. Boxplots represent IQR; horizontal line indicates median; whiskers extend to the farthest data point within 1.5 × IQR. Dashed line separates features enriched in responders and features enriched in non-responders.

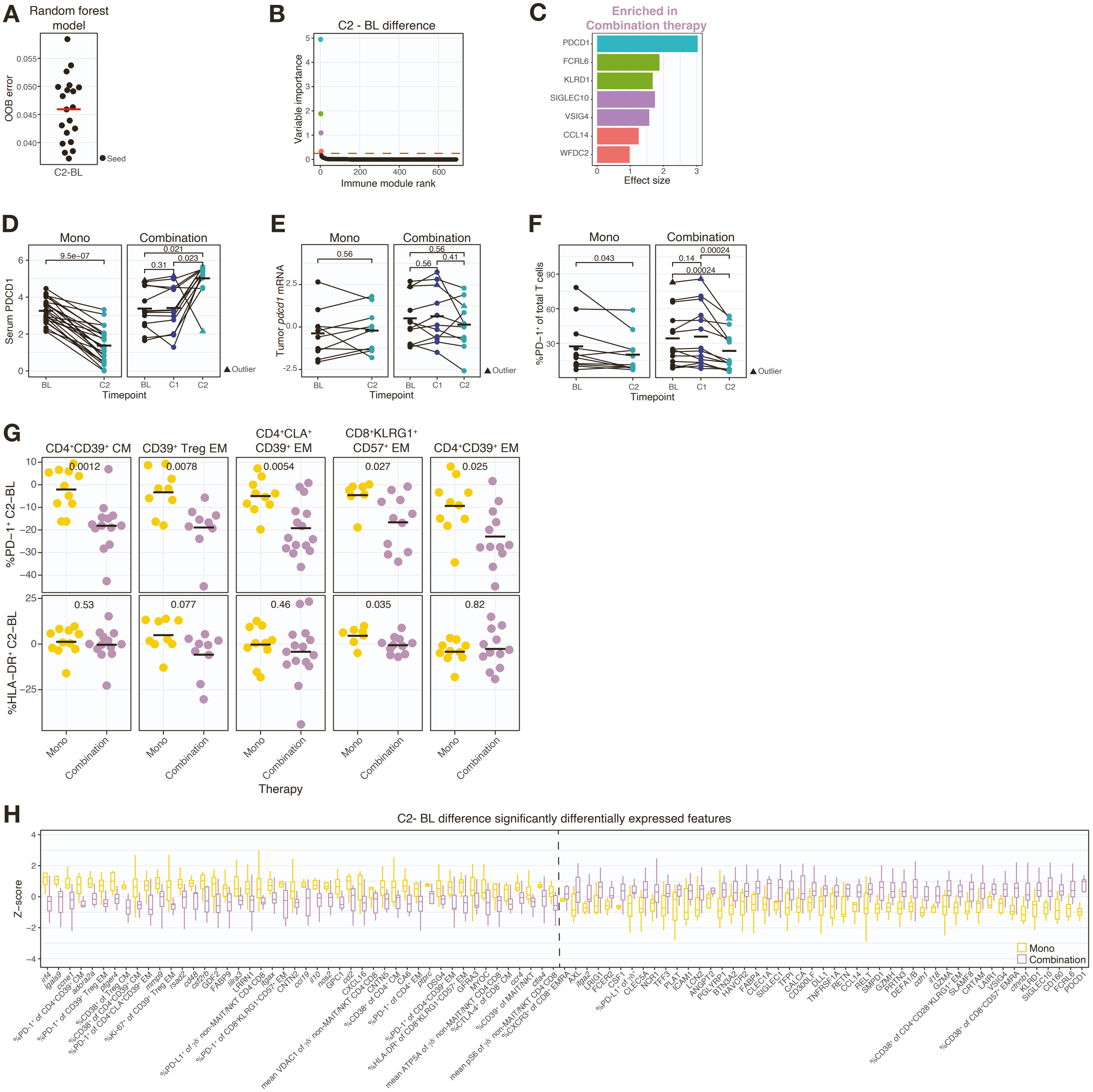

A
Random forest model
OOB error
Seed
C2-BL
B
C2 - BL difference
Variable importance
Immune module rank
C
Enriched in Combination therapy
PDCD1
FCRL6
KLRD1
SIGLEC10
VSIG4
CCL14
WFDC2
Effect size
D
Mono
Combination
Serum PDCD1
9.5e−07
0.021
0.31
0.023
Outlier
BL
C1
C2
Timepoint
E
Mono
Combination
Tumor pdcd1 mRNA
0.56
0.41
Outlier
Timepoint
F
Mono
Combination
%PD−1⁺ of total T cells
0.043
0.14
0.00024
Outlier
Timepoint
G
CD4⁺CD39⁺ CM
CD39⁺ Treg EM
CD4⁺CLA⁺ CD39⁺ EM
CD8⁺KLRG1⁺ CD57⁺ EM
CD4⁺CD39⁺ EM
%PD−1⁺ C2−BL
0.0012
0.0078
0.0054
0.027
0.025
%HLA−DR⁺ C2−BL
0.53
0.077
0.46
0.035
0.82
Mono
Combination
Therapy
H
C2- BL difference significantly differentially expressed features
Z−score
Mono
Combination

Figure S6: Mono and combination therapy induce differential abundance of PD-1 – related to Figure 5

A) Out-of-bag errors across different seeds of the random forest models generated to predict therapy regimen using immune modules at the C2-BL difference. Crossbar indicates mean. Lower error indicates improved accuracy.

B) Gini index of immune module predictors separating mono and combination therapy in the C2-BL timepoint. Dashed red line indicates threshold set for immune modules shown in (C). Colors correspond to immune modules in (C).

C) Effect size of underlying features comprising immune modules selected in (B). Bar color denotes immune module membership. Features are ordered by Gini index.

D) Log_2 expression of serum PD-1 by clinical trial and timepoint. Crossbar indicates mean. FDR-corrected Wilcoxon signed rank.

E) Log_2 expression of tumor *pdcd1* mRNA by clinical trial and timepoint. Crossbar indicates mean. FDR-corrected Wilcoxon signed rank.

F) %PD-1^+ of total T cells by clinical trial and timepoint. Crossbar indicates mean. FDR-corrected Wilcoxon signed rank.

G) Difference in %PD-1^+ (top row) or %HLA-DR^+ (bottom row) between C2 and BL in T cell populations by clinical trial. Crossbar indicates mean. FDR-corrected Wilcoxon rank sum.

H) Z-scores of significantly differentially expressed features ($p<0.05$ & $|effect|>1$) stratifying response to mono and combination therapy at C2-BL difference. Wilcoxon rank sum. Boxplots represent IQR; horizontal line indicates median; whiskers extend to the farthest data point within 1.5 × IQR. Dashed line separates features enriched in monotherapy and features enriched in combination therapy.

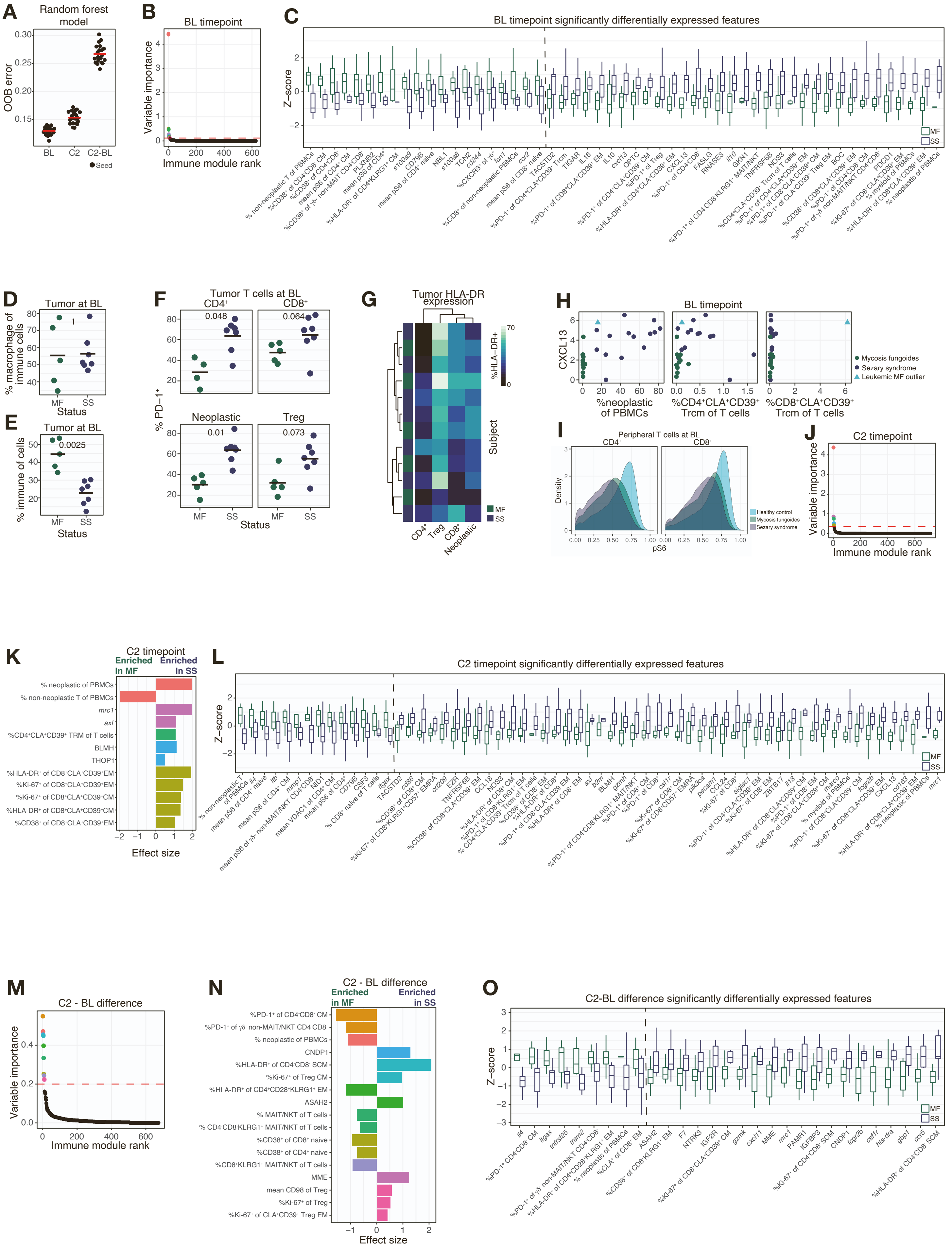
A
Random forest model
OOB error
BL
C2
C2-BL
Seed
B
BL timepoint
Variable importance
Immune module rank
C
BL timepoint significantly differentially expressed features
Z-score
MF
SS
D
Tumor at BL
% macrophage of immune cells
Status
E
Tumor at BL
% immune of cells
0.0025
Status
F
Tumor T cells at BL
CD4+
CD8+
0.048
0.064
Neoplastic
Treg
0.01
0.073
% PD-1+
Status
G
Tumor HLA-DR expression
%HLA-DR+
Subject
CD4+
Treg
CD8+
Neoplastic
MF
SS
H
BL timepoint
CXCL13
%neoplastic of PBMCs
%CD4+CLA+CD39+ Trcm of T cells
%CD8+CLA+CD39+ Trcm of T cells
Mycosis fungoides
Sezary syndrome
Leukemic MF outlier
I
Peripheral T cells at BL
CD4+
CD8+
Density
pS6
Healthy control
Mycosis fungoides
Sezary syndrome
J
C2 timepoint
Variable importance
Immune module rank
K
C2 timepoint
Enriched in MF
Enriched in SS
% neoplastic of PBMCs
% non-neoplastic T of PBMCs
mrc1
axl
%CD4+CLA+CD39+ TRM of T cells
BLMH
THOP1
%HLA-DR+ of CD8+CLA+CD39+EM
%Ki-67+ of CD8+CLA+CD39+EM
%Ki-67+ of CD8+CLA+CD39+CM
%HLA-DR+ of CD8+CLA+CD39+CM
%CD38+ of CD8+CLA+CD39+EM
Effect size
L
C2 timepoint significantly differentially expressed features
Z-score
MF
SS
M
C2 - BL difference
Variable importance
Immune module rank
N
C2 - BL difference
Enriched in MF
Enriched in SS
%PD-1+ of CD4-CD8- CM
%PD-1+ of γδ- non-MAIT/NKT CD4-CD8-
% neoplastic of PBMCs
CNDP1
%HLA-DR+ of CD4-CD8- SCM
%Ki-67+ of Treg CM
%HLA-DR+ of CD4+CD28+KLRG1+ EM
ASAH2
% MAIT/NKT of T cells
% CD4-CD8-KLRG1+ MAIT/NKT of T cells
%CD38+ of CD8+ naive
%CD38+ of CD4+ naive
%CD8+KLRG1+ MAIT/NKT of T cells
MME
mean CD98 of Treg
%Ki-67+ of Treg
%Ki-67+ of CLA+CD39+ Treg EM
Effect size
O
C2-BL difference significantly differentially expressed features
Z-score
MF
SS

Figure S7: Identification of immune features stratifying MF and SS – related to Figure 6

A) Out-of-bag errors across different seeds of the random forest models generated to predict disease status using immune modules, separated by timepoint. Crossbar indicates mean. Lower error indicates improved accuracy.
B) Gini index of immune module predictors separating MF and SS at the BL timepoint. Dashed red line indicates threshold set for immune modules shown in Fig. 6A. Colors correspond to immune modules in Fig. 6A.
C) Z-scores of significantly differentially expressed features ($p<0.05$ & $|effect|>1$) stratifying MF and SS subjects at BL. Wilcoxon rank sum. Boxplots represent IQR; horizontal line indicates median; whiskers extend to the farthest data point within 1.5 × IQR. Dashed line separates features enriched in MF and features enriched in SS.
D) % macrophages of immune cells in tumor samples at BL. Crossbar indicates mean. Wilcoxon rank sum.
E) % immune cells of total cells in tumor samples at BL. Crossbar indicates mean. Wilcoxon rank sum.
F) %PD-1^+ of indicated T cell populations in tumor samples at BL. Crossbar indicates mean. FDR-corrected Wilcoxon rank sum.
G) %HLA-DR^+ of indicated tumor-present T cell populations at BL by subject.
H) CXCL13 serum protein level and abundance of indicated cell populations at BL.
I) Expression of pS6 for indicated T cell populations drawn from an equal sampling of cells from HC, MF, and SS subjects at BL.
J) Gini index of immune module predictors separating MF and SS at the BL timepoint. Dashed red line indicates threshold set for immune modules shown in (K). Colors correspond to immune modules in (K).
K) Effect size of underlying features comprising immune modules selected by model differentiating MF and SS subjects at C2. Bar color denotes immune module membership as in (J). Features are ordered by Gini index.
L) Z-scores of significantly differentially expressed features ($p<0.05$ & $|effect|>1$) stratifying MF and SS at C2. Wilcoxon rank sum. Boxplots represent IQR; horizontal line indicates median; whiskers extend to the farthest data point within 1.5 × IQR. Dashed line separates features enriched in MF and features enriched in SS.
M) Gini index of immune module predictors separating MF and SS at C2-BL difference. Dashed red line indicates threshold set for immune modules shown in (N). Colors correspond to immune modules in (N).
N) Effect size of underlying features comprising immune modules selected by model differentiating MF and SS subjects at C2-BL difference. Bar color denotes immune module membership as in (M). Features are ordered by Gini index.
O) Z-scores of significantly differentially expressed features ($p<0.05$ & $|effect|>1$) stratifying MF and SS subjects at C2-BL difference. Wilcoxon rank sum. Boxplots represent IQR; horizontal line indicates median; whiskers extend to the farthest data point within 1.5 × IQR. Dashed line separates features enriched in MF and features enriched in SS.

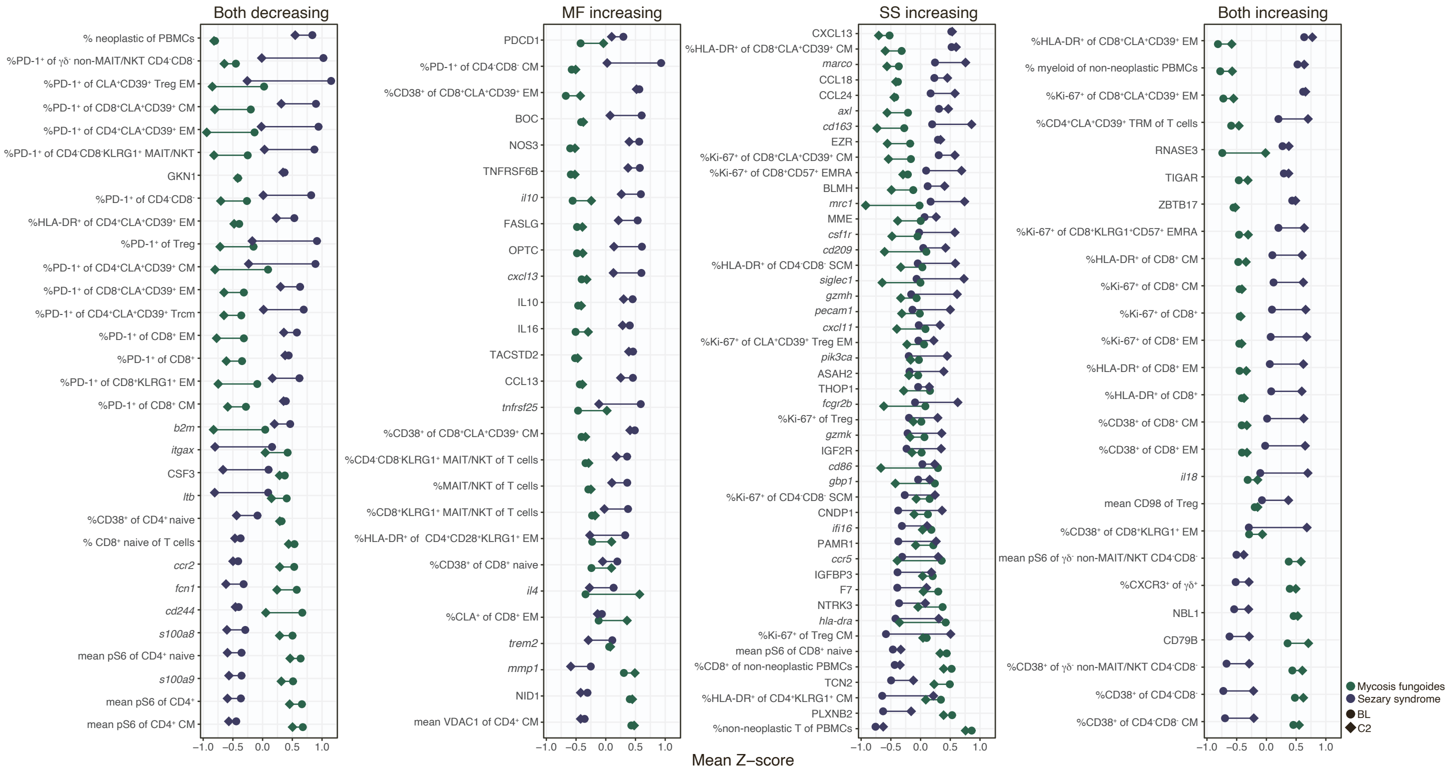

Both decreasing
% neoplastic of PBMCs
%PD-1+ of γδ- non-MAIT/NKT CD4-CD8-
%PD-1+ of CLA+CD39+ Treg EM
%PD-1+ of CD8+CLA+CD39+ CM
%PD-1+ of CD4+CLA+CD39+ EM
%PD-1+ of CD4-CD8-KLRG1+ MAIT/NKT
GKN1
%PD-1+ of CD4-CD8-
%HLA-DR+ of CD4+CLA+CD39+ EM
%PD-1+ of Treg
%PD-1+ of CD4+CLA+CD39+ CM
%PD-1+ of CD8+CLA+CD39+ EM
%PD-1+ of CD4+CLA+CD39+ Trcm
%PD-1+ of CD8+ EM
%PD-1+ of CD8+
%PD-1+ of CD8+KLRG1+ EM
%PD-1+ of CD8+ CM
b2m
itgax
CSF3
ltb
%CD38+ of CD4+ naive
% CD8+ naive of T cells
ccr2
fcn1
cd244
s100a8
mean pS6 of CD4+ naive
s100a9
mean pS6 of CD4+
mean pS6 of CD4+ CM
–1.0 –0.5 0.0 0.5 1.0
MF increasing
PDCD1
%PD-1+ of CD4-CD8- CM
%CD38+ of CD8+CLA+CD39+ EM
BOC
NOS3
TNFRSF6B
il10
FASLG
OPTC
cxcl13
IL10
IL16
TACSTD2
CCL13
tnfrsf25
%CD38+ of CD8+CLA+CD39+ CM
%CD4-CD8-KLRG1+ MAIT/NKT of T cells
%MAIT/NKT of T cells
%CD8+KLRG1+ MAIT/NKT of T cells
%HLA-DR+ of CD4+CD28+KLRG1+ EM
%CD38+ of CD8+ naive
il4
%CLA+ of CD8+ EM
trem2
mmp1
NID1
mean VDAC1 of CD4+ CM
–1.0 –0.5 0.0 0.5 1.0
SS increasing
CXCL13
%HLA-DR+ of CD8+CLA+CD39+ CM
marco
CCL18
CCL24
axl
cd163
EZR
%Ki-67+ of CD8+CLA+CD39+ CM
%Ki-67+ of CD8+CD57+ EMRA
BLMH
mrc1
MME
csf1r
cd209
%HLA-DR+ of CD4-CD8- SCM
siglec1
gzmh
pecam1
cxcl11
%Ki-67+ of CLA+CD39+ Treg EM
pik3ca
ASAH2
THOP1
fcgr2b
%Ki-67+ of Treg
gzmk
IGF2R
cd86
gbp1
%Ki-67+ of CD4-CD8- SCM
CNDP1
ifi16
PAMR1
ccr5
IGFBP3
F7
NTRK3
hla-dra
%Ki-67+ of Treg CM
mean pS6 of CD8+ naive
%CD8+ of non-neoplastic PBMCs
TCN2
%HLA-DR+ of CD4+KLRG1+ CM
PLXNB2
%non-neoplastic T of PBMCs
–1.0 –0.5 0.0 0.5 1.0
Both increasing
%HLA-DR+ of CD8+CLA+CD39+ EM
% myeloid of non-neoplastic PBMCs
%Ki-67+ of CD8+CLA+CD39+ EM
%CD4+CLA+CD39+ TRM of T cells
RNASE3
TIGAR
ZBTB17
%Ki-67+ of CD8+KLRG1+CD57+ EMRA
%HLA-DR+ of CD8+ CM
%Ki-67+ of CD8+ CM
%Ki-67+ of CD8+
%Ki-67+ of CD8+ EM
%HLA-DR+ of CD8+ EM
%HLA-DR+ of CD8+
%CD38+ of CD8+ CM
%CD38+ of CD8+ EM
il18
mean CD98 of Treg
%CD38+ of CD8+KLRG1+ EM
mean pS6 of γδ- non-MAIT/NKT CD4-CD8-
%CXCR3+ of γδ+
NBL1
CD79B
%CD38+ of γδ- non-MAIT/NKT CD4-CD8-
%CD38+ of CD4-CD8-
%CD38+ of CD4-CD8- CM
–1.0 –0.5 0.0 0.5 1.0
Mean Z–score
Mycosis fungoides
Sezary syndrome
BL
C2

Figure S8: Kinetics of immune features stratifying MF and SS – related to Figure 6

Mean Z-scores of differential features identified at BL, C2 or the change from BL to C2, stratified by status (color), timepoint (shape), and feature kinetics (panels). Facets are ordered by BL effect size difference.

Table S1: Cohort demographics – related to Figure 1

subject	age	gender	status	response	study
C10.10	76	M	MF	PR	10
C10.11	61	M	MF	PR	10
C10.15	66	M	SS	SD	10
C10.21	52	M	SS	SD	10
C10.19	72	F	SS	PD	10
C10.08	85	F	SS	SD	10
C10.23	59	M	SS	SD	10
C10.24	66	M	MF	PR	10
C10.07	72	F	SS	CR	10
C10.03	62	M	MF	SD	10
C10.04	75	M	MF	PR	10
C10.13	70	M	SS	SD	10
C10.22	82	M	SS	SD	10
C10.05	47	M	MF	SD	10
C10.09	47	F	MF	PD	10
C10.16	73	M	SS	SD	10
C10.17	44	F	SS	CR	10
C10.12	72	M	SS	PR	10
C10.18	64	M	MF	PD	10
C10.06	67	M	SS	PD	10
C10.14	69	M	SS	PD	10
C10.02	67	M	SS	PD	10
C10.01	63	M	MF	PR	10
C10.20	78	F	SS	PR	10
C13.MFSS.08	72	F	SS	PR	13
C13.MFSS.10	53	M	MF	PR	13
C13.MFSS.13	43	F	MF	SD	13
C13.MFSS.07	55	M	MF	PD	13
C13.MFSS.14	71	F	MF	PD	13
C13.MFSS.15	57	M	MF	PR	13
C13.MFSS.16	70	M	SS	SD	13
C13.MFSS.02	62	F	SS	SD	13
C13.MFSS.05	74	M	SS	SD	13
C13.MFSS.09	56	M	SS	SD	13

C13.MFSS.12	56	M	MF	PR	13
C13.MFSS.01	63	F	SS	CR	13
C13.MFSS.03	73	M	SS	PD	13
C13.MFSS.04	76	M	MF	CR	13
C13.MFSS.06	56	M	SS	SD	13
HC.02	23	F	HC		
HC.17	47	M	HC		
HC.20	37	M	HC		
HC.23	39	F	HC		
HC.52	26	F	HC		
HC.71	32	F	HC		
HC.81	40	M	HC		

Table S3: Mass cytometry panel – related to Figure 1

Mass	Element	Marker	Localization	Clone	Company	Cat	Stain (ug/test)
89	Y	Barcode_1	Pre-stain	HI30	Standard Bio-tools	3089003B	0.2
103	Rh	DNA	Intracellular	-	Standard Bio-tools	201103A	NA
106	Cd	Barcode_2	Pre-stain	HI30	Standard Bio-tools	3106001B	0.2
110	Cd	Barcode_3	Pre-stain	HI30	Standard Bio-tools	3110001B	0.2
111	Cd	Barcode_4	Pre-stain	HI30	Standard Bio-tools	3111001B	0.2
112	Cd	CD8	Surface	RPA-T8	BioLegend	301002	0.05
113	Cd	CD14	Surface	M5E2	BioLegend	301802	0.8
114	Cd	Barcode_5	Pre-stain	HI30	Standard Bio-tools	3114001B	0.2
115	In	CD57	Surface	HNK-1	BioLegend	359602	0.4
116	Cd	Barcode_6	Surface	HI30	Standard Bio-tools	3116001B	0.2
140	Ce	CD56	Surface	REA196	Miltenyi Biotec	130-108-016	0.025
141	Pr	CLA	Surface	HECA-452	BioLegend	321302	0.2
142	Nd	HLA-DR	Surface	L243	BioLegend	307602	0.8
143	Nd	CXCR5	Surface	J252D4	BioLegend	356902	0.4
144	Nd	CD4	Surface	OKT4	BioLegend	317402	0.8
145	Nd	CD45RA	Surface	HI100	BioLegend	304102	0.05
146	Nd	CD103	Surface	Ber-ACT8	BioLegend	350202	0.2
147	Sm	ICOS	Surface	C398.4A	BioLegend	313502	0.2
148	Nd	CD45RO	Surface	UCHL1	BioLegend	304202	0.8

149	Sm	CCR4	Surface	L291H4	BioLegend	359402	0.2
150	Nd	CD38	Surface	HIT2	BioLegend	303502	0.4
151	Eu	CD127	Surface	QA18A44	BioLegend	375702	0.8
152	Sm	IgG4	Surface	HP6025	Southern Biotech	9200-01	0.1
153	Eu	CD161	Surface	W18070C	BioLegend	307502	0.8
154	Sm	CD26	Surface	BA5b	BioLegend	302702	0.1
155	Gd	Ki-67	Intracellular	Ki-67	BioLegend	350502	0.05
156	Gd	TCRgd	Surface	B1	BioLegend	331202	0.8
157	Gd	CXCR3	Surface	G025H7	BioLegend	353702	0.8
158	Gd	CD27	Surface	M-T271	BioLegend	356401	0.4
159	Tb	VDAC1	Intracellular	20B12AF2	Abcam	ab14734	0.1
160	Gd	CD95	Surface	EOS9.1	BioLegend	305702	0.4
161	Dy	Tetramer	Pre-stain	-		-	0.1
162	Dy	CD25	Surface	BC96	BioLegend	302602	0.4
163	Dy	Tetramer	Pre-stain	-		-	0.1
164	Dy	PD-L1	Surface	29E.2A3	BioLegend	329702	0.2
165	Ho	CTLA-4	Intracellular	BNI3	BioLegend	369602	0.2
166	Er	KIR3DL2	Surface	539304	R&D systems	MAB2878	0.8
167	Er	Tetramer	Pre-stain	-		-	0.1
168	Er	CCR7	Surface	G043H7	BioLegend	353202	0.8
169	Tm	HK2	Intracellular	EPR20839	Abcam	ab209847	0.8
170	Er	Tetramer	Pre-stain	-		-	0.1
171	Yb	FoxP3	Intracellular	206D	BioLegend	320102	0.8
172	Yb	CD39	Surface	A1	BioLegend	328202	0.4
173	Yb	CD28	Surface	CD28.2	BioLegend	302902	0.8
174	Yb	ATP5A	Intracellular	15H4C4	Abcam	ab14748	0.8
175	Lu	pS6	Intracellular	N7-548	Standard Bio-tools	3175009A	0.8
176	Yb	CPT1A	Intracellular	8F6AE9	Abcam	ab128568	0.2
194	Pt	Lamin A/C	Intracellular	E-1	Santa Cruz Biotechnology	sc-376248	0.05
195	Pt	Viability	Pre-stain	-	Standard Bio-tools	201195	NA
196	Pt	KLRG1	Surface	13F12F2	Invitrogen	16-9488-85	0.8
198	Pt	CD98	Surface	UM7F8	BD Pharmingen	556074	0.4
209	Bi	CD3	Surface	UCHT1	BioLegend	300402	0.2
-	-	Pembrolizumab	Pre-stain	-	Selleckchem	A2005	0.5

Table S4: Mass cytometry peptides used in pMHC tetramers – related to Figure 1

Name	Sequence	Molecular weight (g/mol)	Species	HLA	Protein	Length	NetMHCpan EL 4.1 Score	Channel 1	Channel 2
A01_CMV_UL44_1	VTEHDTLLY	1090	CMV	A*01:01	UL44	9	0.996741	Dy161	Dy163
A01_CMV_pp65_1	YSEHPTFTSQY	1359	CMV	A*01:01	pp65	11	0.979006	Dy161	Er167
A01_InfA_NP_1	CTELKLSDY	1071	Influenza A	A*01:01	NP	9	0.803383	Dy161	Er170
A01_InfA_PB1_1	VSDGGPNLY	921	Influenza A	A*01:01	PB1	9	0.996282	Dy163	Er167
A01_HSV1_UL46_1	ATDSLNNEY	1026	HSV1	A*01:01	UL46	9	0.995817	Dy163	Er170
A01_HSV1_UL48_1	FTDALGIDEY	1143	HSV1	A*01:01	UL48	9	0.991648	Er167	Er170
A02_InfA_M1_1	GILGFVFTL	966	Influenza A	A*02:01	M1	9	0.991397	Dy161	Dy163
A02_EBV_BRLF1_1	YVLDHLIVV	1070	EBV	A*02:01	BRLF1	9	0.97253	Dy161	Er167
A02_EBV_BALF4_1	FLDKGTYTL	1057	EBV	A*02:01	BALF4	9	0.996447	Dy161	Er170
A02_CMV_pp65_1	NLVPMVATV	943	CMV	A*02:01	pp65	9	0.832363	Dy163	Er167
A02_CMV_IE1_1	VLEETSVML	1020	CMV	A*02:01	IE1	9	0.757418	Dy163	Er170
A02_HSV1_UL25_1	FLWEDQTLL	1164	HSV1	A*02:01	UL25	9	0.991397	Er167	Er170
A03_EBV_EBNA-3A_1	RLRAEAQVK	1070	EBV	A*03:01	EBNA-3A	9	0.788046	Dy161	Dy163
A03_CMV_IE1_1	KLGGALQAK	885	CMV	A*03:01	IE1	9	0.886184	Dy161	Er167
A03_HSV1_RS1_1	RLYPDAPPLR	1197	HSV1	A*03:01	RS1	9	0.925983	Dy161	Er170
B07_CMV_pp65_1	RPHERNGFTVL	1325	CMV	B*07:02	pp65	12	0.984307	Dy163	Er167
B07_EBV_BMRF1_1	RPQGGSRPEF	1130	EBV	B*07:02	BMRF1	10	0.951012	Dy163	Er170
B07_InfA_PB1_1	QPEWFRNVL	1188	Influenza A	B*07:02	PB1	9	0.911315	Er167	Er170

Table S5: Neoplastic TCRs – related to Figure 2

CDR3	v_gene	j_gene
CSAYTTGAREETQYF	TRBV20-1	TRBJ2-5
CSARDGRGGRNEQFF	TRBV20-1	TRBJ2-1
CSASLGDERGGARAYNEQFF	TRBV20-1	TRBJ2-1
CASRATYEQYF	TRBV6-1	TRBJ2-7
CASSVTSGQETQYF	TRBV5-1	TRBJ2-5
CASSQRDNSPLHF	TRBV3-1	TRBJ1-6
CSASPLAGGRDTGELFF	TRBV20-1	TRBJ2-2
CASSPPPGLADTQYF	TRBV18	TRBJ2-3
CASTPGGARTEAFF	TRBV19	TRBJ1-1
CATGWNTGELFF	TRBV7-9	TRBJ2-2
CASSLAGGHETQYF	TRBV7-2	TRBJ2-5
CASSLVPGVGNQPQHF	TRBV7-9	TRBJ1-5
CASSGRPGPGIPVGEQYF	TRBV2	TRBJ2-7
